# Type, severity, frequency and management of adverse reactions associated with ultrasound contrast agents: a systematic review and meta-analysis

**DOI:** 10.1007/s00330-026-12594-5

**Published:** 2026-05-15

**Authors:** Honoria Ocagli, Laura Romanini, Adea Llane, Stefano di Nola, Aart J. van der Molen, Marie-France Bellin, Torkel Brismar, Jean-Michel Correas, Katerina Deike, Ilona A. Dekkers, Remy W. F. Geenen, Gertraud Heinz, Andreas H. Mahnken, Carlo A. Mallio, Carlo C. Quattrocchi, Alexander Radbruch, Peter Reimer, Giles Roditi, Carmen Sebastià, Olivier Clément, Dario Gregori, Michele Bertolotto

**Affiliations:** 1 Unit of Biostatistics, Epidemiology and Public Health, Department of Cardiac, Thoracic, Vascular Sciences, and Public Health, University of Padova, Padova, Italy; 2 Department of Radiology, ASST Cremona, Cremona, Italy; 3 Epistudia, Bern, Switzerland; 4 Department of Radiology, Leiden University Medical Center, Leiden, The Netherlands; 5 University Paris Saclay, AP-HP, University Hospital Bicêtre, Department of Radiology, BioMaps, Le Kremlin-Bicêtre, France; 6 Department of Clinical Science, Intervention and Technology, Unit of Radiology, Karolinska Institute and Department of Radiology, Karolinska University Hospital in Huddinge, Stockholm, Sweden; 7 Université de Paris, AP-HP, Groupe Hospitalier Necker, DMU Imagina, Service de Radiologie, Paris, France; 8 Clinic for Diagnostic and Interventional Neuroradiology, University Clinic Bonn, and German Center for Neurodegenerative Diseases, DZNE, Bonn, Germany; 9 Athinoula A. Martinos Center for Biomedical Imaging, Massachusetts General Hospital, Charlestown, MA USA; 10 Department of Radiology, Amsterdam UMC, Amsterdam, The Netherlands; 11 Department of Radiology, Northwest Clinics, Alkmaar, The Netherlands; 12 Department of Radiology, Landesklinikum St Pölten, St Pölten, Austria; 13 Department of Diagnostic and Interventional Radiology, Marburg University Hospital, Marburg, Germany; 14 Fondazione Policlinico and Research Unit Radiology, Universitario Campus Bio-Medico, Roma, Italy; 15 Centre for Medical Sciences CISMed, University of Trento, Trento, Italy; 16 Department of Radiology, Institute for Diagnostic and Interventional Radiology, Klinikum Karlsruhe, Karlsruhe, Germany; 17 Department of Radiology, Glasgow Royal Infirmary, Glasgow, UK; 18 Department of Radiology, Hospital Clinic de Barcelona, Barcelona, Spain; 19 Université de Paris, AP-HP, Hôpital Européen Georges Pompidou, DMU Imagina, Service de Radiologie, Paris, France; 20 Department of Radiology, University of Trieste, Trieste, Italy

**Keywords:** Contrast media, Contrast-enhanced ultrasound, Drug-related side effects and adverse reactions, Safety, Microbubbles

## Abstract

**Objectives:**

This systematic review and meta-analysis are aimed at evaluating the incidence of adverse drug reactions (ADRs) following administration of clinically approved ultrasound contrast agents (UCAs) in adults and children, to assess risks in patients with cardiovascular disease and in pregnancy, and to evaluate the effectiveness of emergency management of severe ADRs.

**Materials and methods:**

A PRISMA 2020 systematic review was conducted searching PubMed, Scopus, and Embase. Two reviewers independently screened, extracted data, and assessed quality. Incidence estimates were pooled when feasible, stratified by age group, contrast agent, and administration route.

**Results:**

Seventy-four studies encompassing > 1 million adults and > 36,000 children were included, contributing multiple analytic cohorts to the quantitative synthesis. Severe acute ADRs were extremely rare (6 and 16 cases per 100,000 in adults and children, respectively) and absent following endocavitary administration in children. Non-severe acute ADRs occurred in 11 and 8 cases per 10,000 adults and children, respectively. Delayed reactions were very rare (< 1 case per million in adults). No significant safety differences emerged between UCA products. The incidence of ADRs in patients with cardiovascular disease was analogous to the general population. No ADRs were reported in pregnant women. Standard emergency management was effective in almost all serious cases, though rare fatalities occurred.

**Conclusion:**

UCAs show an excellent safety profile in adults and children, with very rare severe ADRs and few non-severe, typically self-limiting reactions. Strict adherence to recommended emergency management protocols mitigates the remaining risks, supporting safe use across a broad range of clinical indications.

**PROSPERO registration:**

CRD42023432668.

**Key Points:**

***Question*** *What is the incidence, type, and severity of acute and delayed ADRs associated with clinically approved UCAs across different patient populations*?

***Findings*** *Severe acute adverse reactions are very rare, and non-severe reactions are rare and self-limiting, with no significant safety differences between adults, children, or patients with cardiovascular disease*.

***Clinical relevant*** *UCAs show an excellent safety profile across populations. These findings support their safe clinical use as reliable alternatives to iodine-based and gadolinium-based contrast agents in routine diagnostic imaging*.

**Graphical Abstract:**

**Figure:**
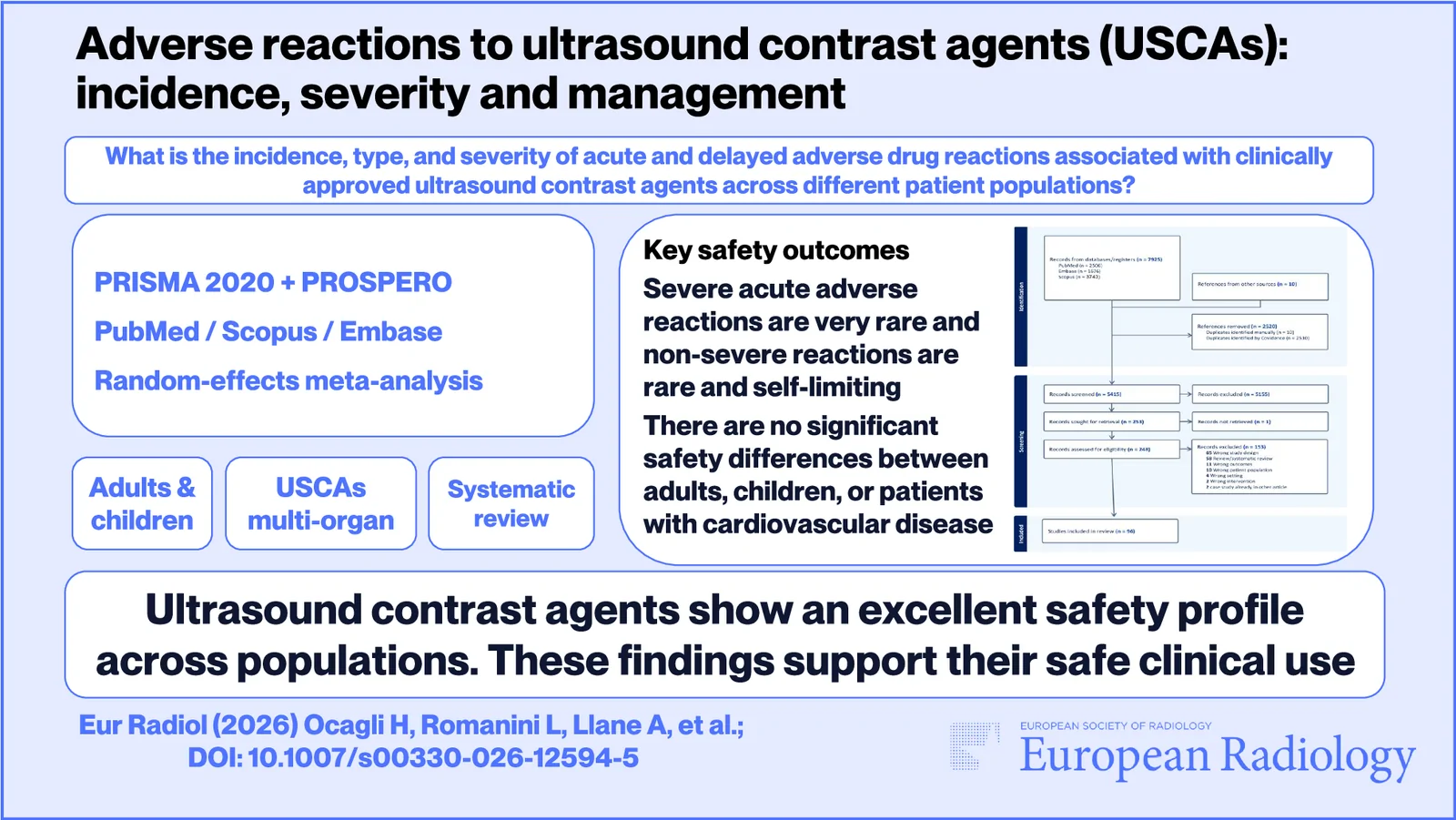

## Background

A variety of ultrasound contrast agents (UCAs) are used in clinical practice for intravascular and endocavitary applications (Table 1) [1, 2]. Their established indications are comprehensively described in international guidelines [3–5]. It is widely recognised that UCAs have an excellent safety profile [6–11] and that even severe renal impairment does not represent a significant contraindication to their use [12]. However, as with all contrast agents, adverse events have occasionally been reported. Adverse events are defined by all health authorities as any untoward medical occurrence associated with the use of a medicinal product used to treat or diagnose disease in humans, whether or not considered product related, while adverse drug reactions (ADRs) are defined as those adverse events for which a causal relationship between exposure to a medicinal product and an untoward medical occurrence is at least a reasonable possibility [13]. Most ADRs are self-limiting, resolving rapidly without sequelae, although life-threatening reactions and even fatalities have been very rarely reported [6–10].

**Table 1:** UCAs available on the market

UCAs gas core	Brand name	Shell composition	Year of approval	Population approved	Application
Perfluoropropane (Perflutren, C₃F₈)	Optison® (GE Healthcare)	Human serum albumin	1997 (USA, FDA)	Adults + paediatrics [41]	Mainly cardiology (echocardiography) in the USA^1^
Perfluoropropane (Perflutren, C₃F₈)	Definity® (Lantheus)	Phospholipid	2001 (USA, FDA)	Adults + paediatrics [42]	Mainly cardiology (echocardiography, LV opacification)^2^
Sulfur hexafluoride (SF₆)	SonoVue®/ Lumason® (Bracco)	Phospholipid	2001 (Europe, EMA); 2014 (USA, FDA)	Adults + paediatrics (not recommended in pregnancy)	Cardiology and abdominal imaging (liver, vesicoureteral reflux in paediatrics)^3^
Perflubutane (C₄F₁₀)	Sonazoid® (GE/Daiichi Sankyo)	Phospholipid	2007 (Japan, PMDA); later in Korea, China, Norway (2014), not FDA approved	Adults only (no paediatric/obstetric approval)	Abdominal imaging (especially liver, Kupffer phase, lymph nodes)^4^

^1^ FDA Prescribing Information (Optison): https://www.accessdata.fda.gov/drugsatf da_docs/label/2021/020899s024lbl.pdf

^2^ FDA Prescribing Information (Definity): https://www.accessdata.fda.gov/drugsatfda_docs/nda/2001/21-064_Definity_prntlbl.pdf

^3^ FDA Prescribing Information (Lumason): https://www.accessdata.fda.gov/drugsatfda_docs/nda/2014/203684Orig1s000Lbl.pdf; EMA Summary of Product Characteristics (SonoVue): https://www.ema.europa.eu/en/documents/product-information/sonovue-epar-product-information_en.pdf

^4^ GE Healthcare Sonazoid product information (Norway): https://www.gehealthcare.com/about/newsroom/press-releases/ge-healthcare-announces-norwegian-medicines-agency-approval-sonazoid%e2%84%a2-powder-and

PMDA (Japan) approval summary (Japanese): https://www.pmda.go.jp/PmdaSearch/iyakuDetail/ResultDataSetPDF/780052_21800AMY10105000_A_01_01

The available safety data are deemed sufficient to support the use of UCAs in clinical practice [5]. However, their exact safety profile remains incompletely characterised. The current evidence is limited by the lack of systematic reviews and meta-analyses, the predominance of retrospective studies, and the frequent pooling of different agents, patient populations, and administration routes. Another limitation is the inconsistent distinction between confounded and true hypersensitivity reactions, which makes causality assessment uncertain. Data on the management of life-threatening adverse reactions are also scarce. Indeed, in radiological publications, the terms of adverse events and adverse reactions are often used interchangeably, making it difficult to distinguish between reactions directly caused by drug administration and unrelated events. This issue is particularly relevant in the cardiology literature, where adverse events may result from the administration of other drugs or from exercise.

Consequently, the ESUR Contrast Media Safety Committee (ESUR-CMSC) deemed it necessary to conduct a systematic review of the available literature on ADRs associated with clinically approved microbubble contrast agents, to clarify their safety profile, incidence, and the characteristics of unconfounded adverse reactions.

The primary objective is to assess the incidence of acute and delayed ADRs following microbubble administration in both adults and children. Specific aims are to determine the incidence of acute ADRs stratified by severity; evaluate the incidence of delayed ADRs; compare ADRs across different microbubbles; assess risks in patients with cardiovascular disease or pregnant women; and evaluate whether standard treatments used for contrast agent–related ADRs are effective for microbubble-associated reactions.

## Methods

### Protocol and registration

This systematic review was conducted in accordance with the Preferred Reporting Items for Systematic Reviews and Meta-Analyses (PRISMA) 2020 guidelines [14] and was registered in PROSPERO (protocol no. CRD42023432668).

According to the ESUR Contrast Media guidelines, an ADR was classified as acute if it occurred within one hour of contrast agent injection and delayed if it occurred between one hour and one week after contrast injection [15]. Acute reactions have been categorised as hypersensitivity or non-hypersensitivity reactions [16]. In the radiological literature, they are further graded as mild, moderate, and severe based on the severity of the symptoms (Tables S1 and S2) [15]. In this work, mild and moderate reactions are grouped in “non-severe” ADRs, as the literature is often inconsistent in their description and classification. This corresponds to the division used in the farmacovigilance records, where ADRs are classified as serious and non-serious. An ADR was considered serious if it resulted in death, was life-threatening, required hospitalisation or prolonged existing hospitalisation, led to persistent or significant disability or incapacity, or was associated with a congenital anomaly or birth defect [17].

The frequency of ADRs was defined according to the European Medicines Agency guidelines as uncommon (≥ 1/1000 to < 1/100), rare (≥ 1/10,000 to < 1/1000) and very rare (< 1/10,000) [18].

For paediatric populations, we distinguished between intravenous administration during contrast-enhanced ultrasound (CEUS) and endocavitary administration for vesicoureteral reflux assessment (ceVUs). Management strategies, including pharmacological treatments and supportive measures, were extracted. For pregnant women, only studies and case reports specifying the microbubble used, and both maternal and foetal outcomes were included. As most studies lacked detailed or consistent classifications of cardiovascular comorbidities, we adopted the overarching category “cardiovascular disease” to facilitate comparability across studies. In this context, the term ‘cardiovascular disease’ is used in a broad sense, encompassing a wide range of cardiac and vascular conditions.

#### Population-intervention-comparison-outcome (PICO) Questions formulation

Research questions were formulated according to the PICO framework (Table 2). PICO1 focuses on the general adult and paediatric populations, aiming to understand the incidence, type, and severity of both acute and delayed ADRs following microbubble contrast administration. PICO2 focuses on patients with known cardiovascular conditions, comparing their risk of ADRs with that of the general population. PICO3 concerns patients who have experienced severe ADRs from microbubble contrast agents, comparing their treatment outcomes with those following similar reactions to iodine- or gadolinium-based agents. PICO4 investigates the risk profile for pregnant women undergoing CEUS, comparing their outcomes with those of the general adult population.

**Table 2:** PICO framework was used in this systematic review to answer clinically relevant questions related to the safety of microbubble-based contrast agents

PICO		Questions	Population	Intervention	Comparison	Outcome
PICO 1	Incidence and characteristics of acute and delayed adverse reactions in adults and children undergoing CEUS with different microbubble contrast agents	1. What is the incidence of acute adverse reactions of different grades in adults and children undergoing CEUS? 2. What is the incidence of delayed adverse reactions in adults and children undergoing CEUS? 3. Do acute and delayed adverse reactions differ among the various microbubble contrast agents? 4. Is the incidence of adverse reactions different in adults and children?	Adults and children, general population	Intravenous, intraarterial, endocavitary microbubble contrast agent administration	Not applicable	Incidence, type, and severity of acute adverse reactions; Incidence, type, and severity of delayed adverse reactions
PICO 2	Risk of adverse events in cardiovascular populations after microbubble contrast agent administration	5. Are patients with cardiovascular diseases at increased risk of acute reactions compared to the general population after microbubble contrast agent administration?	Patients with known cardiovascular diseases	Intravenous, Intraarterial, endocavitary microbubble contrast agent administration	Adults and children, general population	Incidence, type, and severity of acute adverse reactions; Incidence, type, and severity of delayed adverse reactions
PICO 3	Effectiveness of standard treatments for serious adverse reactions following microbubble vs Iodine- or gadolinium-based contrast agents	6. Is the standard treatment used to manage serious adverse reactions after iodine-based and gadolinium-based contrast agent administration also effective in patients who develop acute and/or delayed reactions after microbubble contrast agent administration, and what are the associated outcomes?	Patients with serious ADRs after microbubble contrast agent administration	Standard medication	Outcome of similar adverse reactions after iodine-based and gadolinium-based contrast agents	Recovery/death
PICO 4	Risk profile of microbubble contrast agent administration in pregnant women compared to the general adult population	7. What is the risk profile of microbubble contrast agent administration in pregnant women compared to the general adult population?	Pregnant women	Intravenous, Intraarterial, endocavitary microbubble contrast agent administration	Adults, general population	Incidence, type, and severity of acute adverse reactions; Incidence, type, and severity of delayed adverse reactions

#### Eligibility criteria

We included controlled and observational studies involving adults or children who received UCAs and reported ADRs. Eligible designs comprised cohort and case-control studies for harm assessment, as well as case reports describing severe ADRs and their treatment, which were used to evaluate pharmacological management and outcomes (PICO3). Case reports were also considered to assess the frequency and type of ADRs in pregnant women (PICO4). Animal studies and investigations on UCAs withdrawn from the market, not yet approved, or used solely in experimental settings, were excluded.

#### Information sources and search strategy

A comprehensive search was conducted in PubMed, Scopus, and Embase, with the last update in May 2025. No date or language restrictions were applied in the search strategy. The search strategy combined free-text terms and controlled vocabulary related to microbubble contrast agents, patient characteristics, administration routes, and ADRs. Full search strings are provided in the Supplementary Materials (Table S3).

#### Study selection

In accordance with PRISMA 2020 terminology, the term “records” refers to database search results prior to eligibility assessment, whereas “studies” refer to unique publications meeting the inclusion criteria. For quantitative synthesis, some studies contributed more than one analytic cohort, which were treated as distinct analytic units when results were reported separately by population, contrast agent, or administration route.

Citations were uploaded to Covidence review software for screening (Veritas Health Innovation, Melbourne, Australia, available at www.covidence.org). Two independent reviewers screened titles and abstracts, followed by full-text screening to confirm eligibility. At the full-text stage, studies were most commonly excluded because they did not report ADRs related to UCAs, involved contrast agents not approved or withdrawn from clinical use, focused on experimental or animal models, included overlapping populations, or provided insufficient data for quantitative extraction.

Disagreements were resolved through discussion, with a third reviewer available if needed.

#### Data extraction

Two reviewers independently extracted data on study characteristics, contrast agent type, administration route, examination type, patient demographics, and all reported acute or delayed safety events. Studies by the same authors were screened for overlap. Each reported event was then independently assessed for causality by two authors (M.B. and L.R.) and adjudicated by consensus to determine whether it met the definition of an adverse drug reaction (ADR). Adverse events without a reasonable causal relationship to microbubble contrast administration were excluded. Therefore, this systematic review and meta-analysis address ADRs only.

#### Quality assessment

The quality assessment of the included studies was evaluated using the tools provided by the Joanna Briggs Institute (JBI), tailoring the selection of tools according to the specific study designs. Given the diversity of study types in our review, we applied the JBI critical appraisal checklist for prevalence studies [19], case series [20], case reports [20], cohort studies [20], and randomised controlled trials (RCTs) [21] as appropriate. Two reviewers (M.B. and L.R.) performed the assessment, and disagreements were resolved through consensus.

#### Data synthesis

Where possible, data were pooled for meta-analysis. For studies not amenable to pooling, results were synthesised narratively, grouped by population, UCA type, and administration route.

#### Statistical analysis

Analyses were conducted at the subgroup level, defined by study-reported characteristics. Consequently, a single study could contribute multiple times when adverse events were reported separately for different subgroups; in those cases, we extracted subgroup-specific events and denominators and treated them as distinct study arms [22].

Pooled incidence estimates of ADRs were calculated using random-effects models for proportions. For rare outcomes, we applied logit transformation and generalised linear mixed models (GLMM) as recommended for sparse data [21]. Random-intercept models were fitted to account for between-study heterogeneity, allowing each study-specific proportion to vary around an overall mean effect. This modelling strategy permits inclusion of studies with zero events without the need for continuity corrections, which may bias estimates in rare-event settings. Heterogeneity was quantified with the *I*² statistic [23], with values greater than 50% indicating substantial heterogeneity. Subgroup analyses and meta-regression were performed to explore sources of heterogeneity, including age group (adult vs paediatric), contrast agent type, and administration route (intravenous vs endocavitary).

For outcomes characterised by extreme sparsity or the complete absence of events across studies, a two-part Bayesian approach was implemented [24]. First, the probability that at least one adverse event occurred across the included studies was estimated. Second, conditional on event occurrence, the incidence proportion was modelled using a binomial likelihood with weakly informative Beta priors. Specifically, Beta (0.5, 0.5) or Beta (0.1, 1) priors were used to stabilise estimates and avoid degenerate posterior distributions when no events were observed. Posterior estimates are reported with 95% credible intervals.

Analyses were performed with R software (version 4.4.2) [25], primarily using the *metafor* package for frequentist models and base Bayesian updating for sparse-event analyses. The code used for the analyses is available at: https://github.com/UBESP-DCTV/uscas-adr-ma.

## Results

**Fig. 1:**
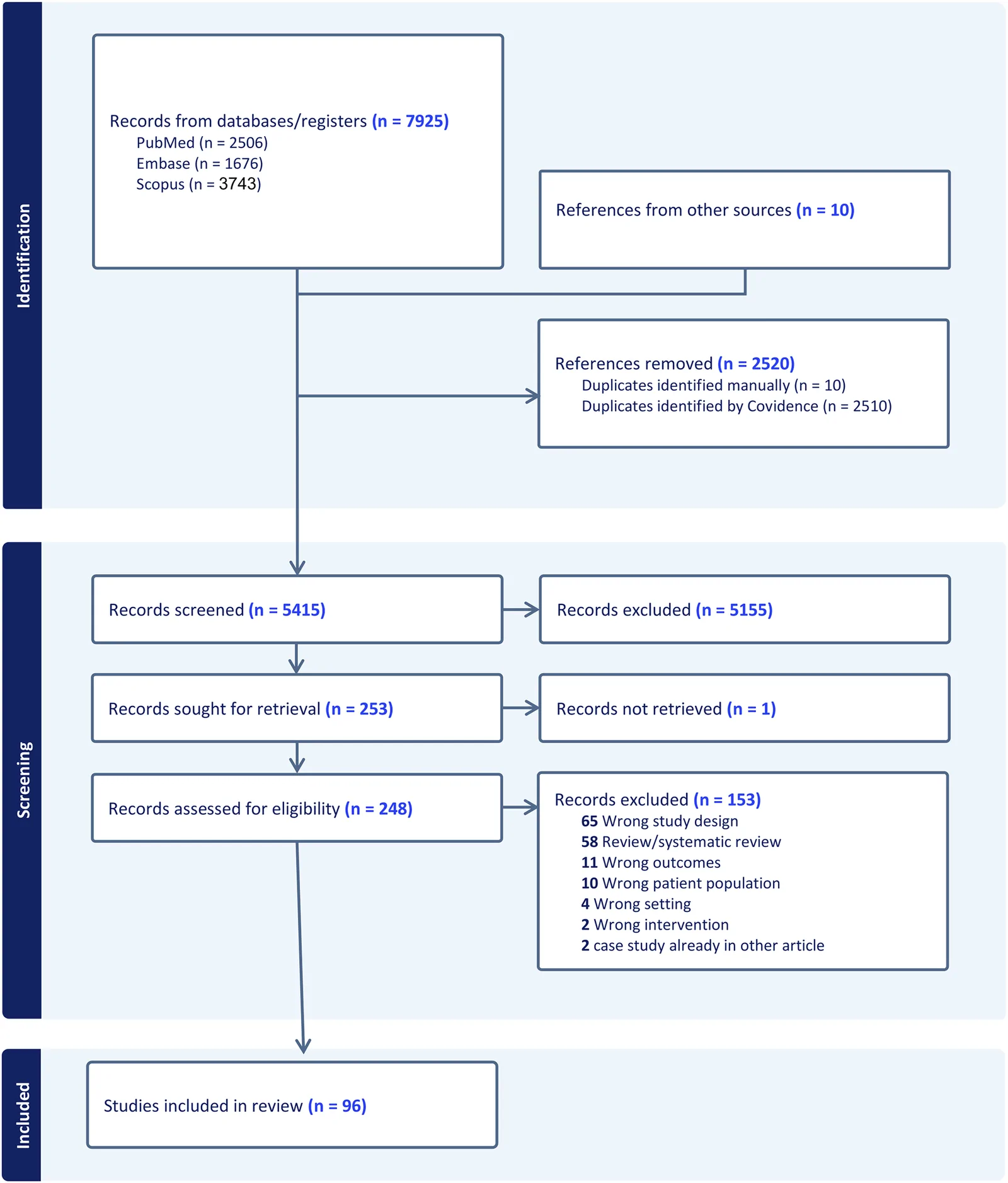
PRISMA Flowchart of the study selection process

The systematic search identified 7925 records, of which 5415 remained after deduplication for title and abstract screening. 248 full-text studies were assessed for eligibility, and 96 met the inclusion criteria for data extraction. Of these, 74 studies were considered in the analysis of ADR prevalence, while 22 studies consisted of case reports describing severe ADRs and their management. The latter were included to evaluate pharmacological management and outcomes (PICO3) or to provide safety data on the use of UCAs during pregnancy (PICO4). The PRISMA flow diagram is presented in Fig. 1.

### Study characteristics

Among the 74 studies included in the quantitative synthesis, a total of 90 analytic cohorts were identified (Table 3). These cohorts were predominantly derived from prospective (51%) or retrospective observational cohorts (23%), with 35 multicentre studies (39%), randomised controlled trials, case series, and case reports (Table 3). Study periods ranged from the early 2000s to recent years, and patient ages spanned both adult and paediatric populations. Across all included studies, the review synthesised evidence from more than 1 million adults and over 36,000 paediatric patients. The type of microbubble contrast agent used was SonoVue® (56%), Optison® (15%), Definity® (21%), and Sonazoid® (4.5%). Some studies explicitly addressed potential confounding factors, while others reported events without clarifying causal relationships (Table S4).

**Table 3:** Summaries of the characteristics of the included studies. The table does not report the studies on treatment

Characteristic		*N* = 90^1^
Type of examinations	Abdominal CEUS	21 (23.3%)
	Breast CEUS	2 (2.2%)
	CeVUS	16 (17.8%)
	Chest ultrasonography	2 (2.2%)
	Echocardiography	2 (2.2%)
	Musculoskeletal CEUS or myocardial CEUS	2 (2.2%)
	Rest and stress echocardiography	12 (13.3%)
	Rest echocardiography	12 (13.3%)
	Stress echocardiography	11 (12.2%)
	Multiple anatomical sites	10 (11.1%)
Contrast agent	SonoVue®/Lumason®	51 (56.7%)
	Definity®	19 (21.1%)
	Optison®	13 (14.4%)
	Optison® or Definity®	3 (3.3%)
	Sonazoid®	4 (4.4%)
Multicenter	Yes	35 (39%)
	No	55 (61%)
Study design grouped	Case report/case series	5 (5.6%)
	Experimental studies	13 (14.4%)
	Observational prospective	46 (51.1%)
	Observational retrospective	21 (23.3%)
	Prevalence	5 (5.6%)
Population	Adults	53 (59%)
	Adults (healthy volunteers)	4 (4.4%)
	Children	26 (29%)
	Children and adults	3 (3.3%)
	Pregnant women	4 (4.4%)
Cardiovascular disease	Yes	42 (47.2%)
	No	48 (52.8%)
Route of administration	Endocavitary	17 (19%)
	Intravenous	73 (81%)

^1^ Absolute number (percentage), *n* (%)

### PICO1—severe acute ADRs

Table 4 and Figs. S1–S14 report all the estimates and 95% confidence intervals. Across 57 adult cohorts, including 831,262 CEUS examinations, the pooled incidence of severe acute ADRs was estimated at ~6 per 100,000. In children, in 29 prospective and retrospective studies including 36,212 patients, severe acute ADRs were also rare, with an incidence of 16 per 100,000. There were no cases of severe acute ADRs following endocavitary administration in children, with a Bayesian estimated incidence of ~1 per 100,000.

**Table 4:** Pooled incidence estimates of acute (serious and non-serious) and delayed ADRs by population subgroup (adults, children) and administration route (endocavitary, intravenous) in general, cardiologic, and non-cardiologic populations

Outcome	Population	*N* patients	*N* events	Pooled incidence as percentage (95% CI)	Events/patients
Serious acute	Adults	831,152	47	0.006% (0.002%, 0.018%)	1/17,000
	Children intravenous	1813	3	0.01646% (0.00124%, 0.21775%)	1/604
	Children endocavitary	7872	0	0.0013% (0%, 0.0124%)^*^	1/76,923
Non-serious acute	Adults	764,978	459	0.1094% (0.0384%, 0.3120%)	1/1666
	Children intravenous	2203	30	0.081% (0.011%, 0.590%)	1/1234
	Children endocavitary	56	1	0.000970% (0.0000000669%, 14.077%)	1/56
Delayed	Adults	756,091	13	0.0000814% (0.000000638%,0.01036%)	1/58,161
	Children intravenous	2588	0	0.0193 (0%, 0.097%)	1/5181
	Children endocavitary	531	1	0.02% (0%, 2.590%)	1/5000
Serious acute	Adults	175,550	13	0.002% (0%, 0.017%)	1/50,000
	Children	133	0	0.0746% (0%, 0.7296%)^*^	1/1340
Non-serious acute	Adults	0	0	-	-
	Children	0	0	-	-
Delayed	Adults	655,712	10	0.0003% (0.000000%, 0.038%)	1/65,571
	Children	133	0	0.075% (0%, 0.73%)^*^	1/1340
Serious acute	Adults	655,712	34	0.006 (0.004%, 0.011%)	1/16,666
	Children	2491	3	0.120 (0.039%, 0.373%)	1/833
Non-serious acute	Adults	655,712	268	0.217 (0.048%-0.975%)	1/460
	Children	2491	26	1.229% (0.512%, 2.921%)	1/82
Delayed	Adults	655,712	10	0, 0.00031%	1/3257
	Children	2459	2	0.0041% (0, 0.0398%)	1/24,390

^*^ For 2 part analysis, the credible intervals are reported

### PICO1—non-severe acute ADRs

Across 55 observational cohorts, in 764,978 adults undergoing CEUS, the incidence of non-severe acute ADRs was estimated at ~11 cases per 10,000.

Among 29 studies in children undergoing CEUS, the incidence of non-severe acute ADRs was ~8 cases per 10,000. In the 16 cohorts on endocavitary use in children, the pooled incidence of non-severe adverse reactions was estimated at ~1 case per 100,000.

### PICO1—delayed ADRs

In 756,091 adults undergoing CEUS, pooled delayed adverse reactions had an incidence of ~1 case per million. Among children receiving microbubbles intravenously, delayed adverse reactions were not reported, whereas in endocavitary use, the estimated incidence was ~2 cases per 100,000.

### Meta-regression

In adults, compared with experimental studies, prospective observational studies showed lower odds of severe acute ADRs (OR 0.05, 95% CI: 0.01–0.21) and non-severe acute ADRs (OR 0.03, 95% CI: 0.01–0.12). Retrospective observational studies showed even lower odds for severe 0.025 (95% CI: 0.0053–0.1165) and non-severe 0.020 (95% CI: 0.0043–0.0916) acute ADRs. For delayed reactions in adults, both prospective and retrospective designs were associated with reduced odds; the prevalence design level was also associated with lower odds, 0.071 (95% CI: 0.0058–0.8694). No moderator achieved significance in paediatric IV analyses (Table S5). In paediatric endocavitary analyses, delayed ADRs showed the same significant design pattern (prospective 0.037, 95% CI 0.01–0.16; retrospective 0.013, 95% CI: 0.002–0.07; prevalence 0.071, 95% CI: 0.006–0.87).

### PICO2—risk ADRs stratified according to cardiologic vs non-cardiologic

In cardiologic CEUS, severe acute ADRs were very rare in adults (2 cases per 100,000) and were not observed in children, with a model-based estimate of 7 per 100,000. Non-severe acute ADRs were not reported in either age group for cardiologic CEUS.

In non-cardiologic patients, severe acute ADRs were very rare in adults (6 per 100,000). Three were described in children. Non-severe acute ADRs were rare in adults (22 cases per 10,000) and uncommon in children (~1%). Non-cardiologic pooled delayed ADRs proportion was 3 and 4 cases per 100,000 in adults and children, respectively.

### PICO2—question 3 subgroup analysis according to contrast agent

Across contrast agents (Table 5), acute severe reactions were very rare for SonoVue® and Definity® (11 and 9 cases per 100,000, respectively), with zero events for Optison® (estimated 3 cases per 100,000). Acute non-severe reactions were higher for SonoVue® (21 cases per 10,000), lower for Definity® (9 cases per 10,000) and Optison® (2 cases per 10,000). Delayed reactions were very rare, with only four events described for SonoVue® and one for Definity®, respectively. For Sonazoid®, evidence was too sparse for meta-analysis: across four small studies (438 patients), no acute severe or non-severe reactions were observed, and four delayed events were reported in a single breast-CEUS trial [26]. Accordingly, any conclusion regarding the safety of Sonazoid® should be interpreted with caution, as it are limited by the small number of included studies and patients.

**Table 5:** Pooled incidence estimates of acute (serious, non-serious) and delayed adverse reactions by contrast agent (SonoVue®, Definity®, Optison®)

Outcome	*N* patients	Contrast agent	*N* events	Pooled incidence as percentage (95% CI)	Events/patients
Serious acute	669,712	SonoVue®	8	0.011% (0.0036%, 0.032%)	1/9090
	103,897	Definity®	3	0.0087% (0.0045%, 0.0166%)	1/11,494
	29,674	Optison®	0	0.0003% (0%, 0.0033%)	1/333,333
Non-serious acute	669,712	SonoVue®	23	0.218% (0.082%, 0.58%)	1/460
	37,733	Definity®	9	0.0087% (0.0045%, 0.0166%)	1/11,544
	29,554	Optison®	4	0.0179% (0.000242%, 1.3158%)	1/5573
Delayed	669,712	SonoVue®	4	0.00054% (0.000026%, 0.0113%)	1/167,428
	31,718	Definity®	1	0.113% (IC 95% 0.021%, 0.604%)	1/886
	29,564	Optison®	0	0.0003% (0, 0.0033%)^*^	1/333,333

^*^ For 2 part analysis, the credible intervals are reported

### PICO3—effectiveness of standard treatments for severe ADRs

The dataset includes 49 cases of severe ADRs following intravenous administration of UCAs, comprising both case reports and severe ADRs from larger observational studies in which the medical management was described (Table S6). Severe ADRs were grouped into three main clinical phenotypes: (i) anaphylaxis/anaphylactoid presentations; (ii) cardiac arrest (*n* = 12, including two fatal outcomes) requiring advanced cardiopulmonary resuscitation manoeuvres; and (iii) allergic acute coronary syndrome (Kounis syndrome).

Most severe ADRs were consistent with immediate-type reactions, ranging from severe anaphylaxis with dyspnoea, bronchospasm, hypotension, bradycardia and loss of consciousness to rapidly progressive systemic manifestations culminating in cardiac arrest. Symptom onset was most often immediate (24%) or within a few minutes (46%) after contrast administration, consistent with immediate hypersensitivity or non-IgE-mediated mechanisms. Management frequently required advanced interventions, including airway support and intubation, cardiopulmonary resuscitation, epinephrine infusion, and vasopressors.

Four definite cases consistent with Kounis syndrome were identified following SonoVue®/Lumason® administration [27–30]. One additional case was considered probable based on the reported clinical presentation [31]. These cases highlight that, although rare, coronary involvement may occur in the context of severe contrast-associated reaction. Cardiac arrest occurred in 12 patients, with two fatalities likely related to contrast agent administration; however, in one case, no autopsy was performed [32], and in the other, pathological examination found no evidence of an acute allergic reaction [33].

Allergy testing was inconsistently performed and variably reported. Skin prick tests and intradermal tests were performed with SonoVue® in four cases and were negative at any time [34–37]. In 3 cases, the tests were negative for one of the excipients (macrogol/Polyethene glycol (9) [34, 37, 38]; in one case, the basophil activation test was positive for SonoVue® and excipients like macrogol/PEG 2000, but negative for macrogol/PEG 2000 alone [39], in another case, it was negative for SonoVue® alone [36]. When evaluated, the levels of Tryptase and Histamine were elevated [31, 34, 37, 39, 40], only in one case were they normal.

### PICO4—pregnant women

No severe ADRs were documented following UCA administration during pregnancy, but evidence was limited to small registry datasets and case series. In total, 17 pregnant patients underwent CEUS examinations, with 24 administrations performed (Table S7). Non-severe acute reactions were not reported, and no maternal or foetal complications were reported during the follow-up. Accordingly, any conclusion regarding the safety of UCAs during pregnancy should be interpreted with caution, given the very small number of reported patients.

### Risk of bias

Overall, the methodological quality was mixed across designs (Tables S8–S14). Case reports were generally well described but often lacked details on adverse events or follow-up. Case series were acceptable, though completeness of inclusion and statistical analyses were inconsistent. RCTs showed variable quality, with frequent shortcomings in allocation concealment and blinding, especially in older trials. Cohort and cross-sectional studies commonly fail to control for confounding or ensure full follow-up. In contrast, prevalence and most diagnostic accuracy studies were generally robust, with appropriate sampling and measurement methods.

### Heterogeneity across pooled estimates

Substantial heterogeneity was observed across several pooled estimates. In particular, analyses of non-severe acute ADRs in adults showed high between-study variability, with *I*² values exceeding 75% in most models. Moderate to substantial heterogeneity was also detected for non-severe ADRs in children following intravenous administration and for delayed ADRs in adults, whereas heterogeneity was generally lower for severe ADRs, especially in paediatric and endocavitary settings.

Meta-regression analyses identified study design as a significant contributor to heterogeneity. Compared with experimental studies, both prospective and retrospective observational studies were associated with significantly lower reported incidences of severe and non-severe ADRs in adults, as well as delayed ADRs. No consistent association was observed for the presence of reported confounding factors. Detailed results of the meta-regression analyses are reported in Supplementary Table S5, and forest plots illustrating between-study variability are provided in Figures S1–S14.

## Discussion

This systematic review synthesises the largest body of evidence to date on the safety profile of UCAs in adult, paediatric, and special patient populations, including pregnant women and those with cardiovascular disease.

Our findings confirm that UCAs are associated with a very low incidence of acute severe reactions in adults and negligible delayed reactions (~0.0001%), with paediatric estimates more uncertain due to small denominators. Non-severe reactions are more frequent than severe ones but remain uncommon overall. A recent global pharmacovigilance analysis of Lumason®/SonoVue® reported an overall ADR risk of 0.024% and a serious-event risk of 0.0096%, with serious hypersensitivity reactions estimated at < 1 in 10,000 administrations, reinforcing the rarity we observed [41].

Emergency management is generally effective, but rare intensive care unit admissions and isolated fatalities are reported, underscoring the need for preparedness.

The available evidence on UCA use in pregnancy is very limited and remains insufficient for definitive safety conclusions. In our review, only four case reports were identified, all showing no maternal or foetal adverse events. The recent scoping review by Dassen et al [42], which applied broader inclusion criteria and included 13 studies with 256 women and multiple agents, likewise did not identify clinically relevant maternal, foetal, pregnancy, or neonatal outcomes related to CEUS. While these observations are reassuring, they are based on small, heterogeneous datasets, and the absence of large-scale, controlled studies warrants caution. Further research is needed before issuing robust recommendations.

Agent-specific analyses are directionally consistent with the stratified results: point estimates for acute severe reactions with SonoVue® (0.011%) and Definity® (0.0087%) are of the same order of magnitude, while Optison® and Sonazoid® show near-zero estimates but with very sparse data. For delayed reactions, only a few reactions have been reported, confidence intervals are wide, and no firm between-agent difference is supported (Table 5).

A recent real-world, retrospective propensity-score–matched cohort study of 11.4 million insured US patients (2018–2022) analysed nationwide claims to assess the rate of immediate severe adverse events after UCA-enhanced vs unenhanced echocardiography [43]. Overall, 4.4% underwent contrast-enhanced procedures. After matching, mortality was lower in UCA recipients, with no differences across agents. Non-fatal serious adverse events were comparable between enhanced and unenhanced studies. These findings support that currently used UCAs are associated with very low rates of severe adverse reactions despite pharmacological differences; however, evidence for Sonazoid® remains limited, and data are insufficient for firm cross-agent comparisons.

The strengths of this review include a comprehensive search strategy, a large, aggregated sample size, and the inclusion of both adult and paediatric populations, which enhances the generalisability of the findings.

Limitations mainly relate to the predominance of observational study designs, variability in event definitions and reporting, and limited representation of specific high-risk groups such as pregnant women and paediatric patients with cardiovascular disease, favouring under-reporting and between-study variability (*I*² often high). The high heterogeneity observed in several pooled estimates likely reflects the marked clinical and methodological diversity of the included studies rather than random variation alone. Differences in UCAs, administration route (intravenous vs endocavitary), and target population contributed to variability in reported ADR rates. In addition, study design emerged as a key determinant: observational studies, particularly large pharmacovigilance-based cohorts, consistently reported lower ADR incidences than experimental or early-phase studies, suggesting potential differences in monitoring intensity, outcome definitions, and reporting thresholds. Further heterogeneity may derive from inconsistent terminology and classification of ADRS vs adverse events across studies, especially for non-severe reactions, which are more prone to subjective reporting and under-ascertainment.

Another limitation is the extreme rarity of severe reactions: while reassuring from a clinical perspective, this limits the statistical power to detect subtle differences between agents.

The rarity of severe reactions, although clinically reassuring, reduces statistical power to detect subtle differences between agents. Rare-event meta-analysis is fragile, as many studies include zero or very few events, leading to model-dependent estimates, wide confidence intervals, and low power for moderator effects. Small subgroups yield imprecise estimates. Additional limitations include possible confounding from co-interventions, unblinded adjudication, residual duplication, publication bias, and regional differences in formulations, dosing, and practice that may limit generalisability.

In conclusion, UCAs show an excellent overall safety profile in both adult and paediatric populations, with very few severe ADRs and a low rate of non-severe reactions. There is no evidence of clinically meaningful safety differences between agents in the available literature. Strict adherence to recommended emergency management protocols effectively mitigates the remaining risks, supporting the safe use of CEUS across a broad spectrum of clinical indications. However, evidence in specific subgroups, particularly pregnant women and paediatric patients with cardiovascular disease, remains very limited and does not allow definitive safety conclusions. In addition, the evidence base for Sonazoid is currently small, and available data are insufficient for meaningful safety comparisons with other agents. High-quality studies are needed to refine risk estimates in selected patient groups and to optimise management strategies across care settings.

## Supplementary information

ELECTRONIC SUPPLEMENTARY MATERIAL

## Abbreviations

ADRs: Adverse drug reactions
ceVUs: endocavitary administration for vesicoureteral reflux assessment
CEUS: Contrast-enhanced ultrasound
GLMM: Generalised linear mixed models
UCAs: ultrasound contrast agents
