# Supplementary material for: Type, severity, frequency and management of adverse reactions associated with ultrasound contrast agents: a systematic review and meta-analysis
Source: Eur Radiol. 2026 May 15;36(10):7671–83. doi: 10.1007/s00330-026-12594-5 (PMC13574902; doi:10.1007/s00330-026-12594-5)
Supplement: Supplementary file 1 [file 330_2026_12594_MOESM1_ESM.pdf]

Type, Severity, Frequency and Management of Adverse Reactions Associated with Ultrasound Contrast Agents: A Systematic Review and Meta-Analysis

ELECTRONIC SUPPLEMENTARY MATERIAL

Eur Radiol (2026) Ocagli H, Romanini L, Llane A, et al.

Sommario

Tables 4
Table S1 - Symptoms of hypersensitivity reactions to contrast agents and their grading according to the ACR 4
Table S2 - Symptoms of non-hypersensitivity adverse reactions to contrast agents and their grading according to the ACR 5
Table S3 - Search strategies for each consulted source 6
Table S4 - Study Characteristics of the included studies 10
Table S5 - Meta-regression results 15
Table S6 - Case reports reporting treatment of serious adverse events 17
Table S7 - Characteristics of studies on pregnant women 31
Table S8 - JBI Critical Appraisal - Case Report 33
Table S9 - JBI Critical Appraisal - Case Series.................................36
Table S10 - JBI Critical Appraisal - Randomized Controlled Trial...38
Table S11 - JBI Critical Appraisal - Prevalence Study 40
Table S12 - JBI Critical Appraisal - Cohort Study.............................41
Table S13 - JBI Critical Appraisal - Diagnostic Test Accuracy Study 44
Table S14 - JBI Critical Appraisal - Cross Sectional Study 45
Figures..46
Figure S1 - Forest Plot Severe Adverse Events – Adults.................46
Figure S2 - Forest Plot Severe Adverse Events - Children IV..........47
Figure S3 - Forest Plot Non-Severe Adverse Events – Adults 48
Figure S4 - Forest Plot Non Severe Adverse Events – Children IV .49
Figure S5 - Forest Plot Non Severe Adverse Events – Children Endocavitary 50
Figure S6 - Forest Plot Delayed Adverse Events – Adults...............51
Figure S7 - Forest Plot Delayed Adverse Events – Endocavitary ...52
Figure S8 - Forest Plot Severe Adverse Events Cardiologic – Adults 53
Figure S9 - Forest Plot Severe Adverse Events Non Cardiologic – Adults 54

Figure S10 - Forest Plot Severe Adverse Events Non Cardiologic – Children 55
Figure S11 - Forest Plot Non Severe Adverse Events Non Cardiologic – Adults 56
Figure S12 - Forest Plot Non Severe Adverse Events Non Cardiologic – Children...57
Figure S13 - Forest Plot Delayed Adverse Events Cardiologic – Adults 58
Figure S14 - Forest Plot Delayed Adverse Events Non Cardiologic – Adults 59
References 60
PICO 3...60
PICO 1, 2, 4...64

Tables

Table S1 - Symptoms of hypersensitivity reactions to contrast agents and their grading according to the ACR

Nasal congestion	Mild
Sneezing, conjuntivitis, rhinorroea	Mild
Limited/scattered urticaria and pruritus	Mild
Cutaneous oedema	Mild
Limited itchy/scratchy throat	Mild
Diffuse orticaria and pruritus	Moderate
Diffuse erythema, no hypotension	Moderate
Facial oedema, no dyspnoea	Moderate
Throat hoarseness, no dyspnoea	Moderate
Mild broncospasm, no hypoxia	Moderate
Diffuse/facial oedema, with dyspnoea	Severe
Diffuse erythema, with hypotension	Severe
Laryngeal oedema, with hypoxia	Severe
Severe broncospasm, with hypoxia	Severe
Anaphylactic shock	Severe
Cardiopulmonary arrest	Severe

ACR American College of Radiology [1,2]

1. ACR-Manual-on-Contrast-Media.pdf, (n.d.). https://edge.sitecorecloud.io/americancoldf5f-acrorgf92a-productioncb02-3650/media/ACR/Files/Clinical/Contrast-Manual/ACR-Manual-on-Contrast-Media.pdf (accessed November 7, 2025).
2. A.J. van der Molen, A.A.J.M. van de Ven, F. Vega, I.A. Dekkers, R.W.F. Geenen, M.-F. Bellin, M. Bertolotto, T.B. Brismar, O. Clément, J.-M. Correas, K. Deike, G. Heinz, A.H. Mahnken, C.A. Mallio, C.C. Quattrocchi, A. Radbruch, P. Reimer, G. Roditi, L. Romanini, C. Sebastià, F. Stacul, Hypersensitivity reactions to contrast media: Part 1. Management of immediate and non-immediate hypersensitivity reactions in adults. Updated guidelines by the ESUR Contrast Media Safety Committee, Eur. Radiol. 35 (2025) 6798–6810. https://doi.org/10.1007/s00330-025-11675-1.

Table S2 - Symptoms of non-hypersensitivity adverse reactions to contrast agents and their grading according to the ACR

Limited nausea or vomiting	Mild
Altered or metallic taste (dysgeusia)	Mild
Feeling warm/hot	Mild
Flushing	Mild
Mild hypertension	Mild
Headache	Mild
Dizziness	Mild
Paraesthesia	Mild
Transient vasovagal reaction	Mild
Protracted nausea or vomiting	Moderate
Hypertensive urgency	Moderate
Isolated chest pain	Moderate
Responsive vasovagal reaction	Moderate
Hypertensive emergency	Severe
Pulmonary oedema	Severe
Cardiac arrhythmia	Severe
Convulsion or seizure	Severe
Unresponsive vasovagal reaction	Severe

1. van der Molen, A. J., & Vega, F. (2025). Acute non-hypersensitivity adverse reactions following intravascular administration of contrast agents in adult patients. European Journal of Radiology, 112355, 10.1016/j.ejrad.2025.112355.
2. American College of Radiology (2024) ACR manual on contrast media. v2025. Available via https://www.acr.org/Clinical-Resources/Contrast-Manual. Accessed 6 October 2025

Table S3 - Search strategies for each consulted source

Source	Search strategy
PubMed	((("Contrast Media"[Mesh] OR "contrast media"[TW] OR "contrast medium"[TW] OR "contrast agent"[TW] OR "contrast agents"[TW] OR "contrast material"[TW] OR "contrast materials"[TW] OR "radiocontrast media"[TW] OR "radiocontrast medium"[TW] OR "radiocontrast agent"[TW] OR "radiocontrast agents"[TW] OR "radiopaque media"[TW] OR "radiopaque medium"[TW] OR "radio-opaque media"[TW] OR "radio-opaque medium"[TW] OR "radiopaque agent"[TW] OR "radiopaque agents"[TW] OR "radio-opaque agent"[TW] OR "radio-opaque agents"[TW] OR "ultrasound enhancing agent"[TW] OR "ultrasound enhancing agents"[TW])<br>AND<br>("Microbubbles"[Mesh] OR “Microbubble”[TW] OR "micro-bubble"[TW] OR "micro-bubbles"[TW] OR “microbubbles”[TW] OR "Gas-filled particle"[TW] OR "Gas-filled particles"[TW] OR "Colloidal Gas Aphron"[TW] OR "Colloidal Gas Aphrons"[TW])) OR "contrast agent BR1"[Supplementary Concept] OR "contrast agent BR1"[All fields] OR “SonoVue®*”[All fields] OR “Lumason®®*”[All fields] OR "FS 069"[Supplementary Concept] OR "FS 069"[All fields] OR "FS069"[All fields] OR "perfluoropropane-filled albumin microsphere solution"[All fields] OR Optison®*[All fields] OR "Definity®"[Supplementary Concept] OR “Definity®”[All fields] OR “Luminity”[All fields] OR "Sonazoid®"[Supplementary Concept] OR “Sonazoid®”[All fields] OR "NC100100"[All fields] OR "DD-723"[All fields])<br>AND<br>("Drug-Related Side Effects and Adverse Reactions"[Mesh] OR "poisoning"[Mesh] OR "Safety"[Mesh] OR "drug interactions"[Mesh] OR "contraindications"[Mesh] OR "abnormalities, drug induced"[Mesh] OR "Chemical and Drug Induced Liver Injury"[Mesh] OR "dyskinesia, drug-induced"[Mesh] OR "Cardiotoxins"[Mesh] OR "drug hypersensitivity"[Mesh] OR "drug eruptions"[Mesh] OR "drug Misuse"[Mesh] OR "Drug Tolerance"[Mesh] OR "Drug Resistance"[Mesh:NoExp] OR "Treatment Failure"[Mesh] OR "toxicogenetics"[Mesh] OR "product surveillance, postmarketing"[Mesh] OR "adverse drug reaction reporting systems"[Mesh] OR “pharmacovigilance”[Mesh] OR "toxicity tests"[Mesh] OR "toxic actions"[Mesh] OR "substance-related disorders"[Mesh] OR "Metabolic Side Effects of Drugs and Substances"[Mesh] OR “Toxicokinetics”[Mesh] OR “risk”[Mesh] OR "Environmental Exposure"[Mesh] OR “death”[Mesh] OR "adverse effects"[SH] OR "toxicity"[SH] OR "poisoning"[SH] OR "chemically induced"[SH] OR "case reports"[PT] OR "reproduction/drug effects"[Mesh] OR "birth weight/drug effects"[Mesh] OR "pregnancy/drug effects"[Mesh] OR "Pregnancy Complications/drug effects"[Mesh] OR "Breast Feeding"[Mesh] OR "Lactation"[Mesh] OR "fertility/drug effects"[MeSH] OR "fetus/drug effects"[Mesh] OR "Embryonic Structures/drug effects"[Mesh] OR "heart/drug effects"[Mesh] OR “liver/drug effects”[Mesh] OR "pancreas/drug effects"[Mesh] OR "Kidney/drug effects"[Mesh] OR "Lung/drug effects"[Mesh] OR "Blood/drug effects"[Mesh] OR adverse[TW] OR "untoward effect"[TW] OR "untoward effects"[TW] OR "untoward reaction"[TW] OR "untoward reactions"[TW] OR "untoward event"[TW] OR "untoward events"[TW] OR "side effects"[TW] OR "side effect"[TW] OR "side reaction"[TW] OR "side reactions"[TW] OR "side event"[TW] OR "side events"[TW] OR risk[TW] OR risks[TW] OR interaction[TW] OR interactions[TW] OR pharmacovigilance[TW] OR safety[TW] OR cardiotox*[TW] OR hepatotox*[TW] OR hepatox*[TW] OR pancreatox*[TW] OR genotox*[TW] OR nephrotox*[TW] OR pneumotox*[TW] OR neurotox*[TW] OR hematotox*[TW] OR haematotox*[TW] OR

pharmacotox*[TW] OR immunotox*[TW] OR immunocytotox*[TW] OR cytotox*[TW] OR toxi*[TW] OR tox[TW] OR lc50[TW] OR induced[TW] OR carcinogen*[TW] OR terat*[TW] OR mutagen*[TW] OR "reproduction study"[TW] OR "reproduction studies"[TW] OR pregnan*[TW] OR "breast-feeding"[TW] OR breastfeeding[TW] OR lactation[TW] OR fertility[TW] OR fetus[TW] OR embryo[TW] OR cancerogenicit*[TW] OR withdrawal[TW] OR contraindication[TW] OR "contraindications"[TW] OR "contra-indication"[TW] OR "contra-indications"[TW] OR "misuse"[TW] OR "drug resistance"[TW] OR "abuse"[TW] OR "overdos*"[TW] OR intoxication[TW] OR poison*[TW] OR death[TW] OR deaths[TW] OR fatal[TW] OR "case report"[TW] OR "case reports"[TW] OR "DRESS syndrome"[TW] OR "Dress syndromes"[TW] OR "Drug Hypersensitivity"[TW] OR "drug hypersensitivities"[TW] OR "Stevens Johnson Syndrome"[TW] OR "Stevens Johnson Syndromes"[TW] OR "Lyell's Syndrome"[TW] OR "Lyell's Syndromes"[TW] OR "Lyell Syndrome"[TW] OR "Lyell Syndromes"[TW] OR "Anticholinergic Syndrome"[TW] OR "Anticholinergic Syndromes"[TW])

Embase

(exp 'contrast medium'/ OR 'contrast media':de,ti,ab,kw OR 'contrast medium':de,ti,ab,kw OR 'contrast agent':de,ti,ab,kw OR 'contrast agents':de,ti,ab,kw OR 'contrast material':de,ti,ab,kw OR 'contrast materials':de,ti,ab,kw OR 'radiocontrast media':de,ti,ab,kw OR 'radiocontrast medium':de,ti,ab,kw OR 'radiocontrast agent':de,ti,ab,kw OR 'radiocontrast agents':de,ti,ab,kw OR 'radiopaque media':de,ti,ab,kw OR 'radiopaque medium':de,ti,ab,kw OR 'radio-opaque media':de,ti,ab,kw OR 'radio-opaque medium':de,ti,ab,kw OR 'radiopaque agent':de,ti,ab,kw OR 'radiopaque agents':de,ti,ab,kw OR 'radio-opaque agent':de,ti,ab,kw OR 'radio-opaque agents':de,ti,ab,kw OR 'ultrasound enhancing agent':de,ti,ab,kw OR 'ultrasound enhancing agents':de,ti,ab,kw)
AND
(exp 'microbubble'/ OR microbubble:de,ti,ab,kw OR microbubbles:de,ti,ab,kw OR 'micro-bubble':de,ti,ab,kw OR 'micro bubbles':de,ti,ab,kw OR 'gas-filled particle':de,ti,ab,kw OR 'gas-filled particles':de,ti,ab,kw OR 'colloidal gas aphron':de,ti,ab,kw OR 'colloidal gas aphrons':de,ti,ab,kw

OR exp 'sulfur hexafluoride'/ OR SonoVue®:ti,ab,tn,kw OR Lumason®®*:ti,ab,tn,kw OR 'contrast agent br1':ti,ab,tn,kw OR exp 'perflutren'/ OR 'fs 069':ti,ab,tn,kw OR 'fs069':ti,ab,tn,kw OR 'perfluoropropane-filled albumin microsphere solution':ti,ab,kw OR Optison®*:ti,ab,tn,kw OR luminity:ti,ab,tn,kw OR Definity®:ti,ab,tn,kw

OR exp 'perflubutane'/ OR 'Sonazoid®':ti,ab,tn,kw OR 'nc100100':ti,ab,tn,kw OR 'dd-723':ti,ab,tn,kw)
AND
(exp 'adverse drug reaction'/ OR exp 'poisoning'/ OR exp 'drug safety'/ OR exp 'drug interaction'/ OR exp 'drug contraindication'/ OR exp 'treatment contraindication'/ OR exp 'drug induced malformation'/ OR exp 'congenital disorder'/ OR exp 'chemical and drug induced liver injury'/ OR exp 'cardiotoxin'/ OR exp 'drug misuse'/ OR exp 'drug tolerance'/ OR exp hypersensitivity/ OR 'drug resistance'/de OR exp 'treatment failure'/ OR exp 'toxicogenetics'/ OR exp 'postmarketing surveillance'/ OR exp 'toxicity testing'/ OR exp 'carcinogenicity'/ OR exp 'toxicity and intoxication'/ OR exp 'substance-related disorders'/ OR exp 'toxicokinetics'/ OR exp 'occupational exposure'/ OR exp 'death'/ OR exp 'case report'/ OR exp 'reproduction'/ OR exp 'birth weight'/ OR exp 'pregnancy'/ OR exp 'pregnancy complications'/ OR exp 'breast feeding'/ OR exp 'lactation'/ OR (exp 'milk human'/ AND exp 'secretion'/) OR exp 'fertility'/ OR exp 'fetus'/ OR exp 'embryo'/

OR adverse:de,ab,ti,kw OR 'untoward effect':de,ti,ab,kw OR 'untoward effects':de,ti,ab,kw OR 'untoward reaction':de,ti,ab,kw OR 'untoward reactions':de,ti,ab,kw OR 'untoward event':de,ti,ab,kw OR 'untoward events':de,ti,ab,kw OR 'side effect':de,ab,ti,kw OR 'side effects':de,ab,ti,kw OR 'side reaction':de,ti,ab,kw OR 'side reactions':de,ti,ab,kw OR 'side event':de,ti,ab,kw OR 'side events':de,ti,ab,kw OR interaction:de,ti,ab,kw OR interactions:de,ti,ab,kw OR pharmacovigilance:de,ti,ab,kw OR safety:de,ti,ab,kw OR cardiotox*:de,ab,ti,kw OR hepatotox*:de,ab,ti,kw OR hepatox*:de,ti,ab,kw OR pancreatox*:de,ti,ab,kw OR genotox*:de,ti,ab,kw OR nephrotox*:de,ab,ti,kw OR pneumotox*:de,ti,ab,kw OR neurotox*:de,ti,ab,kw OR hematotox*:de,ti,ab,kw OR haematotox*:de,ti,ab,kw OR pharmacotox*:de,ab,ti,kw OR immunotox*:de,ab,ti,kw
OR immunocytotox*:de,ab,ti,kw OR cytotox*:de,ab,ti,kw OR toxic*:de,ab,ti,kw OR tox:de,ab,ti,kw OR lc50:de,ab,ti,kw OR induced:de,ti,ab,kw OR carcinogen*:de,ab,ti,kw OR terat*:de,ab,ti,kw OR mutagen*:de,ab,ti,kw OR 'reproduction study':de,ti,ab,kw OR 'reproduction studies':de,ti,ab,kw
OR pregnan*:de,ab,ti,kw OR 'breast feeding':de,ti,ab,kw OR 'breastfeeding':de,ti,ab,kw OR lactation:de,ti,ab,kw OR fertility:de,ti,ab,kw OR fetus:de,ab,ti,kw OR embryo:de,ab,ti,kw OR cancerogenicit*:de,ti,ab,kw OR withdrawal:de,ti,ab,kw OR contraindication:de,ti,ab,kw OR 'contra-indication':de,ti,ab,kw OR contraindications:de,ti,ab,kw OR 'contra-indications':de,ti,ab,kw OR misuse:de,ab,ti,kw
OR 'drug resistance':de,ti,ab,kw OR abuse:de,ti,ab,kw OR overdos*:de,ab,ti,kw OR intoxication:de,ti,ab,kw OR poison*:de,ti,ab,kw OR death*:de,ab,ti,kw OR fatal*:de,ab,ti,kw OR 'case report':de,ab,ti,kw OR 'case reports':de,ab,ti,kw OR 'dress syndrome':de,ab,ti,kw
OR 'dress syndromes':de,ab,ti,kw OR 'drug hypersensitivity':de,ab,ti,kw
OR 'drug hypersensitivities':de,ab,ti,kw
OR 'anaphylaxis':de,ti,ab,kw
OR 'anaphylactic':de,ti,ab,kw
OR 'anaphylactoid':de,ti,ab,kw OR 'stevens johnson syndrome':de,ab,ti,kw OR 'stevens johnson syndromes':de,ab,ti,kw OR 'lyell s syndrome':de,ab,ti,kw OR 'lyell s syndromes':de,ab,ti,kw OR 'lyell syndrome':de,ab,ti,kw OR 'lyell syndromes':de,ab,ti,kw OR 'anticholinergic syndrome':de,ab,ti,kw OR 'anticholinergic syndromes':de,ab,ti,kw)

Scopus

("Contrast Media" OR "contrast medium"
OR "contrast agent" OR "contrast agents"
OR "contrast material" OR "contrast materials"
OR "radiocontrast media" OR "radiocontrast medium"
OR "radiocontrast agent" OR "radiocontrast agents"
OR "radiopaque media" OR "radiopaque medium"
OR "radio-opaque media" OR "radio-opaque medium"
OR "radiopaque agent" OR "radiopaque agents" OR "radio-opaque agent" OR "radio-opaque agents"
OR "ultrasound enhancing agent" OR "ultrasound enhancing agents")
AND
(Microbubbles OR Microbubble
OR micro-bubble OR micro-bubbles OR microbubbles
OR "Gas-filled particle" OR "Gas-filled particles"
OR "Colloidal Gas Aphron" OR "Colloidal Gas Aphrons"
OR "contrast agent BR1" OR "contrast agent BR1" OR SonoVue®* OR Lumason®®*
OR "FS 069" OR "FS 069" OR FS069
OR "perfluoropropane-filled albumin microsphere solution" OR Optison®* OR Definity® OR Definity® OR Luminity

OR Sonazoid® OR Sonazoid® OR NC100100 OR DD-723)

AND
("Drug-Related Side Effects and Adverse Reactions" OR poisoning OR Safety
OR "drug interactions" OR contraindications
OR "abnormalities, drug induced" OR "Chemical and Drug Induced Liver Injury"
OR "dyskinesia, drug-induced"
OR Cardiotoxins OR "drug hypersensitivity" OR "drug eruptions" OR "drug Misuse" OR "Drug Tolerance" OR "Drug Resistance"
OR "Treatment Failure" OR toxicogenetics
OR "product surveillance, postmarketing"
OR "adverse drug reaction reporting systems" OR pharmacovigilance
OR "toxicity tests" OR "toxic actions" OR "substance-related disorders"
OR "Metabolic Side Effects of Drugs and Substances"
OR Toxicokinetics OR risk OR "Environmental Exposure" OR death
OR "Adverse Effects" OR "Toxicity" OR "Poisoning"
OR "Chemically Induced" OR "case reports"
OR "reproduction/drug effects"
OR "birth weight/drug effects" OR "pregnancy/drug effects"
OR "Pregnancy Complications/drug effects" OR "Breast Feeding"
OR Lactation OR "fertility/drug effects"
OR "fetus/drug effects" OR "Embryonic Structures/drug effects"
OR "heart/drug effects" OR "liver/drug effects" OR "pancreas/drug effects"
OR "Kidney/drug effects" OR "Lung/drug effects"
OR "Blood/drug effects" OR adverse
OR "untoward effect" OR "untoward effects" OR "untoward reaction"
OR "untoward reactions" OR "untoward event" OR "untoward events"
OR "side effects" OR "side effect" OR "side reaction" OR "side reactions"
OR "side event" OR "side events"
OR risk OR risks OR interaction OR interactions OR pharmacovigilance
OR safety OR cardiotox* OR hepatotox* OR hepatox* OR pancreatox*
OR genotox* OR nephrotox* OR pneumotox* OR neurotox* OR hematotox*
OR haematotox* OR pharmacotox* OR immunotox* OR immunocytotox*
OR cytotox* OR toxi* OR tox OR lc50 OR induced OR carcinogen* OR terat*
OR mutagen* OR "reproduction study" OR "reproduction studies" OR pregnan*
OR breast-feeding OR breastfeeding OR lactation OR fertility OR fetus OR embryo
OR cancerogenicit* OR withdrawal OR contraindication
OR contraindications OR contra-indication
OR contra-indications OR misuse OR "drug resistance" OR abuse OR overdos*
OR intoxication OR poison* OR death
OR deaths OR fatal OR "case report" OR "case reports" OR "DRESS syndrome" OR "Dress syndromes" OR "Drug Hypersensitivity" OR "drug hypersensitivities" OR "Stevens Johnson Syndrome" OR "Stevens Johnson Syndromes" OR "Lyell's Syndrome" OR "Lyell's Syndromes" OR "Lyell Syndrome" OR "Lyell Syndromes" OR "Anticholinergic Syndrome" OR "Anticholinergic Syndromes")

Table S4 - Study Characteristics of the included studies

Author Year	Country	Contrast Agent	Multi center	Study Design	Study Period	Age/Mean	Participants Total	Examinations Total	Male Female	Severe Adverse Events	Confounders (Present)Yes/No
Abdelmoneim 2009	United States	Optison® or Definity®	No	Observational Retrospective	November 2003-December 2007	65.8±12.1	10,792	10,792	6,193/4,598	0	Yes
Abo-Salem 2014	United States	Definity®	No	case report		39	1	1	0/1	0	No
Anantharam 2009	United Kingdom	Definity®	No	Observational Retrospective	January 2004-July 2008		461	621		0	Yes
Anantharam 2009	United Kingdom	SonoVue®	No	Observational Retrospective	January 2004-July 2008		689	529		0	Yes
Argulian 2020	United States	Optison®	No	Observational Retrospective		66	33	33	19/14	0	Yes
Baracchini 2017	Italy	SonoVue®	No	Observational Prospective	June 2014-June 2016		92	92		0	Yes
Bogdahn 1999	Germany	SonoVue®	Yes	Experimental Studies		60.8	10	40		0	No
Bokor 2001	United Kingdom	SonoVue®	No	Experimental Studies		25	44	90	44/0	0	No
Bokor 2001	United Kingdom	SonoVue®	No	Experimental Studies		68	12	12	8/4	0	Yes
Chou 2019	Taiwan	Sonazoid®	Yes	Observational Prospective	May 2014-42095		54	54		0	No
Cohen 1998	United States	Optison®	Yes	Experimental Studies			198	198		0	Yes
Coleman 2014	United States	Optison®	No	Observational Prospective	June 2003-January 2013		32	126		0	Yes
Cvitkovic-Roic 2022	Croatia	SonoVue®	No	Observational Retrospective	January 2013-February 2020		5153	5153		0	Yes
Dietrich 2021	Multiple Countires across Europe	SonoVue®	Yes	Observational Prospective			1,676	1,676		2	No
Dolan 2009	United States	Optison® or Definity®	Yes	Observational Prospective	1999-2007		23,659	23,659		0	No
Dolan 2009	United States	Definity®	Yes	Observational Prospective	1999-2007		5,947	5,947		0	Yes
Dolan 2009	United States	Optison®	Yes	Observational Prospective	1999-2007		12,802	12,802		0	Yes

Duran 2012	Spain	SonoVue®	No	Observational Retrospective	June 2009-January 2011	2.26±3.54	295	307	154/153	0	Yes
Faizah 2015	Malaysia	SonoVue®	No	Observational Prospective	July 2011-January 2013	2.1	27	27	17/10	0	No
Fang 2004	China	SonoVue®	Yes	Experimental Studies		62.8	90		51/39	0	No
Fischer 2020	Germany	SonoVue®	No	Observational Retrospective	2012-2019	55.4±17.5	764	2268	428/336	0	Yes
Furtado 2021	Brazil	SonoVue®	No	Observational Prospective		65*	1,062	1,062		0	Yes
Geleijnse 2009	The Netherlands	SonoVue®	No	Observational Prospective	2001-2005		274	352		3	No
Geyer 2020	Germany	SonoVue®	No	case series	June 2020-October 2020	34	5	11	0/5	0	No
Herzog 2008	United States	Optison®	No	Observational Prospective	February 1998-February 2002		3,051	3,051		0	Yes
Herzog 2008	United States	Definity®	No	Observational Prospective	February 1998-February 2002	56	6,959	6,959		2	No
Herzog 2008	United States	Definity®	No	Observational Prospective	February 1998-February 2002	56	5,231	5,231		2	No
Herzog 2008	United States	Definity®	No	Observational Prospective	February 2002-October 2007	56	784	784		0	No
Hu 2019	China	SonoVue®	No	Observational Prospective	January 2012-December 2018	46.2±15.5	34,478	344,78	20,348/14,130	3	No
Kaps 1999	Germany	SonoVue®	No	Experimental Studies	August 1998-December 1998		12	48	8/4	0	No
Kaps 2001	Germany, Netherlands, UK (3 European centers)	SonoVue®	Yes	Experimental Studies	June 2000-March 2001	63.4	40	160	21/19	0	No
Kim 2021	South Korea	SonoVue®	No	Observational Prospective	43556-October 2019	1.42*	32	32	20/12	0	Yes
Kis 2010	Hungary	SonoVue®	No	Observational Prospective		0.63	183	183	94/89	0	Yes
Kitzman 2000	United States	Definity®	Yes	Experimental Studies	December 1999-March 2000	56	169	338	139/72	0	Yes
Kljucevsek 2011	Slovenia	SonoVue®	No	Observational Prospective	February 2010-July 2010	0.42	66	66	35/31	0	Yes
Knieling 2016	Germany	SonoVue®	No	Observational Retrospective	38565-42309	11.4±7.5	40	79	19/21	0	No
Kumar 2019	United States	Definity®	No	prevalence			3,306	3,306		0	Yes
Kumar 2019	United States	SonoVue®	No	prevalence			2,137	2,137		0	Yes

Kumar 2019	United States	Optison®	No	prevalence			78	78		0	Yes
Kusnetzky 2008	United States	Definity®	No	Observational Prospective	January 2005-October 2007	66.1±15	6,196	6,196		0	Yes
Kutty 2016	United States	Definity®	No	Observational Prospective	January 2005-June 2014	17.8±3	113	113	62/51	0	Yes
Kuzmanovska 2017	Macedonia	SonoVue®	No	Observational Retrospective		6.4±4.9	31	31	7/24	0	Yes
Li 2023	China	SonoVue®	No	Observational Retrospective	January 2019-October 2021	46.61±12.68	49,100	49,100	15,033/34,067	7	No
Li 2024	China	SonoVue®	No	Observational Retrospective	December 2011-March 2023	51	83,778	83,778	47,048/36,730	5	No
Lv 2021	China and Korea	Sonazoid®	Yes	Observational Prospective	May 2014-42095		218	218		0	No
Lv 2021	China and Korea	SonoVue®	Yes	Observational Prospective	May 2014-42095		206	206		0	No
Madjar 2000	Germany, Italy, Switzerland	SonoVue®	Yes	Experimental Studies		54.9±13.7	40	160	0/40	0	Yes
Mao 2019	China	SonoVue®	No	Observational Prospective	July 2016-43556	7.8*	312	596	179/133	0	No
Marschner 2021	Germany	Sonazoid®	No	Observational Retrospective	2016-2020		49	49	12/37	0	No
McMahon 2005	United States	Optison®	No	Observational Prospective	January 2000-July 2002	15*	20	20	12/8	0	No
Miyamoto 2014	Japan	Sonazoid®	Yes	Experimental Studies	September 2010-June 2011	48.5±12.3	117	351	0/117	0	No
Morel 2000	Switzerland	SonoVue®	No	Experimental Studies	May 1999-August 1999		12	24	7/5	0	No
Nanda 2002	United States	SonoVue®	Yes	Experimental Studies		56.8±13.4	138	138	89/49	0	No
Nguyen 2025	Europe, Asia, Canada	SonoVue®	Yes	Observational Retrospective	2019-2023	35	15	15		0	No
Ntoulia 2018	United States	Optison®	No	Observational Retrospective	September 2014-42095	3.5±4.3	30	30	9/21	0	Yes
Ntoulia 2024	USA	Optison®	No	Observational Prospective	January 2013-June 2023		12	17	2/10	0	Yes
Ntoulia 2024	USA	SonoVue®	No	Observational Prospective	January 2013-June 2023	4.6	4		1/3	0	Yes
Nucifora 2008	Italy	Definity®	No	prevalence		58±11	115	115	88/27	0	Yes
Papadopoulou 2009	Greece	SonoVue®	No	Observational Prospective		1.92	228	456	123/105	0	Yes

Papadopoulou 2014	Greece	SonoVue®	No	Observational Retrospective	May 2009-September 2012	2.9	1010	1010	447/563	0	Yes
Piscaglia 2006	Italy	SonoVue®	Yes	Observational Retrospective	December 2001-December 2004		23188	23188		2	No
Piskunovicz 2015	Poland	SonoVue®	No	Observational Prospective	January 2013-February 2020	10.2±6	137	167	83/54	1	No
Platts 2013	Australia	Definity®	Yes	Observational Prospective	August 2007-May 2012	61.5±13.4	5,576	5956	3,545/2,411	0	Yes
Riggs 2024	United States	SonoVue®	Yes	Observational Retrospective	November 2016-44287	0.58*	38	47	19/19	0	Yes
Sauer 2017	Germany	SonoVue®	Yes	Observational Retrospective	2011-2015	4.9	531	531	53/478	0	Yes
Schwarze 2020	Germany	SonoVue®	No	case series	2005-2014	32	6	6	0/6	0	No
Schwarze 2021	Germany	SonoVue®	No	case series	May 2019-February 2020	31	5	6	0/5	0	No
Seelbach 2022	Germany	SonoVue®	No	Observational Prospective		10.5	30	30	15/15	0	No
Seelbach 2022	Germany	SonoVue®	No	Observational Prospective		3.1	56	56	24/32	0	Yes
Senior 2000	United Kingdom	SonoVue®	Yes	Observational Prospective		64±10	218	218	163/55	0	No
Shaikh 2008	United States	Optison® or Definity®	Yes	Observational Retrospective	December 21, 1999-November 2007	61*	2,914	2,914	1,555/1,359	1	Yes
Shang 2023	China	SonoVue®	Yes	Observational Retrospective	January 2016-43556		463,434	463,434		17	No
Siomou 2020	Greece	SonoVue®	No	Observational Prospective		0.18	60	60	44/16	0	Yes
Soman 2000	Austria	SonoVue®	No	Experimental Studies			13	26		0	Yes
Stenzel 2013	Germany	SonoVue®	No	case series		11.1	37	37		0	No
Suma 2016	United States	Definity®	Yes	Observational Prospective			179	179		0	Yes
Suma 2016	United States	Optison®	Yes	Observational Prospective			40	40		0	Yes
Timperley 2005	United Kingdom and France	SonoVue®	Yes	Observational Prospective	January 2002-July 2004	64±12	299	299	179/120	0	Yes
Timperley 2005	United Kingdom and France	Optison®	Yes	Observational Prospective	January 2002-July 2004	65±11	120	120	70/50	0	Yes

Torres 2017	Sweden	SonoVue®	No	Observational Retrospective	January 2004-December 2014	11	173	287		0	No
Wei 2008	United States	Definity®	Yes	Observational Prospective	January 2001-September 2007		66,164	66,164		5	Yes
Wei 2008	United States	Optison®	Yes	Observational Prospective	January 2001-September 2007		12219	12219		0	Yes
Wei 2012	United States	Definity®	Yes	prevalence	August 2009-December 2009	56*	32	32	15/17	0	No
Wei 2014	United States	Optison®	Yes	Observational Prospective	June 2008-March 2009	60*	1,039	1,039	644/395	0	Yes
Weiss 2012	United States	Definity®	Yes	Observational Prospective	39498-39929	61±12.9	1,053	1,053	653/400	0	Yes
Wever-Pinzon 2012	United States	Definity®	Yes	Observational Prospective	2001-2011	69±14	1,513	1,513	829/684	0	Yes
Wong 2014	Hong Kong	SonoVue®	No	Observational Prospective	September 2010-February 2012	0.5*	31	31	23/8	0	Yes
Wu 2023	United States	Definity®	Yes	Observational Prospective		26*	55		25/30	0	Yes
Wu 2023	United States	Definity®	Yes	Observational Prospective		33*	43		25/18	0	No
Zhang 2018	China	SonoVue®	No	Observational Prospective	August 2016-October 2017	3.1	90	90	43/47	0	No

Table S5 - Meta-regression results

Outcome	Variable	Level	Estimate Log (OR)	95% CI (log OR)	Std. Error	P-value
Adults - serious	Study design grouped	Observational Prospective vs experimental studies	-2.92	-4.27, -1.58	0.69	0.00
Adults - serious	Study design grouped	Observational Retrospective vs experimental studies	-3.70	-5.24, -2.15	0.79	0.00
Adults - serious	Study design grouped	Prevalence vs experimental studies	-1.86	-4.02, 0.3	1.10	0.09
Adults - serious	Confounding factors	Yes vs No	-0.22	-1.26, 0.82	0.53	0.68
Children IV - serious	Study design grouped	Observational Prospective vs experimental studies	-0.67	-3.92, 2.58	1.66	0.69
Children IV - serious	Study design grouped	Observational Retrospective vs experimental studies	-1.58	-4.94, 1.78	1.71	0.36
Children IV - serious	Confounding factors	Yes vs No	0.18	-0.99, 1.35	0.59	0.76
Adults - non serious	Study design grouped	Observational Prospective vs experimental studies	-3.45	-4.76, -2.14	0.67	0.00
Adults - non serious	Study design grouped	Observational Retrospective vs experimental studies	-3.92	-5.46, -2.39	0.78	0.00
Adults - non serious	Study design grouped	Prevalence vs experimental studies	-1.81	-3.8, 0.18	1.02	0.07
Children IV - non serious	Study design grouped	Observational Prospective vs experimental studies	-0.08	-3.22, 3.06	1.60	0.96

Children IV - non serious	Study design grouped	Observational Retrospective	-1.37	-4.65, 1.9	1.67	0.41
Children IV - non serious	Confounding factors	Yes vs No	-0.72	-1.95, 0.52	0.63	0.25
Children endocavitary - non serious	Study design grouped	Observational Retrospective vs experimental studies	-1.38	-2.79, 0.02	0.72	0.05
Children endocavitary - non serious	Confounding factors	Yes vs No	-0.74	-2.56, 1.07	0.93	0.42
Adults - Delayed adverse reactions	Study design grouped	Observational Prospective vs experimental studies	-3.30	-4.78, -1.83	0.75	0.00
Adults - Delayed adverse reactions	Study design grouped	Observational Retrospective	-4.37	-6.14, -2.6	0.90	0.00
Adults - Delayed adverse reactions	Study design grouped	Prevalence vs experimental studies	-2.64	-5.14, -0.14	1.27	0.04
Adults - Delayed adverse reactions	Confounding factors	Yes vs No	0.17	-1.05, 1.4	0.62	0.78
Children Endocavitary - Delayed adverse reactions	Study design grouped	Observational Prospective vs experimental studies	-3.30	-4.78, -1.83	0.75	0.00
Children Endocavitary - Delayed adverse reactions	Study design grouped	Observational Retrospective vs experimental studies	-4.37	-6.14, -2.6	0.90	0.00
Children Endocavitary - Delayed adverse reactions	Study design grouped	Prevalence vs experimental studies	-2.64	-5.14, -0.14	1.27	0.04
Children Endocavitary - Delayed adverse reactions	Confounding factors	Yes vs No	0.17	-1.05, 1.4	0.62	0.78

Table S6 - Case reports reporting treatment of serious adverse events

Author year	N	Adverse events	Contrast agent	Echocardio	Patient characteristics	Symptoms	Treatment	Appropriateness (Yes/No)	Onset	Notes
Geleijnse 2009	352	1	SonoVue®	Yes	80/M, known hypertension and recent (3 weeks earlier) carotid stenting.	Extensive skin erythema and shock (BP drop from 150/70 to 70/30 mmHg).	Clemastine 2 mg + hydrocortisone 100 mg IV + 300 ml gelofusine.	No	Immediately	
Geleijnse 2009	352	2	SonoVue®	Yes	68/F, with known Pompe's disease	Tinnitus, dizziness, BP drop (140/80 to 70/40), sinus bradycardia (90 to 50 bpm), loss of consciousness.	Clemastine 2 mg + hydrocortisone 100 mg IV + 150 ml gelofusine.	No	Immediately	
Geleijnse 2009	352	3	SonoVue®	Yes	34/M, with known familiar non-ischemic dilated cardiomyopathy	Nausea, extensive skin erythema, sinus bradycardia (<30 bpm), unconscious, gasping.	Clemastine 2 mg + hydrocortisone 100 mg IV + cardiac massage + manual ventilation.	Yes	Immediately	Immunological analysis revealed a type III allergic reaction without identification of a specific allergen.
Herzog 2008	6959	1	Definity®	Yes, prestress with Dobutamina	58/F, with Pulmonary hypertension, DM, Hepatitis C, Allergies Sulfa (pruritus), codeine (pruritus)	Back and chest pain with transient arterial desaturation	None	No	7 min	
Herzog 2008	6959	2	Definity®	Yes	45/F, with hepatitis	Chest pain with dyspnea,	Oxygen, 30 min	Yes	1-2 min	
Herzog 2008	6959	3	Definity®	Yes, prestress	25-year-old chronic kidney disease, hypertension. Allergy to gadolinium (prurutus)	Dyspnea with chest And back pain	None	No	1-2 min	

Herzog 2008	6959	4	Definity®	Yes	59/F with renal cell cancer, hypertension, DM. Allergy to cephalosporin	Back pain, dyspnea, bronchospasm	Bronchodilator	Yes	1 min
Hu 2019	34478	1	SonoVue®			Anaphylactic shock (skin erythema)	IM epinephine (1mg/ml) 0,3-0,5 mg Oxygen 8-10 L/min	Yes	Median time 13 min
Hu 2019	34478	2	SonoVue®			Anaphylactic shock (nausea, dizziness)	IM epinephine (1mg/ml) 0,3-0,5 mg Oxygen 8-10 L/min	Yes	Median time 13 min
Piscaglia 2006	23188	1	Definity®	Yes	71/M, previously operated on for prostate cancer, being investigated to characterise a liver lesion suspected of metastasis	Dyspnoea, bronchospasm, slight hypotension and bradycardia	Hydrocortisone - 2g total Intravenous (IV) chlorophenamine	No	1 min
Piscaglia 2006	23188	2	Definity®	Yes	65/F, who suffered from atherosclerotic vasculopathy and had a stent positioned in the right renal artery for a severe ostial stenosis	Clouding of consciousness followed by dursolumbar pain. The pulse accelerated first and then quickly became immeasurable. Severe hypotension lasted over 30 min despite further medical treatment. 15 min after contrast medium administration, a cutaneous rash occurred.	1g hydrocortisone + vasoactive amines	No	2 min
Shang 2023	463434	17*	SonoVue®		Avg. Age 47.5, 50/50 gender	Anaphylactic shock (hypotension + tachycardia) (n = 13). In 1 case, it was followed by	Standard treatments for the severe physiologic reactions involved	No	91% within 30 min

						wheezing/bronchospasm, significant hypoxia. For patients with severe physiologic aes, the most common signs and symptoms were vasovagal reaction resistant to treatment (n = 2) and followed by arrhythmia (n = 1).	papaverine, saline, and potassium chloride, dexamethasone, saline, and potassium chloride for another; blood transfusion, correction of coagulation abnormalities, liver protection, and anti-infection treatment. dexamethasone glucose, potassium chloride, and calcium gluconate.			
Tang 2017	30222	1	SonoVue®		47/M	Blurred vision, chest tightness, palpitations, small amount of nasal bleeding, hypotension, dyspnea	Inhalation oxygen,intravenous injection of dexamethasone sodium phosphate (10 mg)	Yes	1 min	2 hours later, his blood pressure and heart rate were back to normal. The patient was followed on the next day, and he did not report any discomfort.
Tang 2017	30222	2	SonoVue®		35/M	Chest tightness, palpitations, facial cyanosis, dyspnea, hypotension, tachycardia	Inhalation oxygen, intravenous injection of epinephrine hydrochloride (1 mg)	Yes	1 min 20 sec	After 2 min, symptoms were back to normal; on the next day during follow-up by telephone, the patient did not report discomfort
Wei 2008	78383	1	Definity®	Yes	74/M	Anaphylactoid: hypotension, rash, diaphoresis, difficulty swallowing	Fluids, antihistamines, SQ epinephrine	Yes	2-3 min	Monitored for 4 to 8 hours in the hospital or the emergency department before being discharged

Wei 2008	78383	2	Definity ®	Yes	F	Anaphylactoid: hypotension, less responsive	Fluids	No	10-15 min	
Wei 2008	78383	3	Definity ®	Yes	55/M	Anaphylactoid: SOB, urticaria, throat swelling	Steroids, antihistamines	No	<1 min	
Wei 2008	78383	4	Definity ®	Yes	40/M	Anaphylactoid: hives, throat swelling, hypotension	SQ epi, antihistamines	No	< 2min	
Wei 2008	78383	8	Definity ®	Yes	59/F	Hypoxemia: low back pain, acute SOB, and O2 sat 80s	Bronchodilator	Yes	<1 min	
Blokh 2004	1	1	Luminity		83/F, history of CAD, DM, atrial fibrilation, pacemaker, recent sepsis	Less responsive, with systolic blood pressure dropping to 70 mmhg, and developed rapid atrial fibrillation at a rate of 130 beats/min Subsequently, her oxygen saturation dropped to 70%	Benadryl, Pepcid, Solumedrol, fluids, vasopressors, intubation	Yes	3 min	The next day the levophed was titrated off and both BUN and creatinine levels decreased to initial values. Three sets of serial cardiac enzymes were within normal limits.
Calvo 2006	175	1	SonoVue®	Yes, dobutamine	42/M, with a history of an acute anterior myocardial infarction 4 months previously, and single vessel coronary disease	Sudden sweating, rubefaction and bradycardia, followed by severe hypotension and cardiorespiratory arrest	Advanced CRP was initiated and four minutes later the patient recovered a stable hemodynamic situation	Yes	Immediately	Four minutes later, the patient recovered a stable hemodynamic situation
Coudray 2017	1	1	SonoVue®		62/M, with hypertension, without any other cardiopulmonary disease or allergic history	Limb paraesthesia, shortness of breath, then presented a rash, with profuse sweating and reported a sensation of intense heat inside and around his torso, but no chest pain. A few minutes later, his eyes rolled back into his head, and	Intravenous injection of 100g epinephrine and fluid resuscitation (500ml normal saline) A new intravenous administration of 100g epinephrine was required about 2 hours	Yes	30 sec	Transferred to the ICU he was discharged one day later. Increased levels of histamine up to 88,9nmol/L and tryptase up to 147ugl/L. During the following months, the search for allergens

						he lost consciousness and urinated, without clonic jerks. Blood pressure measurement was impossible. The patient desaturated down to 92% spo2.	later, with continuous relay because of a new hemo- dynamic and respiratory deterioration.			by skin prick tests was negative for SonoVue®®, the product itself and one of this excipient, macrogol.
D'Amico 2020	1	1	Definity ®	Yes	A 53/F, African American	Acute pain in her lower extremities that progressed caudally to her abdomen and chest, hypoxic and tachypneic Acute splenic sequestration crisis (ASSC) was suspected.	Emergency transfusion, hospitalization, oxygen, IV fluids.	Yes	Within min	Definity® use was identified as potential triggering factor.
Gupta 2015	1	1	Definity ®	Yes	66/F, known allergy to iodinated contrast	Angioedema (lip swelling), urticarial rash (chin, arm, leg), shortness of breath, difficulty swallowing.	Diphenhydramine 50 mg	Yes	20 min	Symptoms subsided over the next 1 hour. Allergy to iodinated cm
Krantz 2020	1	1	Definity ®	Yes	58/M, Prior known IgE-mediated HMW pegallergy confirmed via positive skin testing and anti-PEG IgE. Required ICU admission and IV epinephrine.	Anaphylaxis (urticaria, shortness of breath, hypotension)	Intramuscular epinephrine, steroids, and antihistamines, but ultimately required intravenous epinephrine drip and intensive care unit admission for continued hypotension	Yes	Within minutes	Skin Test + to PEG 3350 at all concentrations. He was SPT and oral challenge negative to PEG 300, a lower MW macrogol and methylprednisolone sodium succinate which does not contain PEG 3350. Stabilization over the 4 hours after admission led to weaning of his vasopressors, and he was discharged the following day.

Levano 2012	1	1	SonoVue®		39/F, with liver cirrhosis of unknown cause (Child-Pugh score B) and portal hypertension	Sensation in the upper trunk and neck, throat tightness, extensive skin erythema, dyspnea, cyanosis, and loss of consciousness. Hypotension (blood pressure, 50/30 mm Hg), tachycardia, and a silent chest, and her Glasgow Coma Scale score was 7	Intubated, mechanical ventilation, immediately administered intravenous fluids, corticosteroids, and antihistamines and norepinephrine	Yes	Immediately	Skin prick tests and intradermal tests were performed with SonoVue®, beginning with a dilute solution (1/10,000); the concentration was increased by 10-fold every 15 minutes until the concentration of the undiluted dose (5 mg/ml) was reached. All the skin test results were negative . Non–IgE–mediated anaphylactic shock Five hours later, the patient's signs and symptoms began to improve, and she was extubated. She was maintained under observation in the ICU for 24 hours.
Mahjoub 2009	1	1	Definity ®	Yes	73/M, with hypertension, dyslipidemia, chronic atrial fibrillation, a history of coronary artery disease with bypass	Cardiac arrest, with initially no electrical activity (asystole) followed by ventricular tachycardia on electrocardiographic monitoring	Cardiac resuscitative maneuvers were immediately performed but to no avail despite emergency thoracotomy, which revealed no effusion. The patient was pronounced dead after approximately 30 minutes of attempted	Yes	5 min	The postmortem examination confirmed there was no evidence of an acute allergic reaction. Pathologic examination did not reveal signs of microvascular leakage or cell injury consistent with damage due to microbubble rupture. Favoring pseudocomplication

Mansour 2022	1	1	Lumason®®	Yes	67/M, with a history of heart failure with preserved EF presented for an outpatient echocardiogram evaluation due to the gradual worsening of his dyspnea during exertion		cardiopulmonary resuscitation. Intravenous antihistamines, methylprednisolone and epinephrine	Yes	Immediately	
Mir 2021	1	1	Definity®	Yes	69/M, African American with history of hypertension, chronic atrial fibrillation on apixaban, heart failure with preserved EF, obstructive sleep apnea, chronic kidney disease stage II, DM and CAD with PCI to the ramus and a known right coronary artery (RCA) chronic total occlusion (CTO) with adequate collaterals	Severe dyspnoea and became diaphoretic with subsequent loss of consciousness. No electrical activity which was consistent with asystole.	Cardiac massage and epinephrine administration	Yes	Immediately	His condition stabilized and was monitored for a few hours to ensure that there were no delayed reactions. He was discharged home later that day.
Olson 2018	1	1	Lumason®®		63/M, myelodysplastic syndrome, confirmed by a bone marrow biopsy, and a	He became apneic and pulseless.	Chest compressions and bag-valve-mask ventilation. 1 mg of epinephrine, two	Yes	30 sec	Elevation of the patient's tryptase to 71.4 ng/ml, while urine analysis showed elevation of leukotriene E4 (6750

					reported diagnosis of indolent systemic mastocytosis,		ampules of bicarbonate and 1 g of calcium chloride. Given concern for an allergic reaction in the context of mastocytosis, the patient also received 10 mg of intravenous dexamethasone, 50 mg of diphenhydramine IV, and a total of 40 mg of famotidine IV.			pg/mg Cr; normal < 104) and N-methylhistamine (3897 mcg/g Cr; normal 30–200), all findings consistent with mast cell activation and systemic mastocytosis.
Solivetti 2012	1	1	SonoVue®		45/F, healthy woman with no history of cardiac or allergic disease.	Itching, heat sensation, edema of face and glottis, urticaria, severe dyspnea, anaphylactic shock with hypotension and circulatory collapse.	Oxygen, IV corticosteroids, antihistamines, plasma expanders.	No	3 min	Slow recovery; monitored until next day.Kept under observation by a medical intensivist until the evening; the cutaneous reaction and slight edema persisted until the following day.
Vanginkel 2014	1	1	SonoVue®	Yes	60/M	Allergic reaction (nausea, hypotension, sweating) → severe chest pain, total AV block, ST-segment elevation (inferior leads), coronary spasm confirmed via angiography and IVUS — diagnosed as Kounis syndrome.	Clemastine, hydrocortisone, oxygen, saline infusion.	No	1-2 minutes	Coronary spasm resolved spontaneously.
Arnouk 2023	1	1	Lumason®®	Yes	35/M	Feeling flushed and feeling like something was wrong. He then	CRP was initiated for PEA and the patient was intubated. An	Yes	Immediately	The patient died several hours later. His post-arrest troponin was 1.29 ng/

						became unresponsive and pulseless	automated chest compression device was utilized. Maximal support with Epinephrine, sodium bicarbonate, dextrose, calcium, magnesium, dexamethasone, and tissue plasminogen activator. After approximately 45 minutes of resuscitative efforts, the patient, still pulseless, was placed on VA ECMO with maximal support. He developed ventricular fibrillation that converted to a slow sinus rhythm after defibrillation and receipt of amiodarone. He developed worsening lactic acidosis and died several hours later.			ml. Autopsy was not performed. The Naranjo adverse drug reaction probability scale yielded a score of 3, indicating possible likelihood of a drug-induced adverse event.
Crucinio 2004	1	1	SonoVue®		77/F, Chronic disorders such as obstructive pulmonary disease,	Acute severe dyspnea and fall of blood pressure until undetectable values occurred.	Basic life support manouvres associated to steroid and plasma expander	Yes	5 min	The onset of the event we observed cannot be clearly established.

					hypertension, atrial fibrillation and DM a chronic liver disease with a dishomogeneous ultrasono- graphic pattern associated to a focal lesion		intravenous administration allowed reaching a normalization of cardiac and SonoVue®respiratory functions within 20 min.			Reaching a normalization of cardiac and respiratory functions within 20 min.
Steihauer 2023	1	1	SonoVue®	1	49/F, no known medical history, recent LAD stent placement; presented in heart failure, LVEF 30–35%, treated with dual antiplatelet therapy	Sudden onset dyspnea and increased work of breathing followed by unresponsiveness and loss of pulse	Necessitating initiation of CPR. On exam her skin was dusky and she developed a diffuse erythematous rash. She received two rounds of CPR with administration of two doses of 1 mg IV epinephrine, 200 mg IV hydrocortisone, and 50 mg IV diphenhydramine	Yes	Within minutes	Urgent coronary angiography revealed LAD stent thrombosis Allergy and Immunology: laboratory data were highly suggestive of an anaphylactic event. Tryptase was elevated at 23.9mcg/L (reference range <11mcg/L). May be an important relationship between anaphylaxis and acute coronary syndrome with in-stent thrombosis Kounis 1991, there is a known phenomenon coronary spasm or occlusive coronary disease in the setting of allergic events.
Ionescu 2009	1	1	SonoVue®	Yes	55/M, obese man with a history of type 2 DM on insulin and Metformin, documented three-vessel coronary artery disease and	Paresthesias over his face and head, and then severe dizziness, after which he became unresponsive. Sinus bradycardia (30 b.p.m.), and his systemic arterial	Atropine 600mg i.v. 200 mg of hydrocortisone and 10mg of chlorpheniramine 'free-running' infusion of normal saline At this point a tonicclonic	Yes	3 min	The causal link between the contrast agent and the side effects is established without doubt, and it highlights the fact that, like any other substance given to patients for diagnostic

					angioplasty to a diagonal branch of his LAD 6 weeks earlier	pressure was 60 mmhg systolic	fit and received 5 mg of Midazolam i.v. A faintly erythema- tous blanching rash visible on the patient's chest and abdomen			or treat- ment purposes, SonoVue® can cause life-threatening reactions.
Samannan 2018	1	1	Definity®	Yes	65/M, Caucasian with a history of asymptomatic atrial tachycardia, hypertension and hyperlipidemia, symptoms of dyspnea on exertion	Swelling of lips and wheezing	Epinephrine, IV Methyl prednisone and antihistamines and transferred to intensive care unit	Yes	15 sec	His respiratory distress subsided with above measure and was discharged home the next day. His chart was updated about allergy to echo contrast and the patient was told not to receive any contrast agents in future.
Piskunovicz 2015	137	1	SonoVue®		Pediatric oncology patients (mean age 10.2 years)	Severe anaphylaxis (hypotension, tachy/bradycardia, hypoxia)	Oxygen, IV epinephrine, IV fluids (nacl)	Yes	43 sec	After 2 h the patient's general condition improved, and all symptoms resolved.
Nomigolzar 2022	1	1	SonoVue®		74/F, history of anaphylaxis (penicillin and cephalosporins), provoked pulmonary embolism (PE), hypertension, and hypothyroidism	Chest pain, ECG changes consistent with myocardial ischemia, Type I Kounis Syndrome (coronary spasm without thrombosis), triggered by contrast Somnolent, hypotensive, and diaphoretic	Methylprednisolone, diphenhydramine, and famotidine	Yes	Immediately	Allergic reaction to this agent causing KS type I.
Sagalov 2023	1	1	SonoVue®	Yes	51/M, history of CAD, HF with reduced EF, LV thrombus, cocaine use, hypertension; prior exposure to SHLTAM (3rd time)	Chest pain, dyspnea, hypotension, lip/tongue swelling, diffuse urticaria, paradoxical breathing, wheezing, ST elevations, elevated troponin (Kounis Syndrome)	Epinephrine Solu-Medrol Benadryl Heparin drip Urgent cardiac catheterization	Yes	Immediately	A complete blood count with differential did not show elevated eosinophil counts before or after administration of the contrast agent. 15 minutes later showed

										resolution of the acute ST changes. Diagnosis: Kounis syndrome
Marchi 2024	1	1	SonoVue®	Yes	44/M, hypertension, recent ACS with stent in lcx; no history of allergies	Diffuse rash, hypotension (70/30), tachycardia (120 bpm), bronchospasm, chest pain, STEMI, AV block (2:1), stent thrombosis (moxed Type 1 and type 3 Kounis Syndrome), coronary spasm	IV hydrocortisone 500 mg IV chlorphenamine 10 mg Fluids (aggressive) IV norepinephrine (0.2 mg/kg/min) PCI with thrombectomy and new stent Avoiding epinephrine for the risk of worsening the myocardial ischemia and induction of coronary vasospasm or arrythmias.	Yes	Immediately	Mast cell tryptase levels and white blood cell formulae were measured on the first POD and resulted in the normal range.
Nigolian 2024	1	1	SonoVue®	Yes	60/M, history of ischemic cardiomyopathy, prior cardiac arrest, severe LV dysfunction (EF 25%), apical thrombus, cerebral palsy, dehydration, no known allergies	Rash (chest & abdomen), stridor, respiratory failure, hemodynamic instability, cardiac arrest, required defibrillation	IV antihistamines IV corticosteroids Oxygen IV fluids IV epinephrine CPR (25 min) Amiodarone Electrical defibrillation (2x)	Yes	~3 minutes	The serum tryptase level, measured 2hours after the onset of symptoms, was at 16μg/L (reference range: 0–11.4μg/L), which is consistent with anaphylactic shock. Due to the severity of the reaction, no skin tests were performed. Basophil activation test was positive for SonoVue® (sulphur hexafluoride and excipients like macrogol/PEG 2000)

										but negative for macrogol/ PEG 2000 alone.
Mikhail 2023	1	1	Lumason®®	Yes	50/M, PMH: hypertension, hyperlipidemia, atrial fibrillation, OSA, diabetes, asthma; prior Lumason®® exposure 3 weeks earlier without reaction	Acute anaphylaxis: severe shortness of breath, cyanosis, diaphoresis, tachycardia, hypoxia; wheezing on auscultation	IV Solumedrol 125 mg IV Diphenhydramine 50 mg Albuterol inhalation Oxygen (4 L/min via nasal cannula)	Yes	Within minutes	After 12 h of observation transfer to medical dept
Longhino 2024	1	1	SonoVue®		67/M, liver cirrhosis (Child–Pugh A6), hepatocellular carcinoma, portal hypertension; prior exposure to SonoVue® 20 days earlier without reaction	Pruritus, dyspnea, bilateral wheezing, cyanosis, cardiac arrest	IV methylprednisolone 80 mg IV chlorphenamine 10 mg CPR Laryngeal mask placement Midazolam infusion	Yes	~3 minutes	Skin prick tests and intradermal reactions with SonoVue® (1:10,000, 1:1000, 1:100, 1:10 and pure solutions were negative<br>BAT for SonoVue® was carried out and came back negative.
Amir-Kabirian 2024	1	1	Lumason®®	Yes	58/F, remote history of breast cancer (mastectomy, chemotherapy, radiation); allergy to penicillin (rash only); no known PEG allergy or anaphylaxis	Tingling/numbness around mouth, urinary incontinence, unresponsiveness, cardiac arrest (PEA), required ACLS and vasopressors	Epinephrine (1 dose during arrest) Solu-Medrol Diphenhydramine Vasopressors (ICU) Methylprednisolone, Diphenhydramine, Famotidine	Yes	Within minutes	Several days
Hadid 2024	1	1	Lumason®®	Yes	55/M, history of End Stage Renal Disease and hypertension; known allergy to iodinated contrast (premedicated for prior imaging)	Diffuse rash, erythema, pruritus, skin blanching, hypotension (BP dropped to 60s), wheezing, anaphylactic shock, required intubation	Intubation, full treatment for anaphylactic shock CT-Pulmonary Embolism after pre-medication	Yes	Within minutes	CT-Pulmonary Embolism after premedication with steroids for previous allergy to iodinated cm and antihistamines which

							with steroids and antihistamines			was unrevealing and uneventful.
Minc 2024	1	1	SonoVue®	Yes	67/F, moderate aortic stenosis; no PEG allergy, but diagnosed with hereditary alpha tryptasemia	Itching, urticaria, shortness of breath, loss of consciousness (LOC) → anaphylaxis	IM Epinephrine IV Diphenhydramine IV Methylprednisolone	Yes	Immediately	Skin prick test to PEG 3350 and UEA negative; tryptase elevated;hat testing :extrallelic copy of TBSB1
Dietrich 2021	1676	1	SonoVue®		11/F	Loss of consciousness, pruritus, nausea, hypotension, tachycardia/bradycardia, decreased oxygen saturation (60 %)	IV Epinephrine, oxygen, crystalloid bolus	Yes	43 sec	Symptoms resolved following treatment
Dietrich 2021	1676	1	SonoVue®		4/M, paediatric CEUS case	Widespread urticaria, cardiovascular collapse, and loss of consciousness	IM Adrenaline, IV Chlorpheniramine , IM/IV Hydrocortisone, crystalloid bolus, oxygen followed by intubation	Yes	55 sec	

Abbreviations
M, male; BP, blood pressure; F, Female; IV, intravenous; bpm, beats per minute; DM, diabetes mellitus; iM, intramuscular; ICU, intensive care unit; EF, ejection fraction; CPR, cardio pulmonary resuscitation; CAD, coronary artery disease; PCI, percutaneous coronary intervention; EF, ejection fraction; PEA, pulseless electrical activity; VA ECMO, veno-arterial extracorporeal membrane oxygenation

Table S7 - Characteristics of studies on pregnant women

Author year	Contrast Agent	Number of participants	Patient characteristics	Type of exam	Number of examinations	Number of AEs (severe, non-severe, delayed)
Schwarze 2021	SonoVue®	5	P1: 35 yearsold, 21 weeks, Desmoid tumor of the left rectus abdominis muscle.<br>P2: 35 years old, 33 weeks, Hepatic hemangioma in segment VI.<br>P3: 30 years old, 5 weeks, Hepatic amebic abscess in segment V.<br>P4: 30 years old, 21 weeks, Retroperitoneal and pelvic hematoma without active bleeding after high velocity car accident.<br>P5: 31 years old, 12 weeks, Renal cyst, Bosniak type 1, focal scar- ring of renal cortex after recurrent urinary tract infections	P1: MRI: predominantly T1-hypointense, T2-hyperintense<br>P3: MRI: predominantly T1-hypointense, T2-hyperintense, restricted diffusion<br>P4: MRI: retroperitoneal and pelvic hematoma. No visceral lacerations	6	0
Schwarze 2020	SonoVue®	6	P1: 25 years old, 24 weeks, Hepatic metastases, rectal cancer.<br>P2: 35 years old, 19 weeks, Hepatic atypical hemangioma. P3: 33 years old, 15 weeks, Focal nodular hyperplasia (FNH) .<br>P4: 34 years old, 12 weeks, Hepatic cystic echinococcosis<br>P5: 34 years old, 33 weeks, Hepatic arteriovenous malformation .<br>P6: 29 years old, 35 weeks, Hepatic metastases; acinus cell carcinoma of the pancreas.	MRI hepatic lesions	6	0

Geyer 2020	SonoVue®	5	P1: 30 years old, 27 weeks, Renal angiomyolipoma.<br>P2: 37 years old, 21 weeks, Pyelonephritis, ruled out the presence of an abscess.<br>P3: 34 years old, 25 weeks, Necrotic uterine fibroid.<br>P4: 33 years old, 17 weeks, Gallbladder polyp.<br>P5: 34 years old, 14 weeks, Superior mesenteric vein thrombosis.	P1: T1-hypointense, T2-hypointense, restricted diffusion<br>P2: homogeneous, restricted diffusion, perirenal fluid collections<br>P3: inhomogeneous, predominantly T1-hypointense, T2-hypointense<br>P5: small superior mesenteric vein thrombosis	11	0
Abo-Salem 2014	Definity®	1	39-year-old, 35 weeks, hypertensive urgency, chronic kidney disease, morbid obesity, remote history of syphilis	Transthoracic echocardiography (TTE), contrast echocardiography, chest CTA	1	0

Abbreviations

USCA, Ultrasound Contrast Agent; US, Ultrasound; AE, adverse event; ESUR-CMSC, ESUR Contrast Media Safety Committee; PICO, P (Population) I (Intervention) C (Comparison) O (Outcome); CEUS, Contrast-enhanced Ultrasound

Table S8 - JBI Critical Appraisal - Case Report

Author year	1) Were patient's demographic characteristics clearly described?	2) Was the patient's history clearly described and presented as a timeline?	3) Was the current clinical condition of the patient on presentation clearly described?	4) Were diagnostic tests or assessment methods and the results clearly described?	5) Was the intervention(s) or treatment procedure(s) clearly described?	6) Was the post-intervention clinical condition clearly described?	7) Were adverse events (harms) or unanticipated events identified and described?	8) Does the case report provide takeaway lessons?
Arnouk 2023	Yes	Yes	Yes	Yes	Yes	No	Yes	Yes
Blokh 2004	Yes	Yes	Yes	Yes	Yes	No	Yes	Yes
Calvo 2006	Yes	Yes	Yes	Yes	Yes	Yes	Yes	Yes
Crucinio 2004	Yes	Yes	Yes	Yes	Yes	Yes	Yes	Yes
D'Amico 2020	Yes	Yes	Yes	Yes	Yes	Yes	Yes	Yes
Gupta 2015	Yes	No	Yes	Yes	Yes	No	Yes	Yes
Ionescu 2009	Yes	Yes	Yes	Yes	Yes	Yes	Yes	Yes
Levano 2012	Yes	Yes	Yes	Yes	Yes	Yes	Yes	Yes

Mahjoub 2009	Yes	Yes	Yes	Yes	Yes	Yes	Yes	Yes
Mansour 2022	Yes	Yes	Yes	Yes	No	No	No	Yes
Mir 2021	No	Yes	Yes	Yes	Yes	Yes	Yes	Yes
Olson 2018	Yes	Yes	Yes	Yes	Yes	Yes	Yes	Yes
Portero-Portaz 2020	No	Yes	Yes	Yes	No	Yes	Yes	Yes
Schwarze 2020	Yes	Yes	Yes	Yes	Yes	No	Yes	Yes
Solivetti 2012	Yes	Yes	Yes	Yes	Yes	Yes	Yes	Yes
Steinhauer 2022	Yes	Yes	Yes	Yes	Yes	Yes	Yes	Yes
vanGinkel 2014	No	Yes	Yes	Yes	Yes	Yes	Yes	Yes
Abo-Salem 2014	Yes	Yes	Yes	Yes	Yes	Yes	Yes	Yes
Krantz 2020	Yes	Yes	Yes	Yes	Yes	Yes	Yes	Yes
Samannan 2018	Yes	Yes	Yes	No	Yes	Yes	Yes	Yes
Godart 2023	Yes	Yes	Yes	Yes	Yes	Yes	Yes	Yes

Nomigolzar 2022	Yes	Yes	Yes	Yes	Yes	Yes	Yes	Yes
Rames 2024	Yes	Yes	Yes	Yes	Yes	Yes	Yes	Yes
Sagalov 2023	Yes	Yes	Yes	Yes	Yes	Yes	Yes	Yes
Marchi 2024	Yes	Yes	Yes	Yes	Yes	Yes	Yes	Yes
Nigolian 2024	Yes	Yes	Yes	Yes	Yes	Yes	Yes	Yes
Mikhail 2023	Yes	Yes	Yes	Yes	Yes	Yes	Yes	Yes
Longhino 2024	Yes	Yes	Yes	Yes	Yes	Yes	Yes	Yes
Amir-Kabirian 2024	Yes	Yes	Yes	Yes	Yes	Yes	Yes	No
Hadid 2024	Yes	Yes	Yes	Yes	Yes	Yes	Yes	No
Minc 2024	Yes	Yes	Yes	Yes	Yes	Yes	Yes	Yes
Coudray 2017	Yes	Yes	Yes	Yes	Yes	Yes	Yes	Yes

Table S9 - JBI Critical Appraisal - Case Series

Author year	1) Were there clear criteria for inclusion in the case series?	2) Was the condition measured in a standard, reliable way for all participants included in the case series?	3) Were valid methods used for identification of the condition for all participants included in the case series?	4) Did the case series have consecutive inclusion of participants?	5) Did the case series have complete inclusion of participants?	6) Was there clear reporting of the demographics of the participants in the study?	7) Was there clear reporting of clinical information of the participants?	8) Were the outcomes or follow up results of cases clearly reported?	9) Was there clear reporting of the presenting site(s)/clinic(s) demographic information?	10) Was statistical analysis appropriate?
Machado 2018	Yes	Yes	Yes	Yes	Yes	No	Yes	Yes	No	Yes
Schwarze 2020	No	Yes	Yes	Yes	Yes	Yes	Yes	Yes	Yes	Yes
Schwarze 2021	Yes	Yes	Yes	No	Yes	Yes	Yes	Yes	Yes	NA
Stenzel 2013	Yes	Yes	Yes	Yes	Yes	No	Yes	Yes	Yes	Yes
Zhang 2018	yes	Yes	Yes	Yes	Yes	Yes	Yes	Yes	Yes	
Ntoulia 2024	Yes	Yes	Yes	No	Yes	Yes	Yes	Yes	Yes	Yes

Duran 2012	Yes	Yes	Yes	Yes	Yes	Yes	Yes	Yes	Yes	Yes
Nguyen 2025	Yes	Yes	Yes	No	No	Yes	Yes	Yes	No	NA
Senior 2000	Yes	Yes	Yes	Yes	Yes	Yes	Yes	Yes	Yes	Yes
Geyer 2020	Yes	Yes	Yes	No	No	Yes	Yes	Yes	Yes	No
Cvitkovic-Roic 2022	Yes	Yes	Yes	Yes	Yes	Yes	Yes	Yes	No	Yes
Marschner 2021										
D'Amico 2021	Yes	Yes	Yes	No	No	Yes	Yes	Yes	No	Yes

Table S10 - JBI Critical Appraisal - Randomized Controlled Trial

Author year	Was true randomization used for assignment of participants to treatment groups?	Was allocation to treatment groups concealed?	Were treatment groups similar at the baseline?	Were participants blind to treatment assignment?	Were those delivering treatment blind to treatment assignment?	Were outcomes assessors blind to treatment assignment?	Were treatment groups treated identically other than the intervention of interest?	Was follow up complete and if not, were differences between groups in terms of their follow up adequately described and analyzed?	Were participants analyzed in the groups to which they were randomized?	Were outcomes measured in the same way for treatment groups?	Were outcomes measured in a reliable way?	Was appropriate statistical analysis used?	Was the trial design appropriate, and any deviations from the standard RCT design (individual randomization, parallel groups) accounted for in the conduct and analysis of the trial?
Bogdahn 1999	No	No	Yes	Yes	No	No	Yes	no	Yes	Yes	Yes	Yes	No

Bokor 2001	Yes	No	Yes	Yes	No	No	Yes	Yes	Yes	Yes	Yes	Yes	Yes
Lv 2021	Yes	yes	Yes	Yes	yes	yes	Yes	no	Yes	Yes	Yes	Yes	Yes
Madjar 2000	Yes	yes	Yes	Yes	yes	yes	Yes	Yes	Yes	Yes	Yes	Yes	Yes
Miyamoto 2014	Yes	yes	Yes	Yes	yes	yes	Yes	No	Yes	Yes	Yes	Yes	Yes
Nanda 2002	yes	yes	yes	yes	yes	no	yes	no	Yes	Yes	Yes	Yes	Yes
Soman 2000	yes	yes	yes	yes	yes	yes	yes	yes	yes	yes	yes	yes	yes
Kaps 2006	Yes	No	No	No	No	Yes	Yes	Yes	Yes	Yes	Yes	Yes	Yes
Kitzman 2000	Yes	Yes	Yes	Yes	Yes	Yes	Yes	Yes	Yes	Yes	Yes	Yes	Yes
Morel 2000	Yes	No	Yes	No	No	No	Yes	Yes	Yes	Yes	Yes	Yes	No
Kaps 1999	Yes	No	No	No	No	Yes	Yes	Yes	Yes	Yes	Yes	Yes	Yes

Table S11 - JBI Critical Appraisal - Prevalence Study

Author year	1) Was the sample frame appropriate to address the target population	2) Were study participants sampled in an appropriate way?	3) Was the sample size adequate?	4) Were the study subjects and the setting described in detail?	5) Was the data analysis conducted with sufficient coverage of the identified sample?	6) Were valid methods used for the identification of the condition?	7) Was the condition measured in a standard, reliable way for all participants?	8) Was there appropriate statistical analysis?	9) Was the response rate adequate, and if not, was the low response rate managed appropriately?
Kumar 2019	Yes	Yes	Yes	Yes	Yes	Yes	Yes	Yes	Yes
Nucifora 2008	Yes	Yes	Yes	Yes	Yes	Yes	Yes	Yes	Yes
Tang 2017	Yes	Yes	Yes	Yes	Yes	Yes	Yes	Yes	Yes
Torres 2017	Yes	Yes	Yes	Yes	Yes	Yes	Yes	Yes	Yes
Wei 2012	Yes	Yes	Yes	Yes	Yes	Yes	Yes	Yes	Yes
Dietrich	Yes	Yes	Yes	Yes	Yes	Yes	Yes	Yes	No
Piskunovicz 2015	Yes	Yes	Yes	Yes	Yes	Yes	Yes	Yes	Yes
Cvitkovic-Roic 2022	Yes	Yes	Yes	Yes	Yes	Yes	Yes	Yes	Yes
Geleijnse 2009	Yes	Yes	Yes	Yes	Yes	Yes	Yes	Yes	No

Table S12 - JBI Critical Appraisal - Cohort Study

Author year	1) Were the two groups similar and recruited from the same population?	2) Were the exposures measured similarly to assign people to both exposed and unexposed groups?	3) Was the exposure measured in a valid and reliable way?	4) Were confounding factors identified?	5) Were strategies to deal with confounding factors stated?	6) Were the groups/participants free of the outcome at the start of the study (or at the moment of exposure)?	7) Were the outcomes measured in a valid and reliable way?	8) Was the follow up time reported and sufficient to be long enough for outcomes to occur?	9) Was follow up complete, and if not, were the reasons to loss to follow up described and explored?	10) Were strategies to address incomplete follow up utilized?	11) Was appropriate statistical analysis used?
Abdelmoneim 2009	Yes	Yes	Yes	Yes	Yes	Yes	Yes	Yes	Yes	Yes	Yes
Anantharam 2009	Yes	Yes	Yes	Yes	No	Yes	Yes	no	No	No	Yes
Argulian 2020	No	No	Yes	No	No	Yes	Yes	No	Yes	Yes	No
Baracchini 2017	Yes	Yes	Yes	Yes	No	Yes	Yes	Yes	Yes	Yes	Yes
Chou 2019	No	Yes	Yes	No	No	Yes	Yes	Yes	Yes	Yes	No

Cohen 1998	Yes	Yes	Yes	Yes	Yes	Yes	Yes	Yes	No	No	Yes
Coleman 2015	Yes	No	Yes	Yes	No	Yes	Yes	No	No	No	Yes
Dolan 2009	Yes	Yes	Yes	Yes	Yes	Yes	Yes	Yes	No	No	Yes
Fischer 2020	No	Yes	Yes	No	No	Yes	Yes	Yes	Yes	Yes	No
Furtado 2021	Yes	Yes	Yes	Yes	Yes	Yes	Yes	no	No	Yes	Yes
Gaibazzi 2009	Yes	Yes	Yes	No	No	Yes	Yes	Yes	No	No	Yes
Herzog 2008	Yes	Yes	Yes	Yes	No	Yes	Yes	Yes	No	No	Yes
Hu 2019	Yes	Yes	Yes	No	No	Yes	Yes	Yes	Yes	No	Yes
Kusnetzky 2008	Yes	Yes	Yes	Yes	No	Yes	Yes	Yes	No	No	Yes
Kutty 2016	Yes	Yes	Yes	No	No	Yes	Yes	Yes	Yes	Yes	Yes
Mao 2019	Yes	Yes	Yes	Yes	Yes	Yes	Yes	Yes	No	No	Yes
McMahon 2005	Yes	Yes	Yes	Yes	Yes	Yes	Yes	Yes	Yes	Yes	Yes
Papadopoulou 2014	Yes	Yes	Yes	Yes	Yes	Yes	Yes	Yes	Yes	Yes	Yes
Piscaglia 2006	Yes	Yes	Yes	No	No	No	Yes	Yes	No	No	Yes
Platts 2013	Yes	Yes	Yes	No	No	No	Yes	Yes	No	Yes	Yes
Riccabona 2012	Yes	Yes	Yes	No	No	Yes	Yes	Yes	No	No	Yes

Sauer 2017	No	No	Yes	No	No	Yes	Yes	Yes	Yes	Yes	Yes
Seelbach 2022	Yes	Yes	Yes	No	No	Yes	Yes	Yes	Yes	No	Yes
Senior 2000	Yes	Yes	Yes	Yes	No	Yes	Yes	Yes	Yes	No	Yes
Shaikh 2008	Yes	Yes	Yes	Yes	y	Yes	Yes	No	Yes	No	Yes
Shang 2023	Yes	Yes	Yes	Yes	No	Yes	Yes	Yes	No	No	Yes
Suma 2013	Yes	Yes	Yes	Yes	Yes	Yes	Yes	Yes	Yes	Yes	Yes
Suma 2016	Yes	Yes	Yes	No	No	Yes	Yes	no	No	No	Yes
Timperley 2005	Yes	Yes	Yes	Yes	Yes	Yes	Yes	Yes	No	No	Yes
Wei 2008	Yes	Yes	Yes	Yes	Yes	Yes	Yes	no	No	No	Yes
Wei 2014	Yes	Yes	Yes	Yes	No	Yes	Yes	Yes	Yes	No	Yes
Weiss 2012	Yes	Yes	Yes	Yes	Yes	Yes	Yes	Yes	No	No	Yes
Wever-Pinzon 2012	Yes	Yes	Yes	Yes	Yes	Yes	Yes	Yes	Yes	Yes	Yes
Wu 2023	Yes	Yes	Yes	Yes	No	Yes	Yes	Yes	Yes	No	Yes
Li 2023	No	No	Yes	Yes	Yes	Yes	No	No	No	No	Yes
Li 2024	No	No	Yes	Yes	No	Yes	Yes	Yes	No	No	Yes
Ntoulia 2018	Yes	Yes	Yes	Yes	Yes	Yes	Yes	Yes	Yes	No	Yes

Table S13 - JBI Critical Appraisal - Diagnostic Test Accuracy Study

Author year	Was a consecutive or random sample of patients enrolled?	Was a case-control design avoided?	Did the study avoid inappropriate exclusions?	Were the index test results interpreted without knowledge of the results of the reference standard?	If a threshold was used, was it pre-specified?	Is the reference standard likely to correctly classify the target condition?	Were the reference standard results interpreted without knowledge of the results of the index test?	Was there an appropriate interval between index test and reference standard?	Did all patients receive the same reference standard?	Were all patients included in the analysis?
Papadopoulou 2009	Yes	Yes	Yes	Yes	Yes	Yes	No	Yes	Yes	Yes
Kis 2010	Yes	Yes	Yes	Yes	Yes	Yes	Yes	Yes	Yes	Yes
Kljucevsek 2011	Yes	Yes	Yes	No	Yes	Yes	No	Yes	Yes	Yes
Kim 2021	Yes	Yes	Yes	Yes	Yes	Yes	Yes	Yes	Yes	Yes
Faizah 2015	Yes	Yes	Yes	Yes	Yes	Yes	Yes	Yes	Yes	Yes
Knieling 2016	No	Yes	No	No	No	Yes	No	No	No	No

Table S14 - JBI Critical Appraisal - Cross Sectional Study

Author year	1.Were the criteria for inclusion in the sample clearly defined?	2. Were the study subjects and the setting described in detail?	3. Was the exposure measured in a valid and reliable way?	4. Were objective, standard criteria used for measurement of the condition?	5. Were confounding factors identified?	6. Were strategies to deal with confounding factors stated?	7. Were the outcomes measured in a valid and reliable way?	8. Was appropriate statistical analysis used?
Gupta 2015	No	Yes	Yes	Yes	No	No	Yes	Yes
Wong 2014	Yes	Yes	Yes	Yes	No	No	Yes	Yes
Siomou 2020	Yes	Yes	Yes	Yes	No	No	Yes	Yes
Kuzmanovska 2017	No	Yes	Yes	Yes	No	No	Yes	No
Riggs 2024	Yes	Yes	Yes	Yes	No	No	Yes	No
Fang 2004	Yes	Yes	Yes	Yes	No	No	No	No

Figures

Figure S1 - Forest Plot Severe Adverse Events – Adults

Study	N events	Total patients	Proportion [95% CI]
Wei 2008 - stress echocardiography - Definity	10	66164	0.00015 [0.00008, 0.00028]
Li 2023 - abdominal ceus - SonoVue	7	49100	0.00014 [0.00007, 0.00030]
Li 2024 - abdominal ceus - SonoVue	5	83778	0.00006 [0.00002, 0.00014]
Shang 2023 - abdominal ceus - SonoVue	4	463434	0.00001 [0.00000, 0.00002]
Herzog 2008 - - Optison or Definity	4	16025	0.00025 [0.00009, 0.00066]
Herzog 2008 - - Definity	4	12974	0.00031 [0.00012, 0.00082]
Hu 2019 - abdominal ceus - SonoVue	3	34478	0.00009 [0.00003, 0.00027]
Geleijnse 2009 - - SonoVue	3	274	0.01095 [0.00354, 0.03339]
Shaikh 2008 - - Optison or Definity	2	2914	0.00069 [0.00017, 0.00274]
Lv 2021 - abdominal ceus - Sonazoid	2	218	0.00917 [0.00230, 0.03592]
Lv 2021 - abdominal ceus - SonoVue	2	206	0.00971 [0.00243, 0.03797]
Piscaglia 2006 - abdominal ceus - SonoVue	2	23188	0.00009 [0.00002, 0.00034]
Fischer 2020 - - SonoVue	1	764	0.00131 [0.00018, 0.00923]
Kaps 2001 - - SonoVue	0	40	0.01220 [0.00076, 0.16720]
Kitzman 2000 - - Definity	0	169	0.00294 [0.00018, 0.04521]
Chou 2019 - abdominal ceus - Sonazoid	0	54	0.00909 [0.00057, 0.12933]
Madjar 2000 - breast ceus - SonoVue	0	40	0.01220 [0.00076, 0.16720]
Miyamoto 2014 - breast ceus - Sonazoid	0	117	0.00424 [0.00026, 0.06405]
Wu 2023 - - Definity	0	55	0.00893 [0.00056, 0.12728]
Fang 2004 - - SonoVue	0	90	0.00549 [0.00034, 0.08173]
Nguyen 2025 - - SonoVue	0	15	0.03125 [0.00193, 0.35028]
Herzog 2008 - stress echocardiography - Optison	0	3051	0.00016 [0.00001, 0.00261]
Kumar 2019 - stress echocardiography - Definity	0	3306	0.00015 [0.00001, 0.00241]
Kumar 2019 - stress echocardiography - Lumason	0	2137	0.00023 [0.00001, 0.00373]
Kumar 2019 - stress echocardiography - Optison	0	78	0.00633 [0.00039, 0.09316]
Platts 2013 - stress echocardiography - Definity	0	5576	0.00009 [0.00001, 0.00143]
Suma 2016 - stress echocardiography - Definity	0	179	0.00278 [0.00017, 0.04279]
Suma 2016 - stress echocardiography - Optison	0	40	0.01220 [0.00076, 0.16720]
Wei 2008 - stress echocardiography - Optison	0	12219	0.00004 [0.00000, 0.00065]
Wei 2014 - stress echocardiography - Optison	0	1039	0.00048 [0.00003, 0.00764]
Weiss 2012 - stress echocardiography - Definity	0	1053	0.00047 [0.00003, 0.00754]
Cohen 1998 - rest echocardiography - Optison	0	198	0.00251 [0.00016, 0.03884]
Dolan 2009 - rest echocardiography - Optison or Definity	0	23659	0.00002 [0.00000, 0.00034]
Kusnetzky 2008 - rest echocardiography - Definity	0	6196	0.00008 [0.00001, 0.00129]
Nanda 2002 - rest echocardiography - SonoVue	0	138	0.00360 [0.00022, 0.05483]
Nucifora 2008 - rest echocardiography - Luminity	0	115	0.00431 [0.00027, 0.06509]
Senior 2000 - rest echocardiography - SonoVue	0	218	0.00228 [0.00014, 0.03540]
Soman 2000 - rest echocardiography - SonoVue	0	13	0.03571 [0.00220, 0.38387]
Wei 2012 - rest echocardiography - Definity	0	32	0.01515 [0.00094, 0.20079]
Dolan 2009 - stress echocardiography - Definity	0	5947	0.00008 [0.00001, 0.00134]
Dolan 2009 - stress echocardiography - Optison	0	12802	0.00004 [0.00000, 0.00062]
Furtado 2021 - stress echocardiography - SonoVue	0	1062	0.00047 [0.00003, 0.00747]
Wever-Pinzon 2012 - stress echocardiography - Definity	0	1513	0.00033 [0.00002, 0.00526]
Timperley 2005 - stress echocardiography - SonoVue	0	299	0.00167 [0.00010, 0.02606]
Timperley 2005 - stress echocardiography - Optison	0	120	0.00413 [0.00026, 0.06255]
Bogdahn 1999 - transcranial doppler - SonoVue	0	10	0.04545 [0.00278, 0.44831]
Abdelmoneim 2009 - other - Optison or Definity	0	10792	0.00005 [0.00000, 0.00074]
Anantharam 2009 - - Luminity	0	461	0.00108 [0.00007, 0.01705]
Anantharam 2009 - - SonoVue or Luminity	0	689	0.00072 [0.00005, 0.01147]
Argulian 2020 - - Optison	0	33	0.01471 [0.00091, 0.19588]
Baracchini 2017 - - SonoVue	0	92	0.00538 [0.00034, 0.08009]
Bokor 2001 - - SonoVue	0	12	0.03846 [0.00236, 0.40319]
Random-Effects Model			0.00006 [0.00002, 0.00018]

Random-Effects Model: $\tau^2 = 4.66$; $I^2 = 85.5\%$

Test for overall effect: Z = -16.67 (<0.001)

Proportion (logit back-transformed)

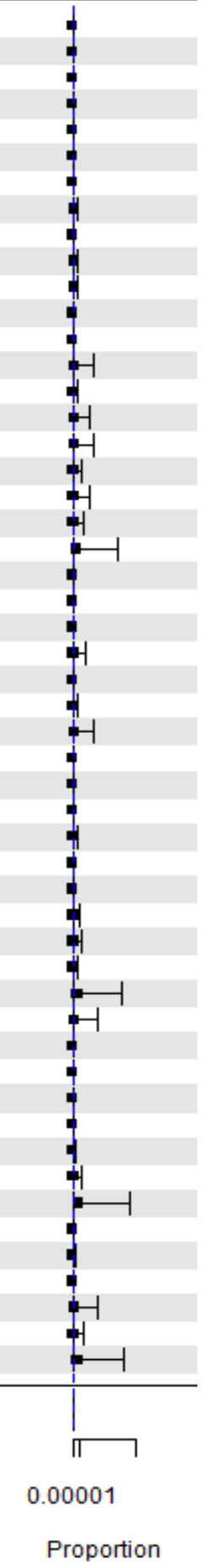

Figure S2 - Forest Plot Severe Adverse Events - Children IV

Study	N events	Total patients	Proportion [95% CI]
dietrich 2021 - abdominal ceus - sonovue	2	1676	0.00119 [0.00030, 0.00476]
piskunovicz 2015 - abdominal ceus - sonovue	1	137	0.00730 [0.00103, 0.04995]
coleman 2014 - abdominal ceus - optison	0	32	0.01515 [0.00094, 0.20079]
mao 2019 - abdominal ceus - sonovue	0	312	0.00160 [0.00010, 0.02500]
seelbach 2022 - - sonovue	0	30	0.01613 [0.00100, 0.21141]
stenzel 2013 - abdominal ceus - sonovue	0	37	0.01316 [0.00082, 0.17839]
torres 2017 - abdominal ceus - sonovue	0	173	0.00287 [0.00018, 0.04421]
knieling 2016 - abdominal ceus - sonovue	0	40	0.01220 [0.00076, 0.16720]
seelbach 2022 - cevus - sonovue	0	56	0.00877 [0.00055, 0.12528]
ntoulia 2018 - cevus - optison	0	30	0.01613 [0.00100, 0.21141]
sauer 2017 - cevus - sonovue	0	531	0.00094 [0.00006, 0.01484]
cvitkovic-roic 2022 - cevus - sonovue	0	5153	0.00010 [0.00001, 0.00155]
papadopoulou 2014 - cevus - sonovue	0	1010	0.00049 [0.00003, 0.00785]
papadopoulou 2009 - cevus - sonovue	0	228	0.00218 [0.00014, 0.03390]
duran 2012 - cevus - sonovue	0	295	0.00169 [0.00011, 0.02640]
kis 2010 - cevus - sonovue	0	183	0.00272 [0.00017, 0.04190]
kljucevsek 2011 - cevus - sonovue	0	66	0.00746 [0.00047, 0.10831]
siomou 2020 - cevus - sonovue	0	60	0.00820 [0.00051, 0.11789]
kim 2021 - cevus - sonovue	0	32	0.01515 [0.00094, 0.20079]
kuzmanovska 2017 - cevus - sonovue	0	31	0.01563 [0.00097, 0.20597]
wong 2014 - cevus - sonovue	0	31	0.01563 [0.00097, 0.20597]
faizah 2015 - cevus - sonovue	0	27	0.01786 [0.00111, 0.22962]
marschner 2021 - cevus - sonazoid	0	49	0.01000 [0.00062, 0.14072]
zhang 2018 - cevus - sonovue	0	90	0.00549 [0.00034, 0.08173]
ntoulia 2024 - - optison	0	12	0.03846 [0.00236, 0.40319]
ntoulia 2024 - - sonovue	0	4	0.10000 [0.00595, 0.67360]
riggs 2024 - - sonovue	0	38	0.01282 [0.00080, 0.17450]
kutty 2016 - stress echocardiography - definity	0	113	0.00439 [0.00027, 0.06617]
mcmahon 2005 - rest echocardiography - optison	0	20	0.02381 [0.00147, 0.28738]
Random-Effects Model			0.00016 [0.00001, 0.00217]

Random-Effects Model: $\tau^2 = 1.8$; $I^2 = 50.1\%$

Test for overall effect: Z = -6.61 (<0.001)

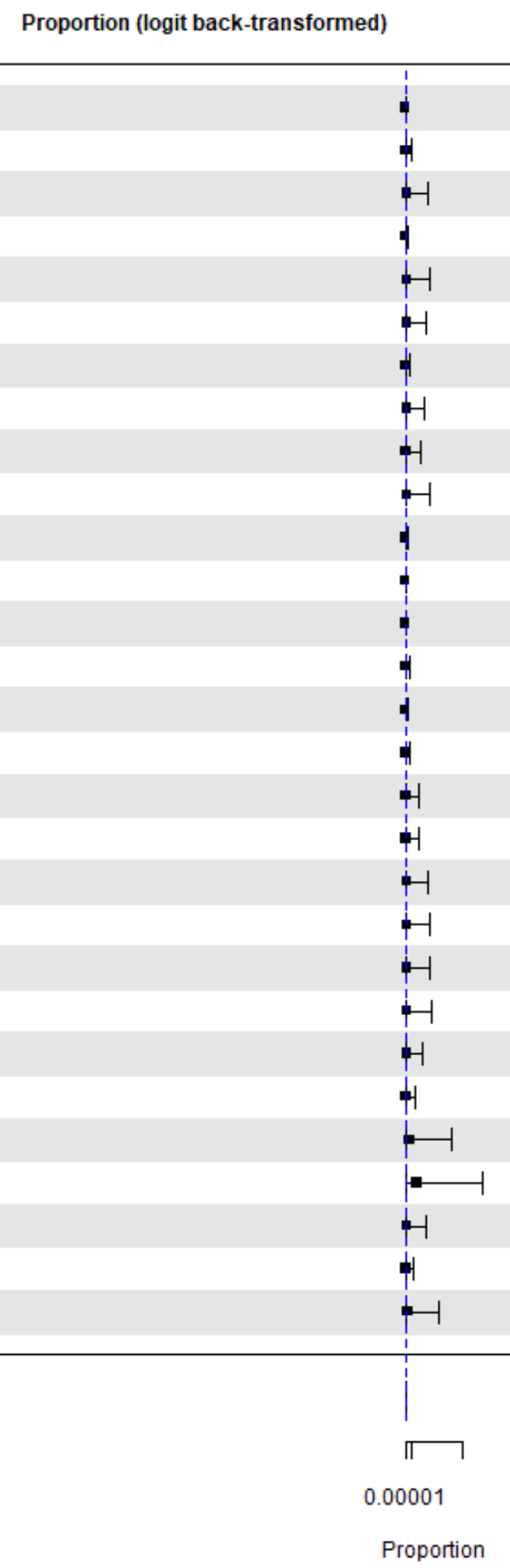

Figure S3 - Forest Plot Non-Severe Adverse Events – Adults

Study	N events	Total patients	Proportion [95% CI]
shang 2023 - abdominal ceus - sonovue	136	463434	0.00029 [0.00025, 0.00035]
abdelmoneim 2009 - other - optison or definity	41	10792	0.00380 [0.00280, 0.00516]
weiss 2012 - stress echocardiography - definity	37	1053	0.03514 [0.02556, 0.04812]
hu 2019 - abdominal ceus - sonovue	36	34478	0.00104 [0.00075, 0.00145]
li 2023 - abdominal ceus - sonovue	36	49100	0.00073 [0.00053, 0.00102]
piscaglia 2006 - abdominal ceus - sonovue	22	23188	0.00095 [0.00062, 0.00144]
platts 2013 - stress echocardiography - definity	16	5576	0.00287 [0.00176, 0.00468]
li 2024 - abdominal ceus - sonovue	15	83778	0.00018 [0.00011, 0.00030]
kumar 2019 - stress echocardiography - definity	13	3306	0.00393 [0.00228, 0.00676]
herzog 2008 - - definity.1	12	5231	0.00229 [0.00130, 0.00403]
nanda 2002 - rest echocardiography - sonovue	12	138	0.08696 [0.05004, 0.14688]
senior 2000 - rest echocardiography - sonovue	12	218	0.05505 [0.03152, 0.09441]
kitzman 2000 - - definity	9	169	0.05325 [0.02794, 0.09917]
cohen 1998 - rest echocardiography - optison	9	198	0.04545 [0.02382, 0.08503]
kaps 1999 - - sonovue	8	12	0.66667 [0.37588, 0.86914]
fang 2004 - - sonovue	6	90	0.06667 [0.03026, 0.14054]
baracchini 2017 - - sonovue	6	92	0.06522 [0.02959, 0.13764]
nucifora 2008 - rest echocardiography - definity	5	115	0.04348 [0.01821, 0.10022]
geleijnse 2009 - - sonovue	4	274	0.01460 [0.00549, 0.03824]
herzog 2008 - - definity.2	4	6959	0.00057 [0.00022, 0.00153]
furtado 2021 - stress echocardiography - sonovue	4	1062	0.00377 [0.00141, 0.00999]
fischer 2020 - - sonovue	3	764	0.00393 [0.00127, 0.01210]
wei 2012 - rest echocardiography - definity	3	32	0.09375 [0.03055, 0.25350]
madjar 2000 - breast ceus - sonovue	2	40	0.05000 [0.01254, 0.17909]
wei 2014 - stress echocardiography - optison	2	1039	0.00192 [0.00048, 0.00766]
bokor 2001 - - sonovue.1	2	44	0.04545 [0.01140, 0.16438]
bokor 2001 - - sonovue.2	2	12	0.16667 [0.04198, 0.47720]
kumar 2019 - stress echocardiography - sonovue	1	2137	0.00047 [0.00007, 0.00331]
anantharam 2009 - - definity	1	461	0.00217 [0.00031, 0.01523]
kaps 2001 - - sonovue	0	40	0.01220 [0.00076, 0.16720]
morel 2000 - - sonovue	0	12	0.03846 [0.00236, 0.40319]
shaikh 2008 - - optison or definity	0	2914	0.00017 [0.00001, 0.00274]
chou 2019 - abdominal ceus - sonazoid	0	54	0.00909 [0.00057, 0.12933]
lv 2021 - abdominal ceus - sonazoid	0	218	0.00228 [0.00014, 0.03540]
lv 2021 - abdominal ceus - sonovue	0	206	0.00242 [0.00015, 0.03739]
miyamoto 2014 - breast ceus - sonazoid	0	117	0.00424 [0.00026, 0.06405]
wu 2023 - - definity.1	0	55	0.00893 [0.00056, 0.12728]
wu 2023 - - definity.2	0	43	0.01136 [0.00071, 0.15733]
nguyen 2025 - - sonovue	0	15	0.03125 [0.00193, 0.35028]
herzog 2008 - stress echocardiography - optison	0	3051	0.00016 [0.00001, 0.00261]
herzog 2008 - - definity.3	0	784	0.00064 [0.00004, 0.01010]
kumar 2019 - stress echocardiography - optison	0	78	0.00633 [0.00039, 0.09316]
suma 2016 - stress echocardiography - definity	0	179	0.00278 [0.00017, 0.04279]
suma 2016 - stress echocardiography - optison	0	40	0.01220 [0.00076, 0.16720]
wei 2008 - stress echocardiography - optison	0	12219	0.00004 [0.00000, 0.00065]
dolan 2009 - rest echocardiography - optison or definity	0	23659	0.00002 [0.00000, 0.00034]
kusnetzky 2008 - rest echocardiography - definity	0	6196	0.00008 [0.00001, 0.00129]
soman 2000 - rest echocardiography - sonovue	0	13	0.03571 [0.00220, 0.38387]
dolan 2009 - stress echocardiography - definity	0	5947	0.00008 [0.00001, 0.00134]
dolan 2009 - stress echocardiography - optison	0	12802	0.00004 [0.00000, 0.00062]
wever-pinzon 2012 - stress echocardiography - definity	0	1513	0.00033 [0.00002, 0.00526]
timperley 2005 - stress echocardiography - sonovue	0	299	0.00167 [0.00010, 0.02606]
bogdahn 1999 - transcranial doppler - sonovue	0	10	0.04545 [0.00278, 0.44831]
anantharam 2009 - - sonovue or definity	0	689	0.00072 [0.00005, 0.01147]
argulian 2020 - - optison	0	33	0.01471 [0.00091, 0.19588]
Random-Effects Model			0.00109 [0.00038, 0.00311]

Random-Effects Model: $\tau^2 = 9.94$; $I^2 = 98.7\%$

Test for overall effect: Z = -12.74 (<0.001)

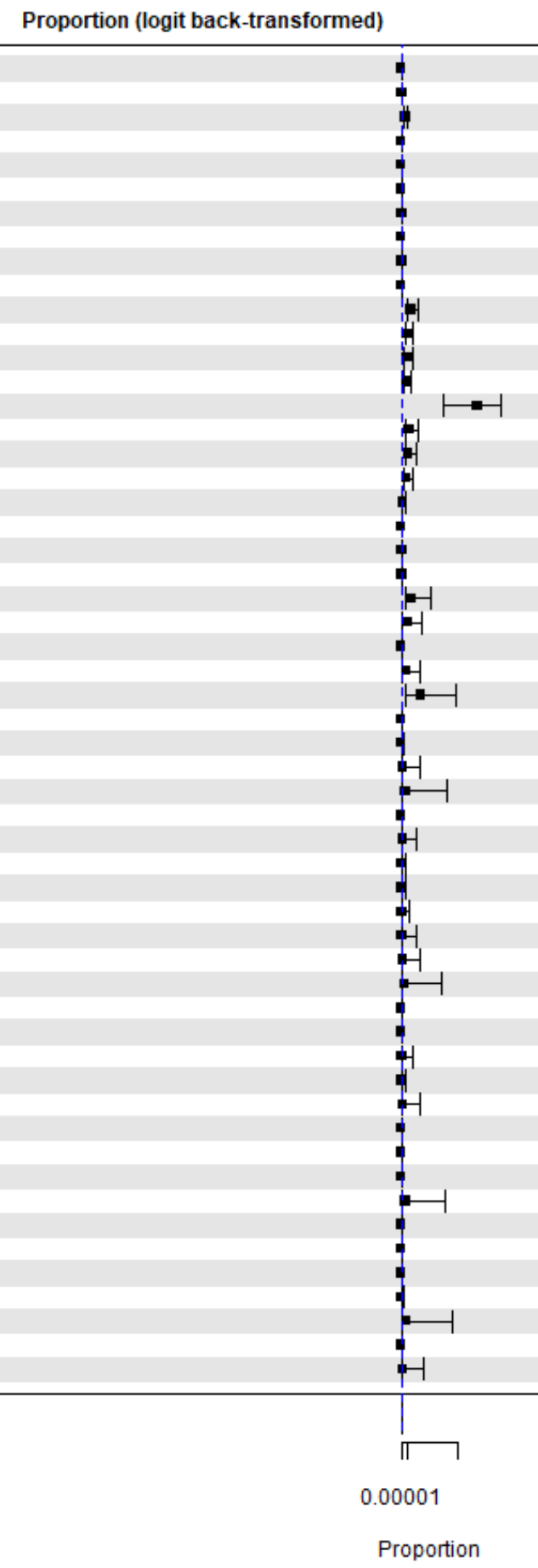

Figure S4 - Forest Plot Non Severe Adverse Events – Children IV

Study	N events	Total patients	Proportion [95% CI]
dietrich 2021 - abdominal ceus - sonovue	13	1676	0.00776 [0.00451, 0.01331]
mao 2019 - abdominal ceus - sonovue	6	312	0.01923 [0.00866, 0.04213]
coleman 2014 - abdominal ceus - optison	3	32	0.09375 [0.03055, 0.25350]
mcmahon 2005 - rest echocardiography - optison	3	20	0.15000 [0.04917, 0.37584]
knieling 2016 - abdominal ceus - sonovue	2	40	0.05000 [0.01254, 0.17909]
seelbach 2022 - - sonovue	1	30	0.03333 [0.00468, 0.20200]
stenzel 2013 - abdominal ceus - sonovue	1	37	0.02703 [0.00379, 0.16847]
seelbach 2022 - cevus - sonovue	1	56	0.01786 [0.00251, 0.11613]
torres 2017 - abdominal ceus - sonovue	0	173	0.00287 [0.00018, 0.04421]
piskunovicz 2015 - abdominal ceus - sonovue	0	137	0.00362 [0.00023, 0.05520]
ntoulia 2018 - cevus - optison	0	30	0.01613 [0.00100, 0.21141]
sauer 2017 - cevus - sonovue	0	531	0.00094 [0.00006, 0.01484]
cvitkovic-roic 2022 - cevus - sonovue	0	5153	0.00010 [0.00001, 0.00155]
papadopoulou 2014 - cevus - sonovue	0	1010	0.00049 [0.00003, 0.00785]
papadopoulou 2009 - cevus - sonovue	0	228	0.00218 [0.00014, 0.03390]
duran 2012 - cevus - sonovue	0	295	0.00169 [0.00011, 0.02640]
kis 2010 - cevus - sonovue	0	183	0.00272 [0.00017, 0.04190]
kljucevsek 2011 - cevus - sonovue	0	66	0.00746 [0.00047, 0.10831]
siomou 2020 - cevus - sonovue	0	60	0.00820 [0.00051, 0.11789]
kim 2021 - cevus - sonovue	0	32	0.01515 [0.00094, 0.20079]
kuzmanovska 2017 - cevus - sonovue	0	31	0.01563 [0.00097, 0.20597]
wong 2014 - cevus - sonovue	0	31	0.01563 [0.00097, 0.20597]
faizah 2015 - cevus - sonovue	0	27	0.01786 [0.00111, 0.22962]
marschner 2021 - cevus - sonazoid	0	49	0.01000 [0.00062, 0.14072]
zhang 2018 - cevus - sonovue	0	90	0.00549 [0.00034, 0.08173]
ntoulia 2024 - - optison	0	12	0.03846 [0.00236, 0.40319]
ntoulia 2024 - - sonovue	0	4	0.10000 [0.00595, 0.67360]
riggs 2024 - - sonovue	0	38	0.01282 [0.00080, 0.17450]
kutty 2016 - stress echocardiography - definity	0	113	0.00439 [0.00027, 0.06617]
Random-Effects Model			0.00081 [0.00011, 0.00590]

Random-Effects Model: $\tau^2 = 8.21$; $I^2 = 90.8\%$

Test for overall effect: Z = -7.02 (<0.001)

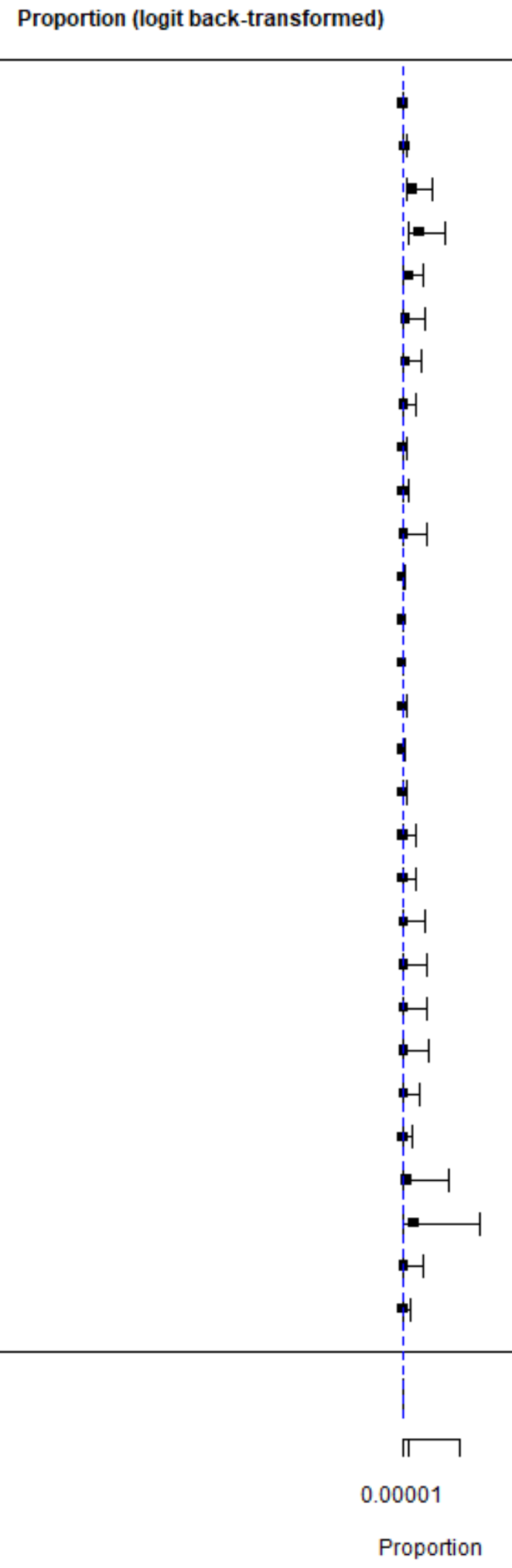

Figure S5 - Forest Plot Non Severe Adverse Events – Children Endocavitary

Study	N events	Total patients	Proportion [95% CI]
seelbach 2022 - cevus - sonovue	1	56	0.01786 [0.00251, 0.11613]
ntoulia 2018 - cevus - optison	0	30	0.01613 [0.00100, 0.21141]
sauer 2017 - cevus - sonovue	0	531	0.00094 [0.00006, 0.01484]
cvitkovic-roic 2022 - cevus - sonovue	0	5153	0.00010 [0.00001, 0.00155]
papadopoulou 2014 - cevus - sonovue	0	1010	0.00049 [0.00003, 0.00785]
papadopoulou 2009 - cevus - sonovue	0	228	0.00218 [0.00014, 0.03390]
duran 2012 - cevus - sonovue	0	295	0.00169 [0.00011, 0.02640]
kis 2010 - cevus - sonovue	0	183	0.00272 [0.00017, 0.04190]
kljucevsek 2011 - cevus - sonovue	0	66	0.00746 [0.00047, 0.10831]
siomou 2020 - cevus - sonovue	0	60	0.00820 [0.00051, 0.11789]
kim 2021 - cevus - sonovue	0	32	0.01515 [0.00094, 0.20079]
kuzmanovska 2017 - cevus - sonovue	0	31	0.01563 [0.00097, 0.20597]
wong 2014 - cevus - sonovue	0	31	0.01563 [0.00097, 0.20597]
faizah 2015 - cevus - sonovue	0	27	0.01786 [0.00111, 0.22962]
marschner 2021 - cevus - sonazoid	0	49	0.01000 [0.00062, 0.14072]
zhang 2018 - cevus - sonovue	0	90	0.00549 [0.00034, 0.08173]
Random-Effects Model			0.00001 [0.00000, 0.12340]

Random-Effects Model: $\tau^2 = 12.28$; $I^2 = 86.6\%$

Test for overall effect: Z = -2.36 (0.018)

Proportion (logit back-transformed)

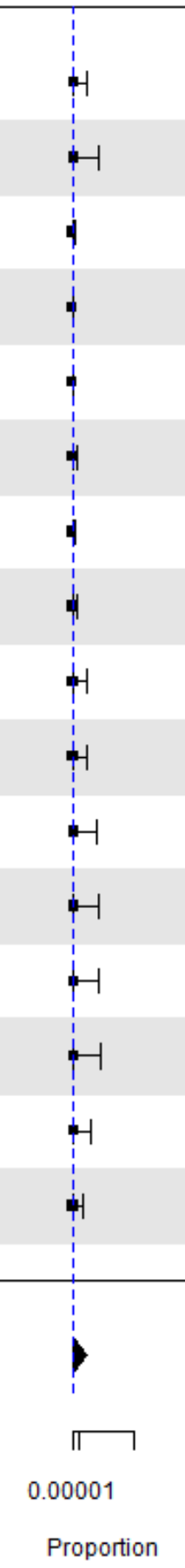

Figure S6 - Forest Plot Delayed Adverse Events – Adults

Study	N events	Total patients	Proportion [95% CI]
piscaglia 2006 - abdominal ceus - sonovue	5	23188	0.000216 [0.000090, 0.000518]
miyamoto 2014 - breast ceus - sonazoid	4	117	0.034188 [0.012891, 0.087550]
kitzman 2000 - - definity	2	169	0.011834 [0.002962, 0.046059]
hu 2019 - abdominal ceus - sonovue	1	34478	0.000029 [0.000004, 0.000206]
furtado 2021 - stress echocardiography - sonovue	1	1062	0.000942 [0.000133, 0.006653]
kaps 2001 - - sonovue	0	40	0.012195 [0.000759, 0.167203]
morel 2000 - - sonovue	0	12	0.038462 [0.002363, 0.403194]
kaps 1999 - - sonovue	0	12	0.038462 [0.002363, 0.403194]
chou 2019 - abdominal ceus - sonazoid	0	54	0.009091 [0.000566, 0.129335]
lv 2021 - abdominal ceus - sonazoid	0	218	0.002283 [0.000143, 0.035402]
lv 2021 - abdominal ceus - sonovue	0	206	0.002415 [0.000151, 0.037389]
madjar 2000 - breast ceus - sonovue	0	40	0.012195 [0.000759, 0.167203]
geleijnse 2009 - - sonovue	0	274	0.001818 [0.000114, 0.028367]
shang 2023 - abdominal ceus - sonovue	0	463434	0.000001 [0.000000, 0.000017]
li 2023 - abdominal ceus - sonovue	0	49100	0.000010 [0.000001, 0.000163]
li 2024 - abdominal ceus - sonovue	0	83778	0.000006 [0.000000, 0.000095]
fischer 2020 - - sonovue	0	764	0.000654 [0.000041, 0.010357]
wu 2023 - - definity.1	0	55	0.008929 [0.000556, 0.127276]
wu 2023 - - definity.2	0	43	0.011364 [0.000707, 0.157332]
fang 2004 - - sonovue	0	90	0.005495 [0.000343, 0.081732]
nguyen 2025 - - sonovue	0	15	0.031250 [0.001926, 0.350276]
herzog 2008 - stress echocardiography - optison	0	3051	0.000164 [0.000010, 0.002613]
herzog 2008 - - definity	0	6959	0.000072 [0.000004, 0.001147]
kumar 2019 - stress echocardiography - definity	0	3306	0.000151 [0.000009, 0.002412]
kumar 2019 - stress echocardiography - sonovue	0	2137	0.000234 [0.000015, 0.003727]
platts 2013 - stress echocardiography - definity	0	5576	0.000090 [0.000006, 0.001432]
suma 2016 - stress echocardiography - definity	0	179	0.002778 [0.000174, 0.042793]
suma 2016 - stress echocardiography - optison	0	40	0.012195 [0.000759, 0.167203]
wei 2008 - stress echocardiography - optison	0	12219	0.000041 [0.000003, 0.000654]
wei 2014 - stress echocardiography - optison	0	1039	0.000481 [0.000030, 0.007636]
weiss 2012 - stress echocardiography - definity	0	1053	0.000474 [0.000030, 0.007536]
cohen 1998 - rest echocardiography - optison	0	198	0.002513 [0.000157, 0.038842]
dolan 2009 - rest echocardiography - optison or definity	0	23659	0.000021 [0.000001, 0.000338]
kusnetzky 2008 - rest echocardiography - definity	0	6196	0.000081 [0.000005, 0.001289]
nanda 2002 - rest echocardiography - sonovue	0	138	0.003597 [0.000225, 0.054826]
nucifora 2008 - rest echocardiography - definity	0	115	0.004310 [0.000269, 0.065094]
senior 2000 - rest echocardiography - sonovue	0	218	0.002283 [0.000143, 0.035402]
soman 2000 - rest echocardiography - sonovue	0	13	0.035714 [0.002197, 0.383867]
wei 2012 - rest echocardiography - definity	0	32	0.015152 [0.000941, 0.200794]
dolan 2009 - stress echocardiography - definity	0	5947	0.000084 [0.000005, 0.001342]
dolan 2009 - stress echocardiography - optison	0	12802	0.000039 [0.000002, 0.000624]
wever-pinzon 2012 - stress echocardiography - definity	0	1513	0.000330 [0.000021, 0.005256]
timperley 2005 - stress echocardiography - sonovue	0	299	0.001667 [0.000104, 0.026055]
timperley 2005 - stress echocardiography - optison	0	120	0.004132 [0.000258, 0.062547]
bogdahn 1999 - transcranial doppler - sonovue	0	10	0.045455 [0.002783, 0.448314]
abdelmoneim 2009 - other - optison or definity	0	10792	0.000046 [0.000003, 0.000740]
anantharam 2009 - - definity	0	461	0.001082 [0.000068, 0.017051]
anantharam 2009 - - sonovue or definity	0	689	0.000725 [0.000045, 0.011472]
argulian 2020 - - optison	0	33	0.014706 [0.000914, 0.195875]
baracchini 2017 - - sonovue	0	92	0.005376 [0.000335, 0.080094]
bokor 2001 - - sonovue.1	0	44	0.011111 [0.000691, 0.154296]
bokor 2001 - - sonovue.2	0	12	0.038462 [0.002363, 0.403194]
Random-Effects Model			0.000001 [0.000000, 0.000091]

Random-Effects Model: $\tau^2 = 19.6$; $I^2 = 93\%$

Test for overall effect: Z = -5.83 (<0.001)

Proportion (logit back-transformed)

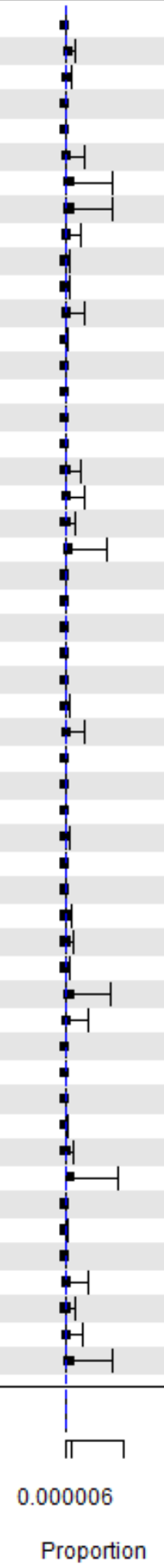

Figure S7 - Forest Plot Delayed Adverse Events – Endocavitary

Study	N events	Total patients	Proportion [95% CI]	Proportion (logit back-transformed)
sauer 2017 - cevus - sonovue	3	531	0.00565 [0.00182, 0.01737]	
seelbach 2022 - cevus - sonovue	0	56	0.00877 [0.00055, 0.12528]	
ntoulia 2018 - cevus - optison	0	30	0.01613 [0.00100, 0.21141]	
cvitkovic-roic 2022 - cevus - sonovue	0	5153	0.00010 [0.00001, 0.00155]	
papadopoulou 2014 - cevus - sonovue	0	1010	0.00049 [0.00003, 0.00785]	
papadopoulou 2009 - cevus - sonovue	0	228	0.00218 [0.00014, 0.03390]	
duran 2012 - cevus - sonovue	0	295	0.00169 [0.00011, 0.02640]	
kis 2010 - cevus - sonovue	0	183	0.00272 [0.00017, 0.04190]	
kljucevsek 2011 - cevus - sonovue	0	66	0.00746 [0.00047, 0.10831]	
siomou 2020 - cevus - sonovue	0	60	0.00820 [0.00051, 0.11789]	
kim 2021 - cevus - sonovue	0	32	0.01515 [0.00094, 0.20079]	
kuzmanovska 2017 - cevus - sonovue	0	31	0.01563 [0.00097, 0.20597]	
wong 2014 - cevus - sonovue	0	31	0.01563 [0.00097, 0.20597]	
faizah 2015 - cevus - sonovue	0	27	0.01786 [0.00111, 0.22962]	
marschner 2021 - cevus - sonazoid	0	49	0.01000 [0.00062, 0.14072]	
zhang 2018 - cevus - sonovue	0	90	0.00549 [0.00034, 0.08173]	
Random-Effects Model			0.00002 [0.00000, 0.02593]	

Random-Effects Model: $\tau^2 = 8.91$; $I^2 = 84.5\%$

Test for overall effect: Z = -2.97 (0.003)

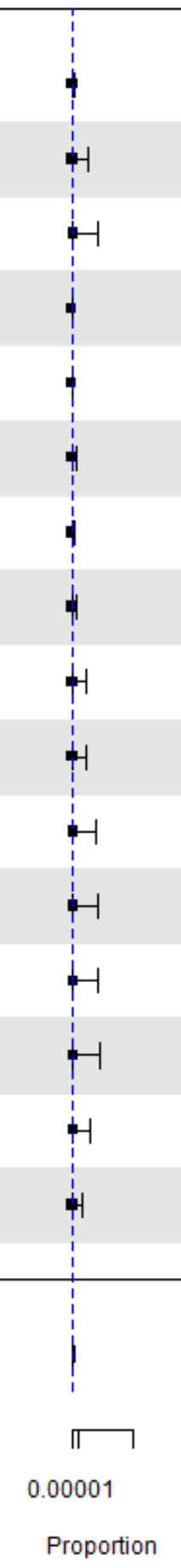

Figure S8 - Forest Plot Severe Adverse Events Cardiologic – Adults

Study	N events	Total patients	Proportion [95% CI]
Kitzman 2000 - - Definity	0	169	0.00294 [0.00018, 0.04521]
Shaikh 2008 - - Optison or Definity	2	2914	0.00069 [0.00017, 0.00274]
Geleijnse 2009 - - SonoVue	3	274	0.01095 [0.00354, 0.03339]
Fang 2004 - - SonoVue	0	90	0.00549 [0.00034, 0.08173]
Herzog 2008 - stress echocardiography - Optison	0	3051	0.00016 [0.00001, 0.00261]
Herzog 2008 - - Optison or Definity	4	16025	0.00025 [0.00009, 0.00066]
Herzog 2008 - - Definity	4	12974	0.00031 [0.00012, 0.00082]
Kumar 2019 - stress echocardiography - Definity	0	3306	0.00015 [0.00001, 0.00241]
Kumar 2019 - stress echocardiography - Lumason	0	2137	0.00023 [0.00001, 0.00373]
Kumar 2019 - stress echocardiography - Optison	0	78	0.00633 [0.00039, 0.09316]
Platts 2013 - stress echocardiography - Definity	0	5576	0.00009 [0.00001, 0.00143]
Suma 2016 - stress echocardiography - Definity	0	179	0.00278 [0.00017, 0.04279]
Suma 2016 - stress echocardiography - Optison	0	40	0.01220 [0.00076, 0.16720]
Wei 2008 - stress echocardiography - Definity	10	66164	0.00015 [0.00008, 0.00028]
Wei 2008 - stress echocardiography - Optison	0	12219	0.00004 [0.00000, 0.00065]
Wei 2014 - stress echocardiography - Optison	0	1039	0.00048 [0.00003, 0.00764]
Weiss 2012 - stress echocardiography - Definity	0	1053	0.00047 [0.00003, 0.00754]
Cohen 1998 - rest echocardiography - Optison	0	198	0.00251 [0.00016, 0.03884]
Dolan 2009 - rest echocardiography - Optison or Definity	0	23659	0.00002 [0.00000, 0.00034]
Kusnetzky 2008 - rest echocardiography - Definity	0	6196	0.00008 [0.00001, 0.00129]
Nanda 2002 - rest echocardiography - SonoVue	0	138	0.00360 [0.00022, 0.05483]
Nucifora 2008 - rest echocardiography - Luminity	0	115	0.00431 [0.00027, 0.06509]
Senior 2000 - rest echocardiography - SonoVue	0	218	0.00228 [0.00014, 0.03540]
Soman 2000 - rest echocardiography - SonoVue	0	13	0.03571 [0.00220, 0.38387]
Wei 2012 - rest echocardiography - Definity	0	32	0.01515 [0.00094, 0.20079]
Dolan 2009 - stress echocardiography - Definity	0	5947	0.00008 [0.00001, 0.00134]
Dolan 2009 - stress echocardiography - Optison	0	12802	0.00004 [0.00000, 0.00062]
Furtado 2021 - stress echocardiography - SonoVue	0	1062	0.00047 [0.00003, 0.00747]
Wever-Pinzon 2012 - stress echocardiography - Definity	0	1513	0.00033 [0.00002, 0.00526]
Timperley 2005 - stress echocardiography - SonoVue	0	299	0.00167 [0.00010, 0.02606]
Timperley 2005 - stress echocardiography - Optison	0	120	0.00413 [0.00026, 0.06255]
Abdelmoneim 2009 - other - Optison or Definity	0	10792	0.00005 [0.00000, 0.00074]
Anantharam 2009 - - Luminity	0	461	0.00108 [0.00007, 0.01705]
Anantharam 2009 - - SonoVue or Luminity	0	689	0.00072 [0.00005, 0.01147]
Argulian 2020 - - Optison	0	33	0.01471 [0.00091, 0.19588]
Random-Effects Model: $\tau^2 = 6.64$; $I^2 = 86.9\%$			0.00002 [0.00000, 0.00020]

Test for overall effect: Z = -8.78 (<0.001)

Proportion (logit back-transformed)

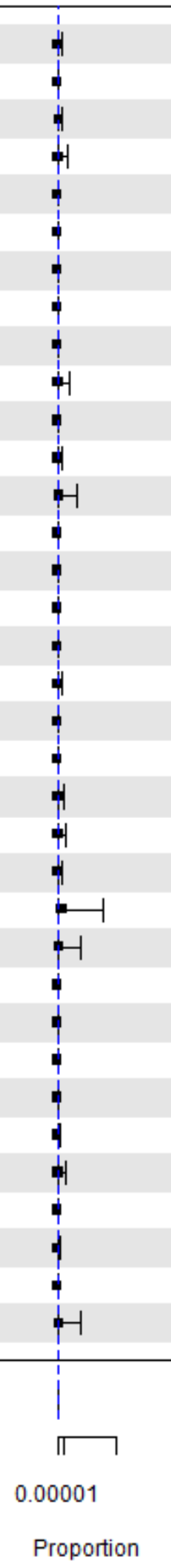

Figure S9 - Forest Plot Severe Adverse Events Non Cardiologic – Adults

Study	N events	Total patients	Proportion [95% CI]
shang 2023 - abdominal ceus - sonovue	17	463434	0.00004 [0.00002, 0.00006]
li 2023 - abdominal ceus - sonovue	7	49100	0.00014 [0.00007, 0.00030]
li 2024 - abdominal ceus - sonovue	5	83778	0.00006 [0.00002, 0.00014]
hu 2019 - abdominal ceus - sonovue	3	34478	0.00009 [0.00003, 0.00027]
piscaglia 2006 - abdominal ceus - sonovue	2	23188	0.00009 [0.00002, 0.00034]
kaps 2001 - - sonovue	0	40	0.01220 [0.00076, 0.16720]
morel 2000 - - sonovue	0	12	0.03846 [0.00236, 0.40319]
kaps 1999 - - sonovue	0	12	0.03846 [0.00236, 0.40319]
chou 2019 - abdominal ceus - sonazoid	0	54	0.00909 [0.00057, 0.12933]
lv 2021 - abdominal ceus - sonazoid	0	218	0.00228 [0.00014, 0.03540]
lv 2021 - abdominal ceus - sonovue	0	206	0.00242 [0.00015, 0.03739]
madjar 2000 - breast ceus - sonovue	0	40	0.01220 [0.00076, 0.16720]
miyamoto 2014 - breast ceus - sonazoid	0	117	0.00424 [0.00026, 0.06405]
fischer 2020 - - sonovue	0	764	0.00065 [0.00004, 0.01036]
wu 2023 - - definity.1	0	55	0.00893 [0.00056, 0.12728]
wu 2023 - - definity.2	0	43	0.01136 [0.00071, 0.15733]
nguyen 2025 - - sonovue	0	15	0.03125 [0.00193, 0.35028]
bogdahn 1999 - transcranial doppler - sonovue	0	10	0.04545 [0.00278, 0.44831]
baracchini 2017 - - sonovue	0	92	0.00538 [0.00034, 0.08009]
bokor 2001 - - sonovue.1	0	44	0.01111 [0.00069, 0.15430]
bokor 2001 - - sonovue.2	0	12	0.03846 [0.00236, 0.40319]
			0.00006 [0.00004, 0.00011]

Proportion (logit back-transformed)

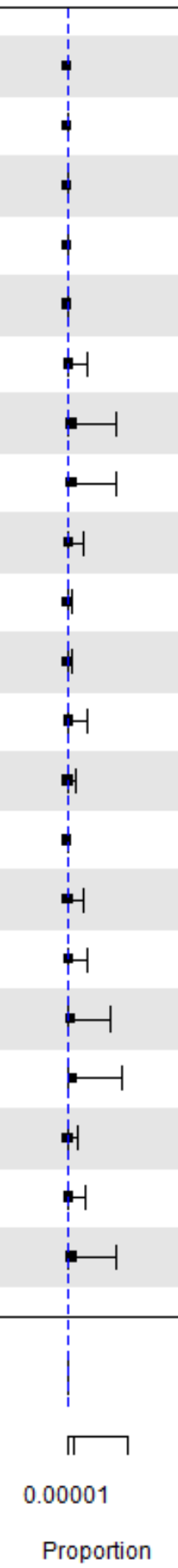

Random-Effects Model: $\tau^2 = 0.15$; $I^2 = 19.6\%$

Test for overall effect: Z = -35 (<0.001)

Figure S10 - Forest Plot Severe Adverse Events Non Cardiologic – Children

Study	N events	Total patients	Proportion [95% CI]	Proportion (logit back-transformed)
dietrich 2021 - abdominal ceus - sonovue	2	1676	0.00119 [0.00030, 0.00476]	
piskunovicz 2015 - abdominal ceus - sonovue	1	137	0.00730 [0.00103, 0.04995]	
coleman 2014 - abdominal ceus - optison	0	32	0.01515 [0.00094, 0.20079]	
mao 2019 - abdominal ceus - sonovue	0	312	0.00160 [0.00010, 0.02500]	
seelbach 2022 - - sonovue	0	30	0.01613 [0.00100, 0.21141]	
stenzel 2013 - abdominal ceus - sonovue	0	37	0.01316 [0.00082, 0.17839]	
torres 2017 - abdominal ceus - sonovue	0	173	0.00287 [0.00018, 0.04421]	
knieling 2016 - abdominal ceus - sonovue	0	40	0.01220 [0.00076, 0.16720]	
ntoulia 2024 - - optison	0	12	0.03846 [0.00236, 0.40319]	
ntoulia 2024 - - sonovue	0	4	0.10000 [0.00595, 0.67360]	
riggs 2024 - - sonovue	0	38	0.01282 [0.00080, 0.17450]	
			0.00120 [0.00039, 0.00373]	

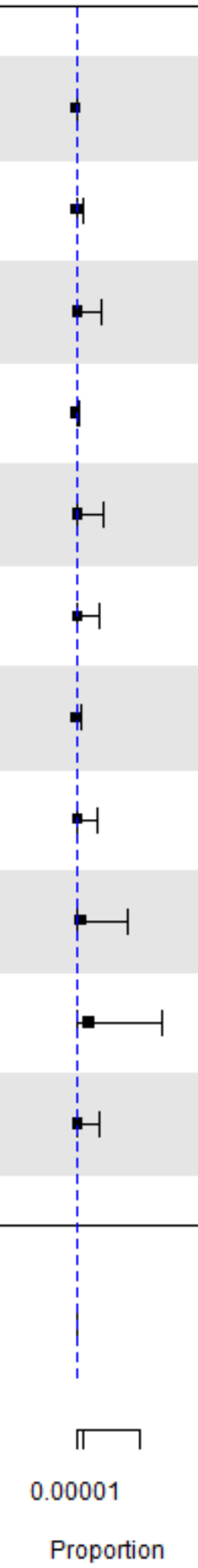

ts Model: $\tau^2 = 0$; $I^2 = 0\%$

ffect: Z = -11.63 (<0.001)

Figure S11 - Forest Plot Non Severe Adverse Events Non Cardiologic – Adults

Study	N events	Total patients	Proportion [95% CI]
shang 2023 - abdominal ceus - sonovue	136	463434	0.00029 [0.00025, 0.00035]
hu 2019 - abdominal ceus - sonovue	36	34478	0.00104 [0.00075, 0.00145]
li 2023 - abdominal ceus - sonovue	36	49100	0.00073 [0.00053, 0.00102]
piscaglia 2006 - abdominal ceus - sonovue	22	23188	0.00095 [0.00062, 0.00144]
li 2024 - abdominal ceus - sonovue	15	83778	0.00018 [0.00011, 0.00030]
kaps 1999 - - sonovue	8	12	0.66667 [0.37588, 0.86914]
baracchini 2017 - - sonovue	6	92	0.06522 [0.02959, 0.13764]
fischer 2020 - - sonovue	3	764	0.00393 [0.00127, 0.01210]
madjar 2000 - breast ceus - sonovue	2	40	0.05000 [0.01254, 0.17909]
bokor 2001 - - sonovue.1	2	44	0.04545 [0.01140, 0.16438]
bokor 2001 - - sonovue.2	2	12	0.16667 [0.04198, 0.47720]
kaps 2001 - - sonovue	0	40	0.01220 [0.00076, 0.16720]
morel 2000 - - sonovue	0	12	0.03846 [0.00236, 0.40319]
chou 2019 - abdominal ceus - sonazoid	0	54	0.00909 [0.00057, 0.12933]
lv 2021 - abdominal ceus - sonazoid	0	218	0.00228 [0.00014, 0.03540]
lv 2021 - abdominal ceus - sonovue	0	206	0.00242 [0.00015, 0.03739]
miyamoto 2014 - breast ceus - sonazoid	0	117	0.00424 [0.00026, 0.06405]
wu 2023 - - definity.1	0	55	0.00893 [0.00056, 0.12728]
wu 2023 - - definity.2	0	43	0.01136 [0.00071, 0.15733]
nguyen 2025 - - sonovue	0	15	0.03125 [0.00193, 0.35028]
bogdahn 1999 - transcranial doppler - sonovue	0	10	0.04545 [0.00278, 0.44831]
			0.00217 [0.00048, 0.00971]

Random-Effects Model: $\tau^2 = 7.58$; $I^2 = 98.6\%$

Test for overall effect: Z = -7.99 (<0.001)

Proportion (logit back-transformed)

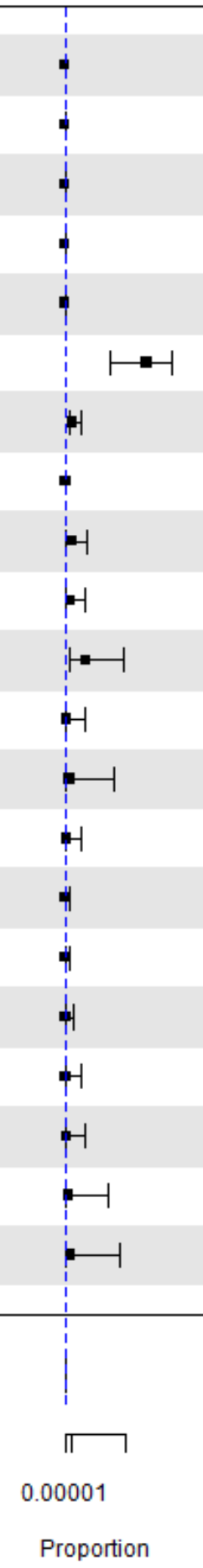

Figure S12 - Forest Plot Non Severe Adverse Events Non Cardiologic – Children

Study	N events	Total patients	Proportion [95% CI]
dietrich 2021 - abdominal ceus - sonovue	13	1676	0.00776 [0.00451, 0.01331]
mao 2019 - abdominal ceus - sonovue	6	312	0.01923 [0.00866, 0.04213]
coleman 2014 - abdominal ceus - optison	3	32	0.09375 [0.03055, 0.25350]
knieling 2016 - abdominal ceus - sonovue	2	40	0.05000 [0.01254, 0.17909]
seelbach 2022 - - sonovue	1	30	0.03333 [0.00468, 0.20200]
stenzel 2013 - abdominal ceus - sonovue	1	37	0.02703 [0.00379, 0.16847]
torres 2017 - abdominal ceus - sonovue	0	173	0.00287 [0.00018, 0.04421]
piskunovicz 2015 - abdominal ceus - sonovue	0	137	0.00362 [0.00023, 0.05520]
ntoulia 2024 - - optison	0	12	0.03846 [0.00236, 0.40319]
ntoulia 2024 - - sonovue	0	4	0.10000 [0.00595, 0.67360]
riggs 2024 - - sonovue	0	38	0.01282 [0.00080, 0.17450]
			0.01229 [0.00512, 0.02921]

Proportion (logit back-transformed)

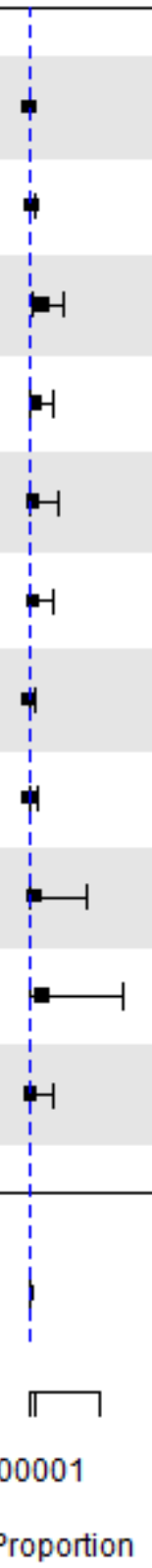

Random-Effects Model: $\tau^2 = 0.9$; $I^2 = 64.3\%$

Test for overall effect: Z = -9.74 (<0.001)

Figure S13 - Forest Plot Delayed Adverse Events Cardiologic – Adults

Study	N events	Total patients	Proportion [95% CI]
kitzman 2000 - - definity	2	169	0.011834320 [0.002961703, 0.046059438]
furtado 2021 - stress echocardiography - sonovue	1	1062	0.000941620 [0.000132625, 0.006652554]
geleijnse 2009 - - sonovue	0	274	0.001818182 [0.000113632, 0.028366563]
fang 2004 - - sonovue	0	90	0.005494505 [0.000342824, 0.081732051]
herzog 2008 - stress echocardiography - optison	0	3051	0.000163827 [0.000010246, 0.002613362]
herzog 2008 - - definity	0	6959	0.000071839 [0.000004493, 0.001147407]
kumar 2019 - stress echocardiography - definity	0	3306	0.000151194 [0.000009456, 0.002412261]
kumar 2019 - stress echocardiography - sonovue	0	2137	0.000233863 [0.000014626, 0.003727038]
platts 2013 - stress echocardiography - definity	0	5576	0.000089654 [0.000005608, 0.001431597]
suma 2016 - stress echocardiography - definity	0	179	0.002777778 [0.000173530, 0.042792515]
suma 2016 - stress echocardiography - optison	0	40	0.012195122 [0.000758572, 0.167202611]
wei 2008 - stress echocardiography - optison	0	12219	0.000040917 [0.000002559, 0.000653789]
wei 2014 - stress echocardiography - optison	0	1039	0.000480769 [0.000030065, 0.007636364]
weiss 2012 - stress echocardiography - definity	0	1053	0.000474383 [0.000029666, 0.007535582]
cohen 1998 - rest echocardiography - optison	0	198	0.002512563 [0.000156980, 0.038841880]
dolan 2009 - rest echocardiography - optison or definity	0	23659	0.000021133 [0.000001322, 0.000337762]
kusnetzky 2008 - rest echocardiography - definity	0	6196	0.000080684 [0.000005047, 0.001288525]
nanda 2002 - rest echocardiography - sonovue	0	138	0.003597122 [0.000224632, 0.054825658]
nucifora 2008 - rest echocardiography - definity	0	115	0.004310345 [0.000269084, 0.065093796]
senior 2000 - rest echocardiography - sonovue	0	218	0.002283105 [0.000142659, 0.035401562]
soman 2000 - rest echocardiography - sonovue	0	13	0.035714286 [0.002196902, 0.383867140]
wei 2012 - rest echocardiography - definity	0	32	0.015151515 [0.000941179, 0.200793972]
dolan 2009 - stress echocardiography - definity	0	5947	0.000084062 [0.000005258, 0.001342405]
dolan 2009 - stress echocardiography - optison	0	12802	0.000039053 [0.000002443, 0.000624034]
wever-pinzon 2012 - stress echocardiography - definity	0	1513	0.000330251 [0.000020654, 0.005256279]
timperley 2005 - stress echocardiography - sonovue	0	299	0.001666667 [0.000104170, 0.026055100]
timperley 2005 - stress echocardiography - optison	0	120	0.004132231 [0.000257985, 0.062547220]
abdelmoneim 2009 - other - optison or definity	0	10792	0.000046326 [0.000002898, 0.000740176]
anantharam 2009 - - definity	0	461	0.001082251 [0.000067661, 0.017051468]
anantharam 2009 - - sonovue or definity	0	689	0.000724638 [0.000045311, 0.011472071]
argulian 2020 - - optison	0	33	0.014705882 [0.000913687, 0.195875248]
			0.000000003 [0.000000000, 0.319120985]

Random-Effects Model: $\tau^2 = 59.67$; $I^2 = 97.1\%$

Test for overall effect: Z = -2.04 (0.041)

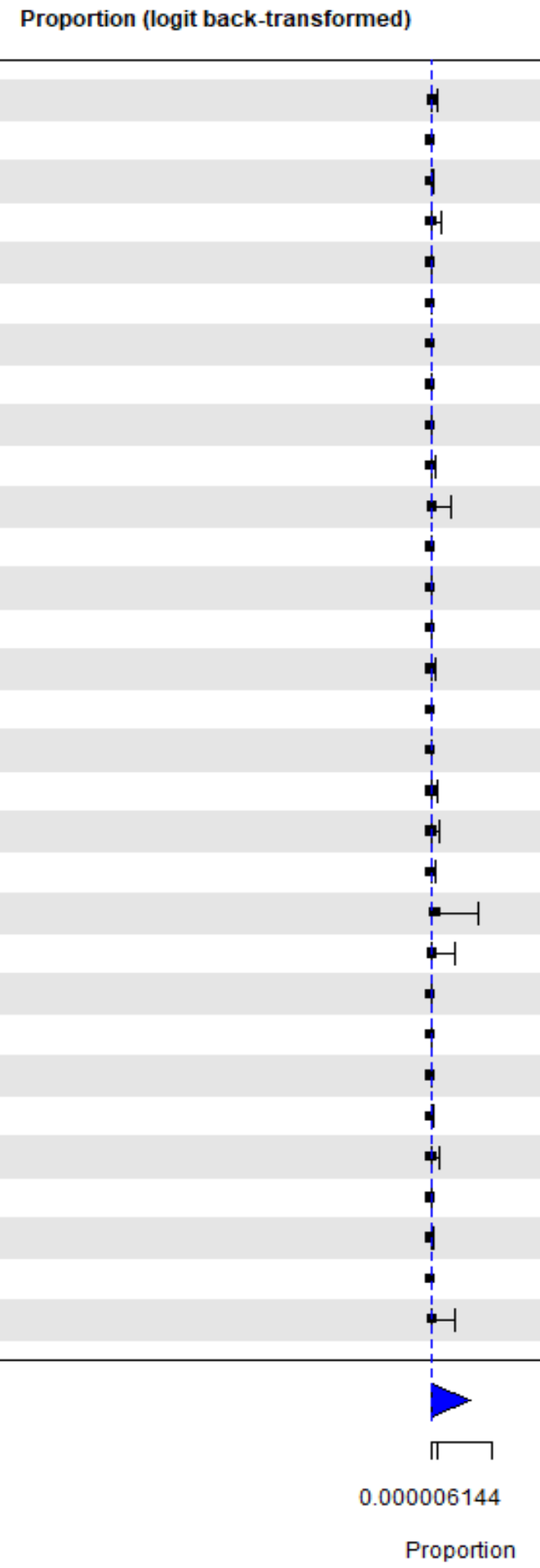

Figure S14 - Forest Plot Delayed Adverse Events Non Cardiologic – Adults

Study	N events	Total patients	Proportion [95% CI]
piscaglia 2006 - abdominal ceus - sonovue	5	23188	0.0002156 [0.0000898, 0.0005179]
miyamoto 2014 - breast ceus - sonazoid	4	117	0.0341880 [0.0128908, 0.0875505]
hu 2019 - abdominal ceus - sonovue	1	34478	0.0000290 [0.0000041, 0.0002059]
kaps 2001 - - sonovue	0	40	0.0121951 [0.0007586, 0.1672026]
morel 2000 - - sonovue	0	12	0.0384615 [0.0023627, 0.4031937]
kaps 1999 - - sonovue	0	12	0.0384615 [0.0023627, 0.4031937]
chou 2019 - abdominal ceus - sonazoid	0	54	0.0090909 [0.0005663, 0.1293346]
lv 2021 - abdominal ceus - sonazoid	0	218	0.0022831 [0.0001427, 0.0354016]
lv 2021 - abdominal ceus - sonovue	0	206	0.0024155 [0.0001509, 0.0373885]
madjar 2000 - breast ceus - sonovue	0	40	0.0121951 [0.0007586, 0.1672026]
shang 2023 - abdominal ceus - sonovue	0	463434	0.0000011 [0.0000001, 0.0000172]
li 2023 - abdominal ceus - sonovue	0	49100	0.0000102 [0.0000006, 0.0001628]
li 2024 - abdominal ceus - sonovue	0	83778	0.0000060 [0.0000004, 0.0000954]
fischer 2020 - - sonovue	0	764	0.0006536 [0.0000409, 0.0103573]
wu 2023 - - definity.1	0	55	0.0089286 [0.0005562, 0.1272756]
wu 2023 - - definity.2	0	43	0.0113636 [0.0007071, 0.1573318]
nguyen 2025 - - sonovue	0	15	0.0312500 [0.0019265, 0.3502756]
bogdahn 1999 - transcranial doppler - sonovue	0	10	0.0454545 [0.0027827, 0.4483136]
baracchini 2017 - - sonovue	0	92	0.0053763 [0.0003355, 0.0800943]
bokor 2001 - - sonovue.1	0	44	0.0111111 [0.0006915, 0.1542955]
bokor 2001 - - sonovue.2	0	12	0.0384615 [0.0023627, 0.4031937]
			0.0000031 [0.0000000, 0.0003209]

Random-Effects Model: $\tau^2 = 21.46$; $I^2 = 94.6\%$

Test for overall effect: Z = -5.35 (<0.001)

Proportion (logit back-transformed)

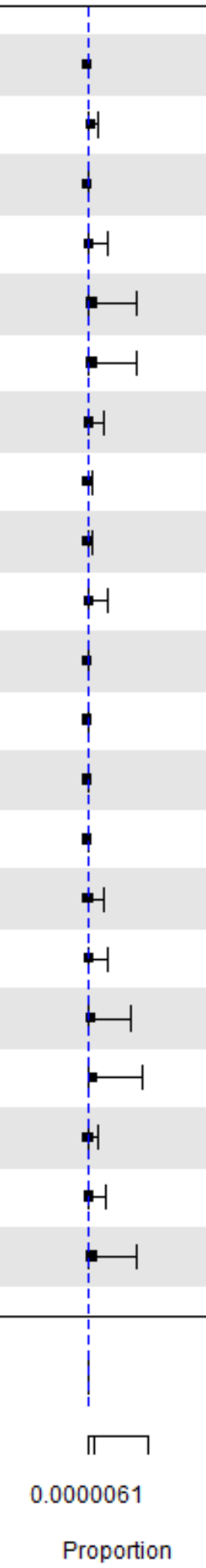

References

PICO 3

1. Dietrich CF, Nolsøe CP, Barr RG, Berzigotti A, Burns PN, Cantisani V, et al. Guidelines and Good Clinical Practice Recommendations for Contrast-Enhanced Ultrasound (CEUS) in the Liver–Update 2020 WFUMB in Cooperation with EFSUMB, AFSUMB, AIUM, and FLAUS. Ultrasound in Medicine & Biology. 2020 Oct;46(10):2579–604.
2. Amir-Kabirian B., Radow M. A Luminating Reaction: Anaphylactic Shock Following Lumason®® Administration. American Journal of Respiratory and Critical Care Medicine [Internet]. 2024;209((Amir-Kabirian) Internal Medicine, Charleston Area Medical Centre, West Virginia University Charleston, Charleston, WV, United States(Radow) Pulmonology/Critical Care, Charleston Area Medical Centre, Charleston, WV, United States). Available from: https://www.atsjournals.org/doi/pdf/10.1164/ajrccm-conference.2024.209.1_MeetingAbstracts.A1354https://ovidsp.ovid.com/ovidweb.cgi?T=JS&CSC=Y&NEWS=N&PAGE=fulltext&D=emed27&DO=10.1164%2fajrccm-conference.2024.209.1_meetingabstracts.a1359https://unipd.primo
3. Arnouk S, Huynh Q, Saric M, Papadopoulos J. A Case Report of Cardiac Arrest After Intravenous Administration of Sulfur Hexafluoride (Lumason®®(®))
Ultrasound Enhancing Agent. J Pharm Pract. 2023;8971900231158935.
4. Blokh I, Ganem A, Bonaros EP, Stephen BD, Steinberg B, Rosen SE. Hemodynamic instability after receiving intravenous perflutren for contrast echocardiography in an elderly female. Echocardiography. 2004;21(7):613–5.
5. Calvo D, de la Hera J, Lee DH. Contrast echocardiography and clinical safety [3]. Revista Espanola de Cardiologia. 2006;59(4):399–400.
6. Coudray S, Fabre C, Aichoun I, Perez-Martin A. Anaphylactic shock with an ultrasound contrast agent. J Med Vasc. 2017;42(6):384–7.
7. Crucinio N, Nacchiero MC, Panella C, Ierardi E. A case of adverse reaction potentially related to a microbubble contrast agent for ultrasonography. European Journal of Radiology Extra. 2004;52(1):25–6.
8. D'Amico A., Mir N., Van Wagoner N., Wilkerson H., Kanter J. Acute Vaso-Occlusive crisis with splenic sequestration in a person with sickle cell disease following perflutren lipid microsphere administration for cardiovascular ultrasound enhancement. Journal of General Internal Medicine. 2020;35(SUPPL 1):S361.
9. Geleijnse ML, Nemes A, Vletter WB, Michels M, Soliman OI, Caliskan K, et al. Adverse reactions after the use of sulphur hexafluoride (SonoVue®) echo contrast agent. J Cardiovasc Med (Hagerstown). 2009;10(1):75–7.
10. Gupta A., Harris S., Naina H. Perflutren-Induced Angioedema. Echocardiography. 2015;32(8):1329.

11. Hadid H. Lumason®® Administration Leading to Anaphylactic Shock.American Journal of Respiratory and Critical Care Medicine [Internet]. 2024;209((Hadid) Pulmonary and Critical Care Medicine, John Dingell Detroit VA medical center, Detroit, MI, United States). Available from: https://www.atsjournals.org/doi/pdf/10.1164/ajrccm-conference.2024.209.1_MeetingAbstracts.A1628https://ovidsp.ovid.com/ovidweb.cgi?T=JS&CSC=Y&NEWS=N&PAGE=fulltext&D=emed27&DO=10.1164%2fajrccm-conference.2024.209.1_meetingabstracts.a1628https://unipd.primo
12. Herzog CA. Incidence of adverse events associated with use of perflutren contrast agents for echocardiography. JAMA. 2008;299(17):2023–5.
13. Hu C, Feng Y, Huang P, Jin J. Adverse reactions after the use of SonoVue® contrast agent: Characteristics and nursing care experience. Medicine (Baltimore). 2019;98(44):e17745.
14. Ionescu A. Bubble trouble: anaphylactic shock, threatened myocardial infarction, and transient renal failure after intravenous echo contrast for left ventricular cavity opacification preceding dobutamine stress echo. Eur J Echocardiogr. 2009;10(5):707–10.
15. Krantz M.S., Liu Y., Phillips E.J., Stone C.A. Anaphylaxis to PEGylated liposomal echocardiogram contrast in a patient with IgE-mediated macrogol allergy. Journal of Allergy and Clinical Immunology: In Practice. 2020;8(4):1416-1419.e3.
16. Levano JA, Jimenez MA, Laiseca A, Vives R. Anaphylactic shock due to SonoVue®. Ann Allergy Asthma Immunol. 2012;108(3):208–9.
17. Longhino D, Buonomo A, Zocco MA, Ainora ME, Esposto G, Mignini I, et al. Anaphylaxis after SonoVue®: A Case Report and a Literature Review. J Clin Med. 2024;13(17).
18. Mahjoub H, Roméo P, Leung TK, Burelle D, Cartier R, Basmadjian AJ. Sudden death after intravenous administration of a perflutren contrast agent: a case of pseudocomplication? J Am Soc Echocardiogr. 2009 June;22(6):753.e5-8.
19. Mansour A., Shinn A., Bataineh M.A., Aggarwal K.B. RARE ANAPHYLACTIC REACTION TO AN ECHOCARDIOGRAPHY CONTRAST AGENT. Journal of the American College of Cardiology. 2022;79(9 Supplement):3116.
20. Marchi E, Sciara P, Casini M, Migliorini A, Valenti R, Muraca I. Subacute Stent Thrombosis Related to Kounis Syndrome During Anaphylaxis Induced by Ultrasound Contrast Agent Infusion. JACC Case Rep. 2024;29(21):102612.
21. Mikhail ES, Ghaly JB, Malik B, Ghatol AG. Anaphylaxis to Lumason®® Contrast Agent in Echocardiography after Immune Sensitization: A Case Report and Literature Review. J Community Hosp Intern Med Perspect. 2023;13(4):74–8.

22. Minc A., Baldwin J., Kolias T., LaBounty T. A CASE OF ANAPHYLAXIS TO CARDIAC ULTRASOUND ENHANCING AGENT. Annals of Allergy, Asthma and Immunology. 2024;133(6 Supplement):S112.
23. Mir T, Uddin MM, Watson K, Meir EB, Abdo A. Definity®, echo contrast, induced cardiac arrest: brief review of the literature. BMJ Case Rep. 2021;14(4).
24. Nigolian A, Nigolian H, Harr T, Cook S. Anaphylactic shock and cardiac arrest after intravenous injection of sulphur hexafluoride SonoVue®. BMJ Case Rep. 2024;17(11).
25. Nomigolzar S., Khederzadeh S., Baggett A., Vallabhaneni M., Younes A. Type I Kounis Syndrome Induced by Echocardiography Contrast Agent Sulfur Hexafluoride. Circulation. 2022;146(Supplement 1).
26. Olson MC, Atwell TD, Knudsen JM. Anaphylactic reaction to an ultrasound contrast agent (Lumason®®) in a patient with systemic mastocytosis. J Clin Ultrasound. 2018;46(8):533–5.
27. Piscaglia F, Bolondi L, Aiani L, Luigi Angeli M, Arienti V, Barozzi L, et al. The safety of SonoVue®® in abdominal applications: Retrospective analysis of 23188 investigations. Ultrasound in Medicine and Biology. 2006;32(9):1369–75.
28. Piskunowicz M, Kosiak W, Batko T, Piankowski A, Połczyńska K, Adamkiewicz-Drożyńska E. Safety of Intravenous Application of Second-Generation Ultrasound Contrast Agent in Children: Prospective Analysis. Ultrasound in Medicine & Biology. 2015 Apr 1;41(4):1095–9.
29. Sagalov A., Eggert A., Rimawi A., Hegde S. A Rare Presentation of Kounis Syndrome Induced by an Echocardiography Contrast. CJC Open. 2023;5(10):757EP – 759.
30. Samannan R., Arif M.A., Bhatti U., Munzinger E., Mathew S. Echocontrast induced angioedema. Cardiology (Switzerland). 2018;140(Supplement 1):221.
31. Shang Y, Xie X, Luo Y, Nie F, Jing X, Liao J, et al. Safety findings after intravenous administration of sulfur hexafluoride microbubbles to 463,434 examinations at 24 centers. Eur Radiol. 2023;33(2):988–95.
32. Solivetti FM, Elia F, Musicco F, Bonagura AC, Di Leo N, Iera J, et al. Anaphylactic shock induced by sulphur hexafluoride in an individual with no history of heart disease: case report and literature review. Ultraschall Med. 2012;33(6):597–8.
33. Steinhauer R., Miyashita S., Maslon L., Chweich H., Downey B. A Rare Case of Echo Contrast Anaphylaxis Complicated by Acute Coronary Syndrome. Circulation. 2022;146(Supplement 1).
34. Tang C, Fang K, Guo Y, Li R, Fan X, Chen P, et al. Safety of Sulfur Hexafluoride Microbubbles in Sonography of Abdominal and Superficial Organs: Retrospective Analysis of 30,222 Cases. J Ultrasound Med. 2017;36(3):531–8.

35. van Ginkel AG, Sorgdrager BJ, de Graaf MA, Karalis I, Ajmone Marsan N. ST-segment elevation associated with allergic reaction to echocardiographic contrast agent administration. Netherlands Heart Journal. 2014;22(2):77–9.
36. Wei K, Mulvagh SL, Carson L, Davidoff R, Gabriel R, Grimm RA, et al. The safety of Definity® and Optison® for ultrasound image enhancement: a retrospective analysis of 78,383 administered contrast doses. J Am Soc Echocardiogr. 2008;21(11):1202–6.

PICO 1, 2, 4

1. Abo-Salem E., Lopez-Candales A. Diagnosis of asymptomatic aortic dissection during pregnancy using contrast echocardiography. Journal of Cardiology Cases. 2014;9(5):200–2.
2. Dietrich CF, Nolsøe CP, Barr RG, Berzigotti A, Burns PN, Cantisani V, et al. Guidelines and Good Clinical Practice Recommendations for Contrast-Enhanced Ultrasound (CEUS) in the Liver–Update 2020 WFUMB in Cooperation with EFSUMB, AFSUMB, AIUM, and FLAUS. Ultrasound in Medicine & Biology. 2020 Oct;46(10):2579–604.
3. Geyer T, Rübenthaler J, Froelich MF, Sabel L, Marschner C, Schwarze V, et al. Contrast-Enhanced Ultrasound for Assessing Abdominal Conditions in Pregnancy. Medicina (Kaunas). 2020 Dec 8;56(12):675.
4. Abdelmoneim SS, Bernier M, Scott CG, Dhoble A, Ness SA, Hagen ME, et al. Safety of contrast agent use during stress echocardiography: a 4-year experience from a single-center cohort study of 26,774 patients. JACC Cardiovasc Imaging. 2009;2(9):1048–56.
5. Anantharam B, Chahal N, Chelliah R, Ramzy I, Gani F, Senior R. Safety of contrast in stress echocardiography in stable patients and in patients with suspected acute coronary syndrome but negative 12-hour troponin. Am J Cardiol. 2009;104(1):14–8.
6. Argulian E., Sud K., Bohra C., Vogel B., Garg V., Talebi S., et al. Safety of Ultrasonic Enhancing Agents in Patients with COVID-19. Journal of the American Society of Echocardiography. 2020;33(7):906–8.
7. Baracchini C, Viaro F, Favaretto S, Palmieri A, Kulyk C, Causin F, et al. Safety and Tolerability of SonoVue®® in Patients with Large Artery Anterior Circulation Acute Stroke. J Neuroimaging. 2017;27(4):409–13.
8. Bogdahn U, Hölscher T, Rosin L, Götz B, Schlachetzki F. Contrast-Enhanced Transcranial and Extracranial Duplex Sonography: Preliminary Results of a Multicenter Phase II/III Study with SonoVue®trade mark. Echocardiography. 1999;16(7, Pt 2):761–6.
9. Bokor D, Chambers JB, Rees PJ, Mant TG, Luzzani F, Spinazzi A. Clinical safety of SonoVue®, a new contrast agent for ultrasound imaging, in healthy volunteers and in patients with chronic obstructive pulmonary disease. Invest Radiol. 2001;36(2):104–9.
10. Chou YH, Liang JD, Wang SY, Hsu SJ, Hu JT, Yang SS, et al. Safety of Perfluorobutane (Sonazoid®) in Characterizing Focal Liver Lesions. J Med Ultrasound. 2019;27(2):81–5.
11. Cohen JL, Cheirif J, Segar DS, Gillam LD, Gottdiener JS, Hausnerova E, et al. Improved left ventricular endocardial border delineation and opacification with OPTISON® (FS069), a new echocardiographic contrast agent. Results of a phase III Multicenter Trial. J Am Coll Cardiol. 1998;32(3):746–52.

12. Coleman JL, Navid F, Furman WL, McCarville MB. Safety of ultrasound contrast agents in the pediatric oncologic population: A single-institution experience. American Journal of Roentgenology. 2014;202(5):966–70.
13. Cvitkovic-Roic A, Turudic D, Milosevic D, Palcic I, Roic G. Contrast-enhancedvoiding urosonography in the diagnosis of intrarenal reflux. J Ultrasound. 2022 Mar 1;25(1):89–95.
14. Dolan MS, Gala SS, Dodla S, Abdelmoneim SS, Xie F, Cloutier D, et al. Safety and efficacy of commercially available ultrasound contrast agents for rest andstress echocardiography a multicenter experience. J Am Coll Cardiol. 2009;53(1):32–8.
15. Duran C, Del Riego J, Riera L, Martin C, Serrano C, Palaña P. Voiding urosonography including urethrosonography: High-quality examinations with an optimised procedure using a second-generation US contrast agent. Pediatric Radiology. 2012;42(6):660–7.
16. Faizah MZ, Hamzaini AH, Kanaheswari Y, Dayang A AA, Zulfiqar MA. Contrast enhanced Voiding Urosonography (ce-VUS) as a radiation-free technique in the diagnosis of vesicoureteric reflux: Our early experience. Med J Malaysia. 2015 Oct 1;70(5):269–72.
17. Fang LG. [The effect of SonoVue® in endocardial border delineation and myocardial perfusion]. Zhonghua Nei Ke Za Zhi. 2004;43(11):824–7.
18. Fischer C, Kunz P, Strauch M, Weber MA, Doll J. Safety Profile of Musculoskeletal Contrast-Enhanced Ultrasound with Sulfur Hexafluoride Contrast Agent. Ther Clin Risk Manag. 2020;16:269–80.
19. Furtado RG, Rassi DDC, Melato LH, Oliveira ACR, Nunes PM, Baccelli PE, et al. Safety of SF6(SonoVue®®) Contrast Agent on Pharmacological Stress Echocardiogram. Arq Bras Cardiol. 2021;117(6):1170–8.
20. Geleijnse ML, Nemes A, Vletter WB, Michels M, Soliman OI, Caliskan K, et al. Adverse reactions after the use of sulphur hexafluoride (SonoVue®) echo contrast agent. J Cardiovasc Med (Hagerstown). 2009;10(1):75–7.
21. Herzog CA. Incidence of adverse events associated with use of perflutren contrast agents for echocardiography. JAMA. 2008;299(17):2023–5.
22. Hu C, Feng Y, Huang P, Jin J. Adverse reactions after the use of SonoVue® contrast agent: Characteristics and nursing care experience. Medicine (Baltimore). 2019;98(44):e17745.
23. Kaps M, Legemate DA, Ries F, Ackerstaff RG, Markus H, Pezzoll C, et al. SonoVue® in transcranial Doppler investigations of the cerebral arteries. J Neuroimaging. 2001;11(3):261–7.
24. Kaps M, Seidel G, Bokor D, Modrau B, Algermissen C. Safety and ultrasound-enhancing potentials of a new sulfur hexafluoride-containing agent in the cerebral circulation. J Neuroimaging. 1999;9(3):150–4.
25. Kim T, Jang H, Ison SRW. Microbubble Contrast Agents for Ultrasound Imaging — Safety and Efficacy in Abdominal and Vascular Imaging. In 2008 [cited 2025 Aug 25]. Available from: https://www.semanticscholar.org/paper/Microbubble-Contrast-Agents-

for-Ultrasound-Imaging-Kim-Jang/c68ada1f04b194cc56b553d3e8867eccb188811d
26. Kis É, Nyitrai A, Várkonyi I, Máttyus I, Cseprekál O, Reusz G, et al. Voiding urosonography with second-generation contrast agent versus voiding cystourethrography. Pediatr Nephrol. 2010 Nov 1;25(11):2289–93.
27. Kitzman DW, Goldman ME, Gillam LD, Cohen JL, Aurigemma GP, Gottdiener JS. Efficacy and safety of the novel ultrasound contrast agent perflutren (Definity®) in patients with suboptimal baseline left ventricular echocardiographic images. Am J Cardiol. 2000;86(6):669–74.
28. Ključevšek D, Battelino N, Tomažič M, Kersnik Levart T. A comparison of echo-enhanced voiding urosonography with X-ray voiding cystourethrography in the first year of life. Acta Paediatrica. 2012;101(5):e235–9.
29. Knieling F, Strobel D, Rompel O, Zapke M, Menendez-Castro C, Wölfel M, et al. Spectrum, Applicability and Diagnostic Capacity of Contrast-Enhanced Ultrasound in Pediatric Patients and Young Adults after Intravenous Application – A Retrospective Trial. Ultraschall Med. 2016 Dec;37(6):619–26.
30. Kumar S, Purtell C, Peterson A, Gibbons P, Khan AM, Heitner SB. Safety profile of ultrasound enhancing agents in echocardiography. Echocardiography. 2019;36(6):1041–4.
31. Kusnetzky LL, Khalid A, Khumri TM, Moe TG, Jones PG, Main ML. Acute mortality in hospitalized patients undergoing echocardiography with and without an ultrasound contrast agent: results in 18,671 consecutive studies. J Am Coll Cardiol. 2008;51(17):1704–6.
32. Kutty S, Xiao Y, Olson J, Xie F, Danford DA, Erickson CC, et al. Safety and Efficacy of Cardiac Ultrasound Contrast in Children and Adolescents for Resting and Stress Echocardiography. J Am Soc Echocardiogr. 2016;29(7):655–62.
33. Kuzmanovska D, Risteski A, Kambovska M, Trpcevski T, Sahpazova E, Petrovski M. Voiding Urosonography with Second-Generation Ultrasound Contrast Agent for Diagnosis of Vesicoureteric Reflux: First Local Pilot Study. Open Access Maced J Med Sci. 2017;5(2):215–21.
34. Li D, Zhang R, Lan H, Chen M, Huang Z, Zhao H, et al. A retrospective study on adverse events of intravenous administration of sulfur hexafluoride microbubbles in abdominal and superficial applications in 83,778 patients. Insights Imaging. 2024;15(1):65.
35. Li Q., Yang K., Ji Y., Liu H., Fei X., Zhang Y., et al. Safety Analysis of Adverse Events of Ultrasound Contrast Agent Lumason®®/SonoVue® in 49,100 Patients. Ultrasound in medicine & biology. 2022;((Li, Yang, Ji, Liu, Fei, Zhang) Department of Ultrasound, First Medical Center, Chinese PLA General Hospital, Beijing, China(Li) Department of Ultrasound, First Medical Center, Chinese PLA General Hospital, Beijing, China; Medical School of Chinese PLA, B).

36. Lv K, Zhai H, Jiang Y, Liang P, Xu HX, Du L, et al. Prospective assessment of diagnostic efficacy and safety of Sonazoid®(TM) and SonoVue®(®) ultrasound contrast agents in patients with focal liver lesions. Abdom Radiol (NY). 2021;46(10):4647–59.
37. Madjar H, Prömpeler HJ, Del Favero C, Hackelöer BJ, Llull JB. A new Doppler signal enhancing agent for flow assessment in breast lesions. Eur J Ultrasound. 2000;12(2):123–30.
38. Mao M, Xia B, Chen W, Gao X, Yang J, Li S, et al. The Safety and Effectiveness of Intravenous Contrast-Enhanced Sonography in Chinese Children-A Single Center and Prospective Study in China. Front Pharmacol. 2019;10:1447.
39. Marschner CA, Schwarze V, Stredele R, Froelich MF, Rübenthaler J, Geyer T, et al. Safety assessment and diagnostic evaluation of patients undergoing contrast-enhanced urosonography in the setting of vesicoureteral reflux confirmation. Clin Hemorheol Microcirc. 2021;79(1):65–72.
40. McMahon CJ, Ayres NA, Bezold LI, Lewin MB, Alonzo M, Altman CA, et al. Safety and efficacy of intravenous contrast imaging in pediatric echocardiography. Pediatr Cardiol. 2005;26(4):413–7.
41. Miyamoto Y, Ito T, Takada E, Omoto K, Hirai T, Moriyasu F. Efficacy of Sonazoid® (perflubutane) for contrast-enhanced ultrasound in the differentiation of focal breast lesions: phase 3 multicenter clinical trial. AJR Am J Roentgenol. 2014;202(4):W400-7.
42. Morel DR, Schwieger I, Hohn L, Terrettaz J, Llull JB, Cornioley YA, et al. Human pharmacokinetics and safety evaluation of SonoVue®, a new contrast agent for ultrasound imaging. Invest Radiol. 2000;35(1):80–5.
43. Nanda NC, Wistran DC, Karlsberg RP, Hack TC, Smith WB, Foley DA, et al. Multicenter evaluation of SonoVue® for improved endocardial border delineation. Echocardiography. 2002;19(1):27–36.
44. Nguyen SA, Burrowes DP, Merrill C, Wilson SR. Contrast-enhanced ultrasound in pregnancy. Abdom Radiol (NY). 2025;
45. Ntoulia A, Back SJ, Shellikeri S, Poznick L, Morgan T, Kerwood J, et al. Contrast-enhanced voiding urosonography (ceVUS) with the intravesical administration of the ultrasound contrast agent Optison®TM for vesicoureteral reflux detection in children: a prospective clinical trial. Pediatr Radiol. 2018;48(2):216–26.
46. Ntoulia A, Darge K, Thompson AA, Back SJ. Safety of Ultrasound Contrast Agents in Pediatric Patients With Sickle Cell Disease and Trait. J Ultrasound Med. 2024;43(1):189–200.
47. Nucifora G, Marsan NA, Siebelink HM, van Werkhoven JM, Schuijf JD, Schalij MJ, et al. Safety of contrast-enhanced echocardiography within 24 h after acute myocardial infarction. Eur J Echocardiogr. 2008;9(6):816–8.
48. Papadopoulou F, Ntoulia A, Siomou E, Darge K. Contrast-enhanced voiding urosonography with intravesical administration of a second-

generation ultrasound contrast agent for diagnosis of vesicoureteral reflux: prospective evaluation of contrast safety in 1,010 children. Pediatr Radiol. 2014;44(6):719–28.
49. Piscaglia F, Bolondi L, Aiani L, Luigi Angeli M, Arienti V, Barozzi L, et al. The safety of SonoVue®® in abdominal applications: Retrospective analysis of 23188 investigations. Ultrasound in Medicine and Biology. 2006;32(9):1369–75.
50. Piskunowicz M, Kosiak W, Batko T, Piankowski A, Połczyńska K, Adamkiewicz-Drożyńska E. Safety of Intravenous Application of Second-Generation Ultrasound Contrast Agent in Children: Prospective Analysis. Ultrasound in Medicine & Biology. 2015 Apr 1;41(4):1095–9.
51. Platts DG, Luis SA, Roper D, Burstow D, Call T, Forshaw A, et al. The safety profile of perflutren microsphere contrast echocardiography during rest and stress imaging: Results from an Australian multicentre cohort. Heart Lung and Circulation. 2013;22(12):996–1002.
52. Riggs B.J., Martinez-Correa S., Stern J., Tierradentro-Garcia L.O., Haddad S., Anupindi S., et al. Intravenous administration of ultrasound contrast to critically ill pediatric patients. Pediatric Radiology. 2024;54(5):820EP – 830.
53. Sauer A, Wirth C, Platzer I, Neubauer H, Veldhoen S, Dierks A, et al. Off-label-use of sulfur-hexafluoride in voiding urosonography for diagnosis of vesicoureteral reflux in children: A survey on adverse events. World J Clin Pediatr. 2017;6(1):52–9.
54. Schwarze V, Marschner C, Negrão de Figueiredo G, Rübenthaler J, Clevert DA. Single-Center Study: Evaluating the Diagnostic Performance and Safety of Contrast-Enhanced Ultrasound (CEUS) in Pregnant Women to Assess Hepatic Lesions. Ultraschall Med. 2020;41(1):29–35.
55. Schwarze V, Froelich MF, Marschner C, Knösel T, Rübenthaler J, Clevert DA. Safe and pivotal approaches using contrast-enhanced ultrasound for the diagnostic workup of non-obstetric conditions during pregnancy, a single-center experience. Archives of Gynecology and Obstetrics. 2021;303(1):103–12.
56. Seelbach J, Krüger PC, Waginger M, Renz DM, Mentzel HJ. Safety and parents' acceptance of ultrasound contrast agents in children and adolescents - Contrast enhanced voiding urosonography and contrast enhanced ultrasound. Medical Ultrasonography. 2022;24(1):27–32.
57. Senior R, Andersson O, Caidahl K, Carlens P, Herregods MC, Jenni R, et al. Enhanced left ventricular endocardial border delineation with an intravenous injection of SonoVue®, a new echocardiographic contrast agent: A European multicenter study. Echocardiography. 2000;17(8):705–11.
58. Shaikh K, Chang SM, Peterson L, Rosendahl-Garcia K, Quinones MA, Nagueh SF, et al. Safety of Contrast Administration for Endocardial Enhancement During Stress Echocardiography Compared With Noncontrast Stress. American Journal of Cardiology. 2008;102(11):1444–50.

59. Shang Y, Xie X, Luo Y, Nie F, Jing X, Liao J, et al. Safety findings after intravenous administration of sulfur hexafluoride microbubbles to 463,434 examinations at 24 centers. Eur Radiol. 2023;33(2):988–95.
60. Siomou E, Giapros V, Serbis A, Makrydimas G, Papadopoulou F. Voiding urosonography and voiding cystourethrography in primary vesicoureteral reflux associated with mild prenatal hydronephrosis: a comparative study. Pediatric Radiology. 2020;50(9):1271–6.
61. Soman P, Lahiri A, Senior R. Safety of an intravenous second generation contrast agent in patients with severe left ventricular dysfunction [1]. Heart. 2000;84(6):634–5.
62. Stenzel M. Intravenous contrast-enhanced sonography in children and adolescents - a single center experience. J Ultrason. 2013;13(53):133–44.
63. Suma V, Seetharam K, Halazun H, Ziad S, Silbiger JJ, Bewley B, et al. Safety of perflutren-based echocardiographic contrast agent administration in the setting of a patent foramen ovale (pfo): a multicenter study conducted in tertiary care centers based in the new york/chicago metropolitan areas. JACC. 2017 Mar 21;69(11_Supplement):1418–1418.
64.Timperley J, Mitchell AR, Thibault H, Mirza IH, Becher H. Safety of contrast dobutamine stress echocardiography: a single center experience. J Am Soc Echocardiogr. 2005;18(2):163–7.
65.Torres A, Koskinen SK, Gjertsen H, Fischler B. Contrast-enhanced ultrasound using sulfur hexafluoride is safe in the pediatric setting. Acta Radiol. 2017;58(11):1395–9.
66. Wei K, Main ML, Lang RM, Klein A, Angeli S, Panetta C, et al. The effect of Definity® on systemic and pulmonary hemodynamics in patients. J Am Soc Echocardiogr. 2012;25(5):584–8.
67. Wei K, Mulvagh SL, Carson L, Davidoff R, Gabriel R, Grimm RA, et al. The safety of Definity® and Optison® for ultrasound image enhancement: a retrospective analysis of 78,383 administered contrast doses. J Am Soc Echocardiogr. 2008;21(11):1202–6.
68. Wei K, Shah S, Jaber WA, DeMaria A. An observational study of the occurrence of serious adverse reactions among patients who receive Optison® in routine medical practice. J Am Soc Echocardiogr. 2014;27(9):1006–10.
69. Wei K. Contrast echocardiography: what have we learned from the new guidelines? Curr Cardiol Rep. 2010 May;12(3):237–42.
70.Weiss RJ, Ahmad M, Villanueva F, Schmitz S, Bhat G, Hibberd MG, et al. CaRES (Contrast Echocardiography Registry for Safety Surveillance): a prospective multicenter study to evaluate the safety of the ultrasound contrast agent Definity® in clinical practice. J Am Soc Echocardiogr. 2012;25(7):790–5.
71. Wever-Pinzon O, Suma V, Ahuja A, Romero J, Sareen N, Henry SA, et al. Safety of echocardiographic contrast in hospitalized patients with

pulmonary hypertension: a multi-center study. Eur Heart J Cardiovasc Imaging. 2012;13(10):857–62.
72. Wong LS, Tse KS, Fan TW, Kwok KY, Tsang TK, Fung HS, et al. Voiding urosonography with second-generation ultrasound contrast versus micturating cystourethrography in the diagnosis of vesicoureteric reflux. Eur J Pediatr. 2014;173(8):1095–101.
73. Wu M, Fields JJ, Sachdev V, Belcik JT, Chen J, Reed F, et al. Increased Susceptibility for Adverse Reactions to Ultrasound Enhancing Agents in Sickle Cell Disease. J Am Soc Echocardiogr. 2023;36(2):208–15.
74. Zhang W, Cai B, Zhang X, Zhou J, Qiu L, Yi H. Contrast-enhanced voiding urosonography with intravesical administration of ultrasound contrast agent for the diagnosis of pediatric vesicoureteral reflux. Exp Ther Med. 2018;16(6):4546–52.
